# *De novo* Design of G Protein-Coupled Receptor 40 Peptide Agonists for Type 2 Diabetes Mellitus Based on Artificial Intelligence and Site-Directed Mutagenesis

**DOI:** 10.3389/fbioe.2021.694100

**Published:** 2021-06-14

**Authors:** Xu Chen, Zhidong Chen, Daiyun Xu, Yonghui Lyu, Yongxiao Li, Shengbin Li, Junqing Wang, Zhe Wang

**Affiliations:** ^1^Department of Pathology, The Eighth Affiliated Hospital, Sun Yat-sen University, Shenzhen, China; ^2^School of Pharmaceutical Sciences (Shenzhen), Sun Yat-sen University, Shenzhen, China

**Keywords:** T2DM, GPR40, artificial intelligence, oligopeptides, molecular fingerprint, site-directed mutagenesis

## Abstract

G protein-coupled receptor 40 (GPR40), one of the G protein-coupled receptors that are available to sense glucose metabolism, is an attractive target for the treatment of type 2 diabetes mellitus (T2DM). Despite many efforts having been made to discover small-molecule agonists, there is limited research focus on developing peptides acting as GPR40 agonists to treat T2DM. Here, we propose a novel strategy for peptide design to generate and determine potential peptide agonists against GPR40 efficiently. A molecular fingerprint similarity (MFS) model combined with a deep neural network (DNN) and convolutional neural network was applied to predict the activity of peptides constructed by unnatural amino acids (UAAs). Site-directed mutagenesis (SDM) further optimized the peptides to form specific favorable interactions, and subsequent flexible docking showed the details of the binding mechanism between peptides and GPR40. Molecular dynamics (MD) simulations further verified the stability of the peptide–protein complex. The R-square of the machine learning model on the training set and the test set reached 0.87 and 0.75, respectively; and the three candidate peptides showed excellent performance. The strategy based on machine learning and SDM successfully searched for an optimal design with desirable activity comparable with the model agonist in phase III clinical trials.

## Introduction

Type 2 diabetes mellitus (T2DM) is a degenerative disease caused by impairment in insulin action and pancreatic β-cell function, characterized by the inability to maintain glucose homeostasis. During the past two decades, T2DM has emerged as one of the most severe global healthcare concerns (Zheng et al., 2018). The general objective of all therapeutic modalities for T2DM is to decrease the circulating blood glucose levels. Currently, there are various types of drugs that play important roles in glycemic control. The mechanisms of action involve (i) insulin receptor ligands (insulin analogs), (ii) reduction of insulin resistance (biguanides and thiazolidinediones), (iii) stimulation of β-cells by insulin secretagogues (sulfonylureas and meglitinides), (iv) lowering postprandial blood glucose level via alpha-glucosidase inhibitors (acarbose and miglitol), and (v) blocking renal glucose reabsorption via a sodium-glucose cotransporter-2 (SGLT2) inhibitor (dapagliflozin) (Chatterjee et al., 2017). Although most treatments can manage glucose levels in T2DM patients, the progressive decline in β-cell function leads to inevitable dependence on exogenous insulin supply. Therefore, the novel mechanism and potent candidates should draw our attention in the treatment of T2DM (Chen et al., 2016).

G protein-coupled receptors (GPCRs) are among the most attractive membrane targets for many drugs (Sriram and Insel, 2018). In recent years, a growing number of GPCRs are being discovered with a wide variety of ligands, including free fatty acids, sucrose, acetate, lactate, and ketone bodies; and they are implicated in many pathophysiological functions (Blad et al., 2012). G protein-coupled receptor 40 (GPR40), also known as free fatty acid receptor 1 (FFAR1), is a member of the long-chain fatty acid GPCR family. Current studies proved that GPR40 plays a pivotal role in the potentiation of glucose-dependent insulin secretion from pancreatic β-cells, and it also regulates glucagon-like peptide 1 (GLP-1) and gastric inhibitory peptide (GIP) secretion (Li et al., 2020). The view that the agonists of GPR40 may be beneficial for treating T2DM is substantially studied (Nutan et al., 2017). Clinical candidates targeting GPR40 to enhance insulin secretion have been reported in the literature (Naik et al., 2013). Moreover, GPR40 agonists have been described as having superior effects, including cardioprotection, suppressing glucagon levels, and weight loss, than other hypoglycemic drugs (Mancini and Poitout, 2015).

The current development of GPR40 agonists primarily focuses on small molecules, whose limitations, such as low selectivity, high toxicity, and low efficiency, can contribute to failures in clinical trials (Naik et al., 2012; Kim et al., 2018). Therefore, new entities should draw our attention to the development of GPR40 agonists. In this regard, peptides show high bioactivity associated with high specificity, and low toxicity has made them attractive therapeutic agents. Thus, the development of synthetic peptides is an attractive modality to active GPR40. However, little is known on the process of discovery in peptides for targeting GPR40, partly because the core binding pocket is inaccessible to the natural peptides. To this end, peptidomimetic composed of unnatural amino acids (UAAs) can significantly increase its structural diversity and improve binding affinity and selectivity toward GPR40. Besides, the blood circulation time is important for the duration of action. In this context, peptide drugs can provide tunable circulation times through extended sequence engineering or drug delivery systems (Zorzi et al., 2017; Wang et al., 2018, 2020). In the case of membrane protein, the binding site of GPR40 was located in the extracellular part, so peptides targeting GPR40 can act without membrane penetration.

In response to the rapidly growing demand for binding, functional, and ADMET (absorption, distribution, metabolism, and excretion) information of many drug-like bioactive compounds, various public databases (e.g., DrugBank, PubChem, ChEMBL, and Zinc) have been developed for drug discovery. Quantitative structure–activity relationships (QSAR) modeling can largely increase drug design efficiency (Krishna and Anuradha, 2020), but how to build QSAR models for GPR40 remains elusive. Recently, the artificial intelligence (AI) method, deep neural network (DNN) algorithm, has been well represented as a novel approach to build QSAR models (Hessler and Baringhaus, 2018). The use of deep learning in chemical discovery has received considerable attention in recent years. For example, DNN predicted cell permeability based on the chemical structure of organic fluorescent (Soliman et al., 2021), and the convolution neural network (CNN) was used to find the relationship between the chemical structure of odor molecules and their related odors (Sharma et al., 2021). The graph convolutional neural network (GCN) successfully predicted the reverse synthesis reaction (Ishida et al., 2019), and the transformer neural network directly designed potential ligand molecules based on the sequence of the protein target (Grechishnikova, 2021). DNN had achieved many successes in molecular descriptor-based tasks and had the advantage of being easy to construct (Li et al., 2018; Cai et al., 2019). The commonly used two-dimensional (2D) CNN had made brilliant achievements in computer vision, and the derived one-dimensional (1D) CNN was suitable for sequence data, such as gene sequence and natural language processing (Esteva et al., 2021; Sun et al., 2021; Zhang et al., 2021). Molecular descriptors could be used as input of DNN to construct QSAR models, and quantified molecular descriptors could be regarded as a unique sequence for 1D CNN to use. Nevertheless, there was no perfect AI model, and performance comparison between models on a specific task can help obtain more accurate results (Yang et al., 2019).

**FIGURE 1:**
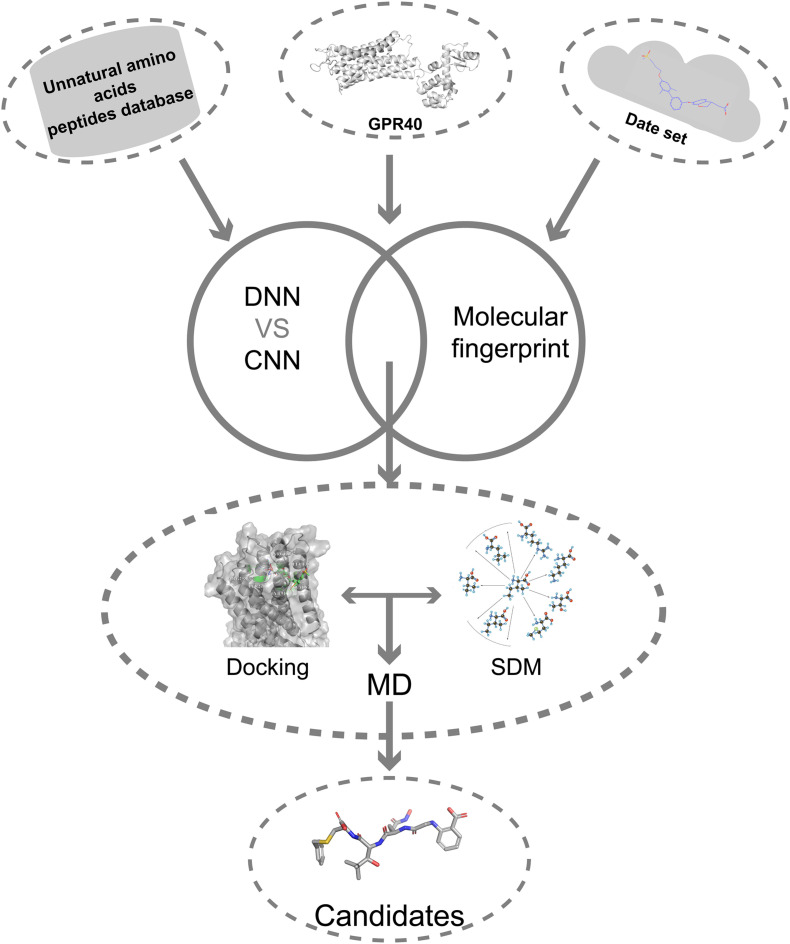
The overall flowchart.

Here, we describe a novel AI combining mutation scanning approach to design new promising oligopeptide agonists for GPR40 (Figure 1). Initially, the ChEMBL database (version 28) was used to extract 528 compounds with known bioactivity and structural profiles. A QSAR model was constructed based on DNN. The molecular fingerprint search method was employed to screen active chemical entities among the oligopeptides generated from the library of natural amino acids (AAs)/UAAs. The obtained candidate oligopeptides were then evaluated for the binding affinity to GPR40 through flexible docking procedures. The initial lead oligopeptides were further processed by the site-directed mutagenesis (SDM) optimization to achieve potential hits toward GPR40. The peptides were further analyzed in molecular dynamics (MD) simulations and showed good stability. These top candidates might be up-and-coming for the treatment of T2DM.

## Materials and Methods

### Data Set

A total of 2,084 GPR40 agonists with EC50 measurements have been extracted from the ChEMBL database (Mendez et al., 2018). The unit of measurement for EC50 is nmol. Fasiglifam (TAK-875), a potent GPR40 selective agonist withdrawn from phase III clinical trials due to drug-induced hepatotoxicity (Yabuki et al., 2013), was used as a positive control to evaluate the efficacy profile of the obtained TAK-875 analogs. In this study, only the compounds that interact with the same region in the TAK-875 binding site were kept, and all duplicate entries were removed. The final data set that consisted of 528 molecules is listed in Supplementary Table 1. All molecular structures were constructed in molecular operating environment (MOE) version 2019.0102 and imported into the database (Vilar et al., 2008). Structure preparation was performed on all molecules to repair structural errors and add hydrogen atoms. Protonate3D was used to protonate all molecules. The temperature of Protonate3D was set to 300K, the pH was set to 7, and the ion concentration was set to 0.1 mol/L. Energy minimization was performed for each molecule to obtain the optimal configuration. The root mean square gradient of energy minimization was set to 0.1 kcal/mol/A.

### Feature Calculation

A total of 153 2D molecular descriptors were calculated in MOE. The names of all molecular descriptors and their specific meanings are listed in Supplementary Table 2. The EC50 value of each molecule is logarithmically processed to pEC50 (Eq. 1).

(1) $$p\,E\,C_{50}=-\log E\,C_{50}+9$$

**FIGURE 2:**
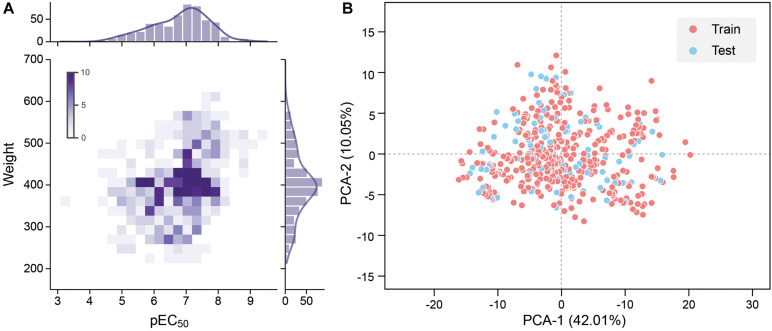
**(A)** Molecular weight and pEC50 distribution of the data set. Frequency increases with the color from light to dark. **(B)** 2D principal component analysis (PCA) results of the data set. The red dot is the training set, and the blue dot is the test set.

To improve the model accuracy, Morgan fingerprints with a length of 1,024-bit strings were calculated by the RDKit toolkit to compare the performance with the MOE 2D descriptors Landrum (2016). All quantitative features were standardized. The pEC50 of 528 molecules in the data set is typically in the range between 4 and 9, and the molecular weight is roughly between 200 and 600 (Figure 2A).

### The UAA Library and the Oligopeptide Database

The AA database used in this study contains 20 natural AAs and 850 UAAs derived from 20 canonical AAs. All UAA category information is in Supplementary Table 3. The number of peptide residues is limited to 3–5. The molecular weight of the peptide is similar to the data set molecule, which helps to improve the accuracy of peptide activity prediction and facilitates the peptide’s entry into the GPR40 binding site. Three random-constructed virtual peptides (tripeptides, tetrapeptides, and pentapeptides) libraries were generated, where each peptide library consists of 100,000 peptide entries. The generation and preservation of peptide structures were done by MOE’s SVL script.

### Model Training

**FIGURE 3:**
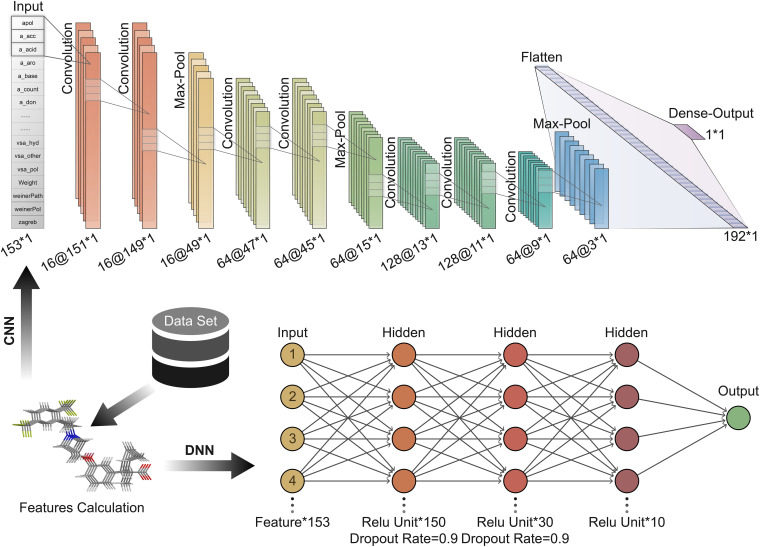
Deep learning architecture of convolution neural network (CNN) and deep neural network (DNN) used to predict peptide activity.

Convolution neural network is a feedforward neural network that includes convolution calculations (LeCun et al., 2015). It can learn features from data and generalize them to specific data. CNN has achieved great success in speech recognition, image recognition, and other fields. The molecular features calculated based on MOE can be expressed as a 1 × 153 vector form; hence 1D CNN can be well adapted to the task of predicting the activity of oligopeptides based on molecular features. In contrast, the DNN is a fully connected neural network, which is more prone to overfitting than CNN. The hyperparameters of the neural network are important for the training and accuracy of the model. We repeatedly adjusted and confirmed the best hyperparameters in the training process. A CNN with seven convolutional layers, three max-pooling layers, and one Flatten layer is built (Figure 3). The size of the local receptive field was 1 × 3; and the number of feature maps of the convolutional layer was 16, 16, 64, 64, 128, 128, and 64 in order. The filter size of the pooling layer was 3. The Flatten layer was used to expand the output of the pooling layer and connect to the final dense layer. The batch size was set to 10.

Here, DNN contains three hidden layers (Figure 3). The number of units contained in each hidden layer is 150, 30, and 10 in order. The first and second hidden layers applied Dropout to prevent overfitting, and the Dropout Rate was defined as 0.9.

The rectified linear unit was used as the activation function of CNN and DNN, mean square error (MSE) (Eq. 2) was defined as the loss function, and the Adam optimizer was used to minimize the loss function (Nair and Hinton, 2010; Kingma and Ba, 2014). The initial learning rate of the Adam optimizer was set to 0.001 and 0.0008 in CNN and DNN, respectively.

(2) $$\mathrm{Mean}\,\mathrm{Square}\,\mathrm{Error}=\frac{1}{m}\,\sum_{i=1}^{m}{\left(y_{i}-\hat{y_{i}}\right)}^{2}$$

where *m* is the sample size, *y_i* is the actual value of the sample’s pEC50, and $\hat{y_{i}}$ is the predicted value of the sample by the model. The data set was divided into a training set and a test set according to the ratio of 4:1. Principal component analysis (PCA) was performed with all the features as input, and the features were mapped to two principal components with 42.01 and 10.05% variances. The data set shows high chemical diversity. 2D PCA shows that the training set and the test set are located in the same chemical space (Figure 2B). All input features in CNN and DNN had undergone maximum and minimum standardization processing. The last layer of CNN and DNN did not use the activation function and outputs pEC50. R-square was used to evaluate the accuracy of model fitting (Eq. 3).

(3) $$R-\text{square}=1-\frac{\sum_{i}{\left(\hat{y_{i}}-\bar{y_{i}}\right)}^{2}}{\sum_{i}{\left(y_{i}-\bar{y_{i}}\right)}^{2}}$$

where $\hat{y_{i}}$ is the predicted value of the sample by the model, *y_i* is the actual pEC50 value, and $\bar{y_{i}}$ is the mean pEC50.

### Principal Component Analysis and LASSO Feature Selection

To improve the accuracy of the model, speed up model training, and reduce overfitting, PCA and LASSO feature selection were applied to reduce redundant features (Tibshirani, 2011). The data set was reduced in dimensionality by solving the covariance matrix. The variance accumulation threshold of PCA was set to 0.99 to remove low variance features. LASSO regression can compress the coefficients of the regression variables to 0 and make the parameters become sparse because it has L1 regularization terms. Here, the alpha value of the LASSO regression estimator was set to 0.001 to limit the L1 regular term, and the maximum number of iterations was set to 8,000 to fit all features.

### Molecular Similarity Search

The fundamental assumption in drug discovery is that structurally similar molecules exhibit similar biological activities (Muegge and Mukherjee, 2016). Hence, the molecular fingerprint method allows for ligand-based virtual screening and predicts bioactivity or other properties of candidates that have not been tested with the fingerprint model built from the database of the active template. In simple terms, fingerprint models transfer the structure of active templates and candidates simultaneously into numbers or matrices and compare them with mathematical methods, for example, the Tanimoto coefficient (Cereto-Massagué et al., 2015). According to the design principle of the fingerprint system, different fingerprint schemes bring out different molecular attributes. For instance, the description of GpiDAPH3 is “3-point pharmacophore-based fingerprint calculated from the 2D molecular graph. Each atom is given one of 8 atom types computed from 3 atomic properties: “in pi system,” “is donor,” “is acceptor.” Anions and cations are not represented. Then, all triplets of atoms are coded as features using the three graph distances and three atom types of each triangle. The resulting fingerprint is represented as a sparse feature list.”

Nearly all common fingerprint types, like BIT_MACCS, MACCS, TAD, TAT, and GpiDAPH3, are available in MOE, so the most proper type should be designed first. It has been proved that there is no generally superior fingerprint to be used (Muegge and Mukherjee, 2016), Thus, in this study, we initially used the GpiDAPH3 fingerprint model to determine which score function (Must match, Maximum, Minimum, and Average and Distance) is suitable for this condition. With the most suitable score function, fingerprint types were then tested for the superior fingerprint type in our database. Having a certain score function and fingerprint type, we trained our fingerprint model to predict the bioactivity of candidates. The data set of molecular fingerprint model consisted of 528 molecules, which were as identical as DNN and CNN models.

### Structure Preparation and Molecular Docking

The crystal structure of the human GPR40-TAK-875 complex (PDB:4PHU) was obtained from Protein Data Bank to perform the docking study (Berman et al., 2000; Srivastava et al., 2014). The structure preparation, Protonate3D, and energy minimization were carried out by MOE. The structure preparation repaired the gap in the protein structure. Protonate3D added hydrogen atoms to the structure and completed the protonation in the default ionization state. The terminal amide, sulfonamide, and imidazole groups were able to flip to optimize the hydrogen bond network. All water molecules were removed. The atomic restraint strength was set to 10, and the extent of the flat bottom of the flat-bottom restraint was set to 0.25 to prevent excessive positional deviation of the atoms. The energy minimization with a root mean square gradient of 0.1 kcal/mol/A was performed to optimize protein conformation. The binding site of GPR40 was created according to the TAK-875 (partial agonist) in crystal complexes.

The molecular docking was performed by the MOE. The same energy minimization protocol as the protein structure was performed on the peptides. Peptides were placed at the binding site by the method of Triangle Matcher. The 30 postures generated by the placement method for each peptide would be refined in the Amber10:EHT force field using the Induced Fit method (Gerber and Müller, 1995; Case et al., 2008). Generalized Born/volume integral/weighted surface area (GBVI/WSA) dG Scoring is used to evaluate the binding free energy of the docking result (Eq. 4).

(4) $$△\,G\approx c+\alpha \,\left[\frac{2}{3}\,\left(△\,E_{C\,o\,u\,l}+△\,E_{s\,o\,l}\right)+△\,E_{v\,d\,W}+\beta \,△\,S\,A_{w\,e\,i\,g\,h\,t\,e\,d}\right]$$

where *c* is the average gain and loss of rotation and translation entropy, α; β is the constant determined during the function training process; *E*_Coul_ is the Coulomb electrostatic term; *E*_sol_ is the solvation electrostatic term calculated by GB/VI solvation model; *E*_vdW_ is van der Waals contribution; and *SA*_weighted_ is surface area weighted by exposure.

### Site-Directed Mutagenesis and Re-Dock

Amino acid single-point mutations were applied in candidate oligopeptides to improve their affinity and stability by attempting to form the salt bridge equivalent to TAK-875. The selected UAAs were sequentially substituted to the designated positions following the reassessment of the interaction between the updated peptide and GPR40. New peptides were isolated from the complex structure created by the mutation. The peptide conformation was preserved, and re-docking to GPR40 for refinement and for further rescoring the GPR40–peptide interactions was performed.

### Molecular Dynamics Simulations

Further analysis of stability about the GPR40–candidate peptides complexes was conducted by MD simulations with GROMACS 2020.5 software on Manjaro platform after flexible docking (Abraham et al., 2015). The topology files of candidate peptides, an indispensable component in MD, were obtained from the Swissparam webserver (Zoete et al., 2011). The GPR40–peptides complexes were firstly placed in a periodic cubic box (1.2-nm edge), and then the TIP3P water molecules and 0.145 M of NaCl ions were added. The steepest descent minimization algorithm with 5,000 steps at most was conducted to avoid excessive local stress. Before the formal simulation, 1-ns NVT pre-equilibrium and 1-ns NPT pre-equilibrium were done to stabilize the temperature (310K) and pressure (1 bar). Formal simulation lasted 10 ns, and the CHARMM27 was applied (Brooks et al., 2009). The analysis of the dynamics simulations consisted of root mean square deviation (RMSD), mean square displacement (MSD), root mean square fluctuation (RMSF), solvent-accessible surface areas (SASAs), radius of gyration (gyrate), and system energy.

## Results and Discussion

### Comparison of CNN and DNN

**FIGURE 4:**
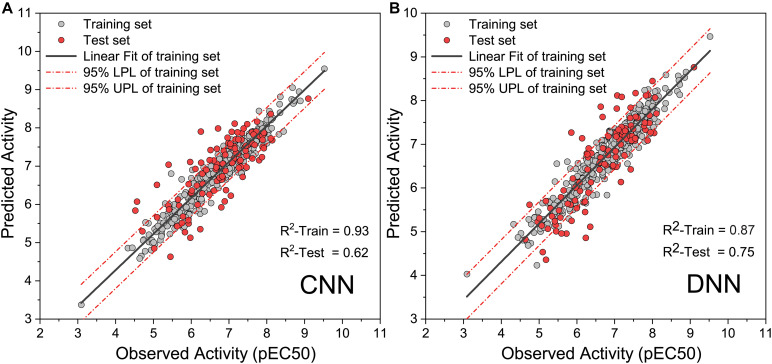
Observed activity and predicted activity of training set and test set using **(A)** The deep neural network (DNN) and **(B)** The convolution neural network (CNN).

To obtain high-precision activity prediction results, CNN and DNN were constructed to compare their fitness with the task. Both neural networks were trained based on all molecules in the data set. All molecular features were normalized with Min–Max scaling before input into the neural network to improve training efficiency. The CNN was trained for 100 epochs with a batch size of 10. In the ninth epoch, the MSE of the training set and the test set was reduced to 0.0084 and 0.0090, respectively. Then the MSE begins to fluctuate slightly, the MSE of the training set continues to decline, and the MSE of the test set fluctuates within a certain range (Supplementary Figure 1). When training to the last 10 epochs, the MSE of the training set dropped from 0.0020 to 0.0010, and the MSE of the test set fluctuated between 0.0089 and 0.0110. DNN traverses all samples in each step, and 1,000 steps of training were executed. At the 270th step, the MSE on the training set and the test set was reduced to 0.0018 and 0.0051, respectively. The training set MSE had been declining throughout the training process. The MSE of the test set begin to rise slowly after the 270th step, and it becomes stable at the end of training (Supplementary Figure 2). During the last 100 steps, the MSE of the training set fluctuates slightly around 0.0004, and the MSE of the test set fluctuates around 0.0083. When compared with DNN, CNN has a greater loss function oscillation, and the ability to reduce the loss function is worse than DNN. Yet on the test set, the highest R-square of DNN reaches 0.750 (Figure 4A), while that of CNN is only 0.620 (Figure 4B). DNN was shown to outperform CNN on the task of activity prediction. Nevertheless, both neural networks exhibit slight overfitting; thus, the early stopping strategy was applied to DNN.

### Performance of Features

**FIGURE 5:**
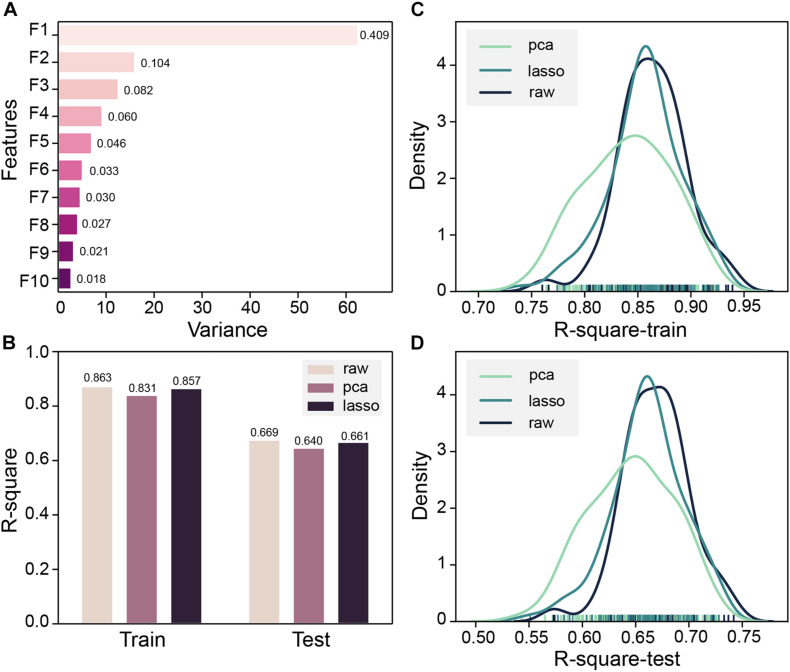
**(A)** Top 10 principal component analysis (PCA) features and their variance ratios. **(B)** R-square average of 100 trainings of deep neural network (DNN) based on PCA, LASSO, and original features on the training set and test set. **(C)** Train set R-square distribution of DNN based on PCA, LASSO, and original features in 100 trainings. **(D)** The test set R-square distribution of DNN based on PCA, LASSO, and original features in 100 trainings.

Both MOE 2D features and Morgan fingerprints features were input to DNN for training. To ensure that the model converges, DNN trained 2000 steps on the Morgan fingerprint data set. At the 1,250th step, the MSE of the training set and the test set both drop to 0.02 and stabilized thereafter (Supplementary Figure 3). The DNN based on Morgan fingerprint training exhibits a more gradual decline in MSE than the DNN based on MOE 2D feature training. The test set MSE of the Morgan fingerprint model was stable at 0.02, which was much higher than the MSE of MOE 2D feature model, and the Morgan fingerprint model did not achieve good accuracy. Subsequently, based on the MOE 2D features, we attempted to optimize the features through PCA and LASSO feature selection to improve the accuracy of the model. Z-score standardization was performed on the data set, the cumulative variance threshold was set to 0.99, and the 153-dimensional features had been reduced by PCA to generate 43 new features. The variance of the top 10 new features is shown in Figure 5A. The fitting accuracy of the LASSO regression estimator reaches 0.71, and there are 83 features whose coefficients are not 0 after fitting (Supplementary Table 4). The new features obtained by the two methods were input to DNN for 100 pieces of training, and each training contains 1,000 steps. The R-square of the model training set and the test set would be checked every 10 steps, and the largest R-square that satisfied the difference between the training set R-square and the test set R-square less than 0.2 in 1,000 steps would be recorded. The R-square frequency distribution curves of the training set and test set obtained by 100 trainings on the original feature data set, PCA dimensionality reduction feature data set, and LASSO feature selection data set are shown in Figures 5C,D. The performance of new features obtained by PCA dimensionality reduction was relatively poor and produced more low-precision models. After the removal of low-importance features via LASSO feature selection, the model still showed good accuracy. Still, the overall trend of model accuracy has a slight decrease as compared with the original data (Figures 5B–D). LASSO feature selection improved the model training speed and only sacrificed a little model accuracy. This method could effectively improve training efficiency when training on an extensive data set. To obtain the highest precision model, we still used raw data for training regardless of training speed. Finally, the DNN model with R-squares of 0.87 and 0.75 for the training set and test set obtained through the early stopping strategy was retained.

### Molecular Fingerprint Similarity

All the fingerprint models were built in MOE. For the most accurate and suitable fingerprint model in this situation, the score function and type of fingerprint model must be tested. To evaluate different fingerprint models better, the training set and the testing set were carefully designed. All the training set in fingerprint models consisted of active entities (low EC50 in ChEMBL) since the purpose of the fingerprint model is to exploit novel potential active entities in other databases. As for the testing set, the active entities (low EC50 in ChEMBL) were encoded index1-50, and on the contrary, the inactive entities (high EC50 in ChEMBL) were encoded index51-100. Since all of the fingerprint models were built from active entities, a higher similarity score represents more possibilities of being an active entity. Therefore, an ideal model should be available to distinguish active entities from inactive entities. To this end, the entities index1-50 should be with a high similarity score, and index51-100 should be with a low similarity score. Furthermore, the *k*, slope of the regression line, which is the relationship between similarity score and index, can reflect the quality of the model in some distance. When the absolute value of *k* is high, it means that there is a tendency of the decrease in similarity score with the increase of index, which also matches with an ideal model.

**FIGURE 6:**
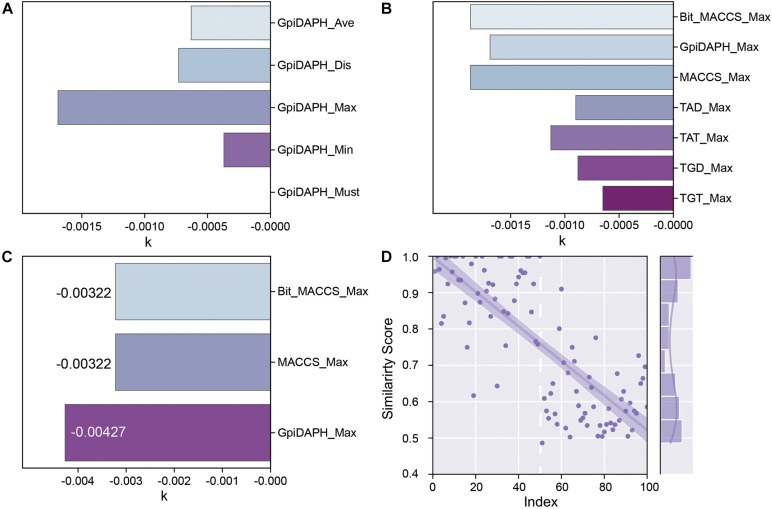
**(A)** The *k* values of the fingerprint model when using different score functions. **(B)** The *k* values of each type of fingerprint model when using the Maximum scoring function. **(C)** The *k* values of Bit_MACCS, MACCS, and GpiDAPH on the new training set. **(D)** Similarity scores by GpiDAPH fingerprint model of active (index1-50) and inactive (index51-100) molecules.

GpiDAPH, a common and gorgeous fingerprint model, was chosen to evaluate which score function (Average, Distance, Maximum, Minimum, and Must match) is the most appropriate one. The predicted similarity score and index of testing data are shown in Supplementary Figure 4, and the values of k are shown in Figure 6A. Comparing the value of k and the distribution of the similarity score, the score function of maximum is chosen as the best score function in this research (Supplementary Figure 4C).

The types of fingerprint models were then evaluated with the same method. The result is shown in Figure 6B and Supplementary Figure 5. The value of k shows little difference among Bit_MACCS, GpiDAPH, and MACCS, so the suitable type cannot be designed yet. Therefore, the entities with higher bioactivity (EC50) were chosen to consist new training set, and the testing set was accordingly adjusted. The new training set and testing set are constructed to evaluate these three fingerprint types further, and the result is shown in Figure 6C and Supplementary Figure 6. The GpiDAPH fingerprint model shows the best performance in distinguishing between active and inactive molecules and is thus the chosen type in this study (Figure 6D and Supplementary Figure 6B). Unlike other molecular fingerprint models, in Figure 6D, the similarity values of active entities (index1-50) are mainly located in the upper left corner, while the inactive entities (index51-100) are in the bottom right corner.

### Activity Prediction of Peptides and AA Preferences

**FIGURE 7:**
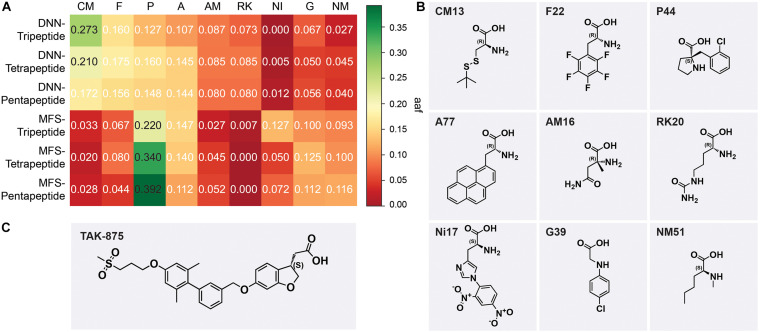
**(A)** The aaf of MSF and deep neural network (DNN) on three peptides. **(B)** The most frequent amino acids from nine preferred amino acids. **(C)** The chemical scaffold of TAK-875.

Deep neural network model and molecular fingerprint similarity (MFS) were used to evaluate the performance of oligopeptides. All peptides were sorted by evaluation scores via two models. The output results of the model were divided into six groups according to the model and peptide length, namely, DNN-Tripeptide, DNN-Tetrapeptide, DNN-Pentapeptide, MFS-Tripeptide, MFS-Tetrapeptide, and MFS-Pentapeptide. The top-ranked 50 oligopeptides in each group were selected as samples for AA frequency statistics to observe the AA preference of high-scoring peptides. AAs were classified according to the backbone for frequency statistics, and the AA frequency (*aaf*) of each sample was calculated according to Eq. (5).

(5) $$a\,a\,f=\frac{C\,A}{T\,A}$$

where *CA* is the frequency of a type of AA that appears in a sample and *TA* is the total number of AAs contained in a sample. The top 5 types of AAs ranked by *aaf* in each sample were selected and integrated. The favorable AAs determined by the DNN model were cysteine and methionine derivatives (CM), phenylalanine derivatives (F), proline derivatives, alicyclic AAs (P), alanine derivatives (A), α-methyl AAs (AM), and lysine and arginine derivatives (RK). The favorable AAs determined by MFS were P, A, F, nitro, and dinitrophenyl AAs (NI), glycine derivatives (G), and *N*-methyl AAs (NM). The most frequently occurring AAs among the nine preferred AAs show the backbone of this type of AA (Figure 7B). DNN’s most preferred AA is CM, and CM had *aaf* of 0.273 in the DNN-Tripeptide sample (Figure 7A). Similarly, CM also achieved higher *aaf* (0.210 and 0.172, respectively) in DNN-Tetrapeptide and DNN-Pentapeptide. The most preferred AA class of MFS was P. The *aaf* of P in MFS-Pentapeptide reached 0.392, which was the highest value among all samples. P reaches high *aaf* of 0.220 and 0.340 in MFS-Tripeptide and MFS-Tetrapeptide, respectively (Figure 7A). DNN and MFS both showed a preference for P and A, but DNN showed a lower tendency for NI, G, and NM, which were preferred by MFS. DNN was more stable than MFS in the *aaf* of three different length peptides. Most of the molecules in the data set used to construct the model are analogs of TAK-875, all of which contain ring structures, and many analogs contain sulfone groups, sulfur heterocycles, or carboxyl groups (Figures 7B,C). The two AAs categories including P and A that DNN and MFS commonly prefer were molecules with five-membered heterocyclic or aromatic rings. The most preferred CM AAs of DNN were cysteine and methionine derivatives, both of which contained sulfur groups. Compared with MFS, DNN successfully captured the sulfur group features of the data set molecules. We noticed that some molecules in the data set contained halogen elements. F22 and P44 are observed with the highest frequency among the F and P AAs preferred by DNN, which shows that DNN has paid attention to the halogen element features of the molecule (Figure 7B).

### Molecular Docking

**FIGURE 8:**
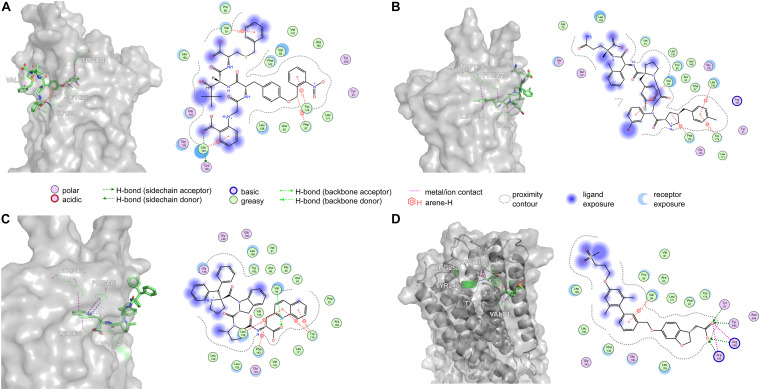
The top three poses generated by flexible docking and the pose of TAK-875 in the crystal structure after energy minimization. In 3D pose diagrams, the red dash lines represent ionic bonds, magenta dash lines represent π–H interaction, and blue dash lines represent H-bonds. **(A)** 4-15, **(B)** 5-1, **(C)** 4-4, and **(D)** TAK-875.

Amino acid preference statistics showed that MFS paid attention to molecular skeleton information, and DNN could pay attention to the details of the molecule. We combined the results of the two models to select candidate peptides for docking. The intersection of the top 1% (1,000) of the two models of the tripeptide, tetrapeptide, and pentapeptide ranked by MFS and DNN was selected. The intersection of peptide samples contains 39 tripeptides, 16 tetrapeptides, and 7 pentapeptides. The GBVI scoring function that estimates the free energy of ligand binding according to a given posture is used to assess docking posture (Naïm et al., 2007) (Table 1). The GBVI scores of the top three peptides numbered 4-15, 5-1, and 4-4 reached −10.2063, −9.62029, and −9.57896, respectively. As shown in Figure 8A, the 4-15 carboxyl group forms a hydrogen bond with the hydrogen on the backbone of Cys136. The three benzene rings of 4-15 form π–H interactions with Val81, Trp174, and Leu135. Phe87 forms a π–H interaction with the main chain hydrogen of 4-15. The docking pose of 5-1 is shown in Figure 8B. The benzene ring in 5-1 forms π–H interaction with Val84, Leu138, and Trp174. The benzene ring of Phe142 forms a π–H interaction with 5-1. The interaction between 4-4 and GPR40 is shown in Figure 8C. The quinoline ring of 4-4 forms 3 π–H interactions with Leu138 and Trp174. The benzene ring of Phe142 forms a π–H interaction with 4-4. The H-bond is found between Val84 and the carboxyl of 4-4. As a control, TAK-875 was also evaluated by the GBVI method. The crystal structure of TAK-875 has abnormal van der Waals interaction between atoms. The energy minimization was performed on the crystal structure to optimize the interaction within the structure. The repaired structure shows that the GBVI of TAK-875 has been reduced from −1.4554 to −9.7494. The pose of TAK-875 is shown in Figure 8D. To be specific, the carboxyl of TAK-875 formed a salt bridge with Arg183 and Arg258. The benzene ring in TAK-875 interacted with Val84 by forming π–H interaction. The binding of TAK-875 to GPR40 mainly relied on two strong salt bridges. The fixation of the three peptides at the binding site mainly relied on π–H interaction, hydrogen bonding, and van der Waals force. Compared with peptides, TAK-875 had a linear structure that allowed it to penetrate deeply into the binding pocket to form salt bridge interactions. In addition, the peptide exhibited higher solvent exposure than TAK-875 due to the residues of the peptide anchored on the outer surface of GPR40. The receptor solvent exposure of the peptide is also higher than that of TAK-875. The solvent exposure of the GPR40 residues after binding with the peptide is further reduced, indicating that the peptide binding to GPR40 is primarily dependent on hydrophobic interactions.

**TABLE 1:** GBVI (GBVI/WSA dG scoring) of the top 10 ranked peptides and TAK-875 in the docking results.

**Name**	**Ranking**	**GBVI**	**Sequence**
4-15	1	−10.2063	(G28)(NI42)(L18)(CM25)
5-1	2	−9.62029	(P80)(G35)(P40)(F117)(AM18)
4-4	3	−9.57896	(P131)(P107)(AM47)(A60)
4-10	4	−9.43213	(A109)(Y40)(CM28)(P103)
4-1	5	−9.38494	(P44)(G22)(L09)(F87)
5-2	6	−9.17657	(P95)(G32)(G34)(A96)(CM25)
3-12	7	−9.16672	(P93)(G28)(W026)
3-13	8	−9.04828	(P97)(A108)(P91)
5-3	9	−8.88673	(RK20)(G31)(L04)(P40)(W030)
4-5	10	−8.76586	(A109)(G19)(P52)(NM26)
TAK-875		−9.74940	

*GBVI/WSA, generalized Born/volume integral/weighted surface area.*

### Site-Directed Mutagenesis Optimization and Re-Docking Evaluation

**FIGURE 9:**
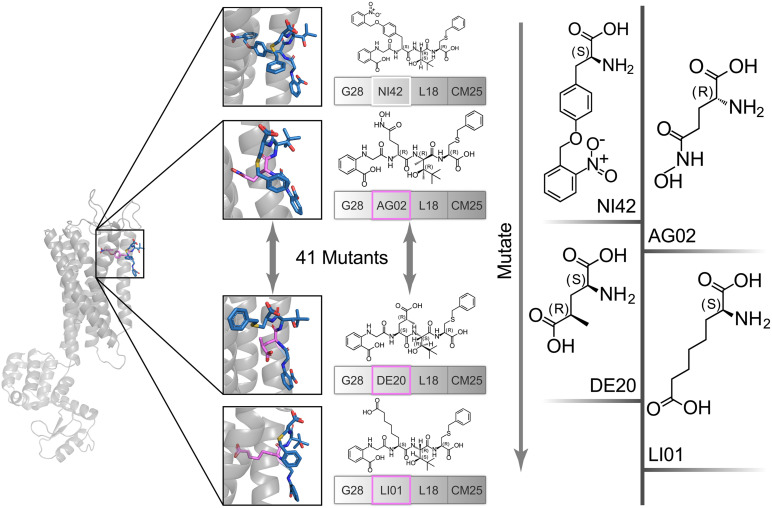
The NI42 amino acid of 4-15 was defined as the mutation site, and 41 mutations were performed.

**FIGURE 10:**
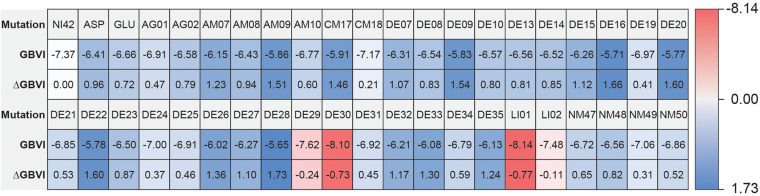
Site-directed mutagenesis result.

The high affinity of TAK-875 came from the ionic and hydrogen bonds between its carboxyl group and Arg183 and Arg258. The carboxyl group was at the end of the TAK-875 chain, and the chain is firmly inserted between the two α-helices (Figure 8D). The NI42 AA of 4-15 with the lowest GBVI enters the target site in a similar posture, but it fails to form strong ionic or hydrogen bonds (Figure 8A). To this end, the SDM method was carried out to optimize the performance of candidate peptides. NI42 that successfully penetrated into GPR40 is defined as the mutation site, and 41 AAs with a carboxyl chain similar to that of TAK-875 are used for SDM (Supplementary Table 5 and Figure 9). A rough mutation evaluation without the refinement of Amber10:EHT force field shows that Li01, DE30, DE29, and LI02 have better GBVI scores than NI42 (Figure 10). The method of directly replacing AAs did not use a force field to adjust the conformation, so all the 41 mutant conformations were preserved, and the flexible docking was performed again. The results show that the DE20 and AG02 mutants with low GBVI formed ionic and hydrogen bonds (Table 2).

**TABLE 2:** The results of re-dock and energy minimization of AG02, DE20, and LI01 mutants.

**Mutated amino acid**	**GBVI**	**Hydrogen/Ionic bond**
AG02	−8.8651	Yes
DE20	−8.8189	Yes
LI01	−8.4173	No
AG02 (energy minimization)	−10.043	Yes
DE20 (energy minimization)	−10.404	Yes
LI01 (energy minimization)	−9.1861	Yes

*GBVI, generalized Born/volume integral.*

**FIGURE 11:**
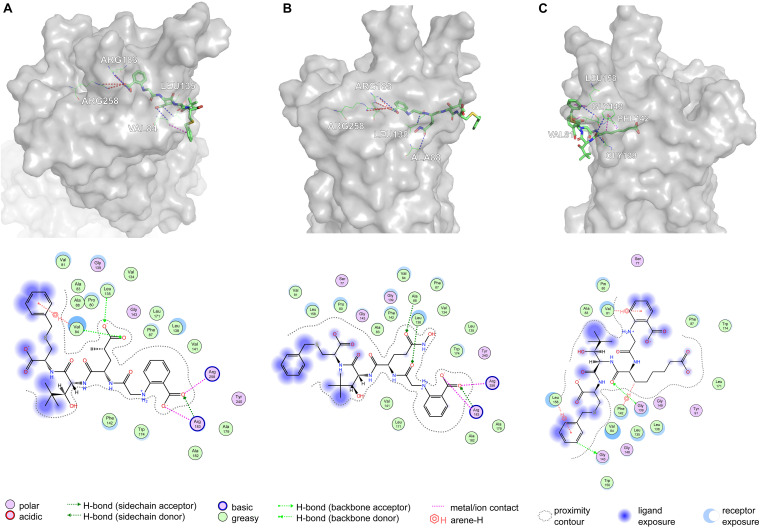
Poses are generated by the flexible docking of mutants. In 3D pose diagrams, the red dash lines represent ionic bonds, magenta dash lines represent π–H interaction, and blue dash lines represent H-bonds. **(A)** DE20 mutant, **(B)** AG02 mutant, and **(C)** LI01 mutant.

Based on Figure 11A, Arg183 and Arg258 also play a significant role in the interaction between DE20 mutant and GPR40 by forming the salt bridge or ionic interaction. The H-bonds were found between Leu135, Val84, and DE20 mutant. The benzene ring of the DE20 mutant forms a π–H interaction with Val84. In Figure 11B, the interaction between AG02 mutant and GPR40 is shown. Interestingly, the carboxyl moiety of AG02 mutant formed a salt bridge with Arg183 comparable with TAK-875. In addition, the AG02 mutant formed a strong ionic interaction with Arg258. The Ala88 and Leu138 interacted with AG02 mutant by H-bond. Less solvent exposure was observed on DE20 mutant and AG02 mutant similar to TAK-875. LI01 with the carboxyl group attached to the long alkyl chain showed the best performance in SDM but poor performance after re-docking. The re-docking pose of LI01 mutant in GPR40 is illustrated in Figure 11C. The side chain of LI01 mutant formed π–H and H-bond interaction with GPR40, which fixed LI01 mutant in GPR40. However, the carboxyl group of LI01 did not form ionic or hydrogen bond interactions with ARG residues as expected. The AG02 and DE20 mutants successfully formed ionic and hydrogen bonds by increasing the GBVI score from −10.2063 to −8.8651 and −8.8189, respectively. The absence of ionic or hydrogen bonds in the GPR40–LI01 mutant complex results in a lower GBVI score at −8.4173 (Table 2). Standard energy minimization of the TAK-875 crystal structure was performed on three mutants to compare their performance. When energy minimization was done, the GBVI scores of the AG02 mutant and DE20 mutant were attained to −10.043 and −10.404, respectively. Both mutants showed expected performance. The carboxyl end of the LI01 mutant successfully contacted the ARG residues and formed ionic and hydrogen bonds, but the pockets might not be gathered to this extent, and it was difficult for the LI01 mutant to form ionic and hydrogen bonds. One solution worth considering was to simplify the side chain of the peptide outside the pocket and further extend the fatty chain where the carboxyl group was located. Finally, 4-15, 5-1, 4-4, DE20 mutant, and AG02 mutant are considered as potential active peptides that are analyzed in MD simulations (Supplementary Figure 7).

### Molecular Dynamics Simulations of Five Peptides and Control

A 10-ns MD simulation was run on five peptides and the control. The structure at 10 ns shows that 5-1 and 4-4 are separated from the binding site (Supplementary Figures 8D,E), which indicated that the binding stability of the two under simulated physiological conditions was insufficient. The GBVI of the structure at 10 ns was calculated. The 5-1 and 4-4 that were separated from the binding site showed poor GBVI. The GBVI values of 4-15, AG02 mutant, DE20 mutant, and the control that remained at the binding site are −9.196, −8.564, −7.335, and −8.542, respectively (Supplementary Figures 8A–C,F and Table 3). The GBVI of all peptides increased, but the increase of the GBVI of the peptides remaining in the binding site was smaller, among which 4-15 showed the best GBVI maintenance ability. The two peptides leaving the binding site were excluded due to insufficient stability, and the remaining three peptides and the control were further analyzed.

**TABLE 3:** Comparison of peptide and control at the beginning and end of molecular dynamics simulation.

**Name**	**GBVI at the beginning of MD**	**GBVI at the end of MD**	**Depart from GPR40**
4-15	−10.206	−9.196	No
5-1	−9.620	−5.913	Yes
4-4	−9.579	−5.765	Yes
AG02	−10.043	−8.564	No
DE20	−10.404	−7.335	No
Control	−9.749	−8.542	No

*GBVI, generalized Born/volume integral.*

**FIGURE 12:**
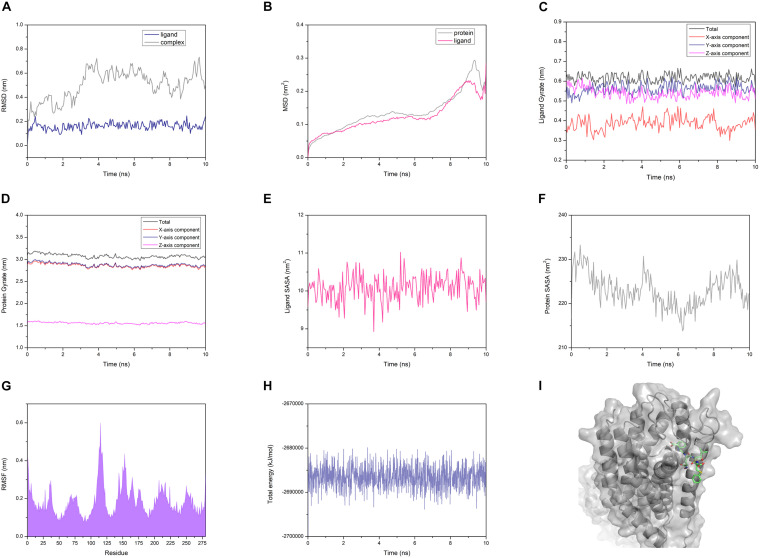
**(A)** Root mean square deviation (RMSD) of the AG02 mutant and the complex. **(B)** Mean square displacement (MSD) of the AG02 mutant and GPR40 in the complex. **(C)** Gyrate of the AG02 mutant in the complex. **(D)** Gyrate of the GPR40 in the AG02 mutant complex. **(E)** Solvent-accessible surface area (SASA) of the AG02 mutant in the complex. **(F)** SASA of the GPR40 in the AG02 mutant complex. **(G)** Root mean square fluctuation (RMSF) of the GPR40 in the AG02 mutant complex. **(H)** The total energy of the AG02 mutant complex. **(I)** The AG02 mutant complex structures at 10 ns.

The AG02 is selected as a representative example for MD analysis (Figure 12). RMSD measured the average positional change of all atoms between two structures. Based on the initial structure, the RMSD of all ligands and complexes was calculated. The RMSD calculated based on the protein and the complex was almost the same, so only the RMSD data of the complex were retained. AG02 mutant, DE20 mutant, and the control as ligands maintain relatively stable RMSD values throughout the simulation process (Supplementary Figures 9B–D). As a ligand, 4-15 showed a large increase in RMSD at about 3 ns then decreased and stabilized after 6 ns. When the RMSD of the 4-15 ligand is stable, the value is the largest among the four ligands, which indicates that the largest posture change occurred during the simulation (Supplementary Figure 9A). However, 4-15 retained the best GBVI at the end of the simulation, which might indicate that it was adjusted to a stable and high-affinity conformation. After the RMSD of the four simulated complexes was compared, the control complex and the DE20 complex show a stable trend at the end of the simulation (Supplementary Figure 9). The RMSD fluctuation of the AG02 complex in the later stage was larger than the former two. The 4-15 complex with the best affinity had a RMSD mutation in the late simulation stage, which might indicate that the conformation of the 4-15 complex will continue to change. MSD, the mean of the squared displacements of all atoms, was used to analyze the changes in the positions of proteins and ligands. The MSD values of the proteins and ligands in the control complex are the most stable (Supplementary Figure 10). The MSD changes of the proteins and ligands of the AG02 complex and the DE20 complex have similar trends, while the MSDs of the two components of the 4-15 complex are quite different (Supplementary Figure 10A). The protein and ligand of the 4-15 complex might have greater relative displacements resulting in low stability. Gyrate reflects the volume and shape of the molecule, and an increase in gyrate indicates that the system have expanded. The gyrate of DE20 mutant and the control is more stable than the other two molecules (Supplementary Figure 11). The *X*-axis component of the control was lower than the *Y*- and *Z*-axes, and its gyrate mainly derived from the plane where the *Y*- and *Z*-axes were located, which was related to the linear structure of the control. The gyrate of 4-15 entered a period of stability when the simulation was approaching the late stage but changed at the end of the simulation. The gyrate of the AG02 mutant was maintained in a relatively large interval during the entire simulation process, and the changes in the three-axis components of the AG02 mutant were also maintained in a range (Supplementary Figure 11B). The gyrates of the protein components of the three complexes other than 4-15 show a stable trend throughout the simulation process (Supplementary Figure 12). The protein component of the 4-15 complex expanded in volume at about 7 ns and then shrank. This might be due to the process of 4-15 adjusting its conformation to a higher affinity.

Similarly, the SASA of 4-15 is different from that of the other three ligands in the whole simulation process (Supplementary Figure 13). After 6 ns, the SASA of 4-15 dropped sharply, indicating that its conformation became tighter. At the end of the simulation, the 4-15 SASA returned to the level before 6 ns. 4-15 had undergone a large degree of conformation adjustment between 6 and 10 ns and maintained a high affinity at the end of the simulation. The SASA calculated based on the protein composition shows that 4-15 had the most stable trend (Supplementary Figure 14). This might be due to AG02, DE20, and the control mainly relying on the combination of salt bridge and GPR40, while 4-15 mainly relies on van der Waals interaction. 4-15 had the most stable hydrophobic interaction. RMSF reflected the average position change amplitude of the residue atoms in 10 ns. The protein residues of the four complexes all have similar RMSF distributions (Supplementary Figure 15). The residues numbered 75-85 and 130-140 showed lower RMSF. This region was where the binding cleft was located, which indicated that the residues at this location had higher stability due to binding with the ligand. The residues numbered 183 and 258 are arginine acids that form a salt bridge, and the RMSF recessed appears adjacent to these two residues (Supplementary Figure 15). The degree of depression of 4-15 near residues 183 and 258 was lower than that of the other three ligands, which might be due to 4-15 relying on van der Waals interaction instead of forming a salt bridge with arginine. The 4-15 complex has a total energy similar to that of the control, and the other two peptide complexes have a total energy lower than that of the control (Supplementary Figure 16). In addition, the total energy fluctuation of the peptide complex was similar to that of the control. 4-15, AG02 mutant, and DE20 mutant showed good performance similar to TAK-875 in MD simulations, and the three peptides were considered as potential GPR-40 agonists.

## Discussion

In this study, CNN can effectively extract local features by local receptive fields. CNN reduces complexity by sharing weights. It is a topological structure that uses spatial relationships to reduce the number of parameters, thereby improving the training efficiency of feedforward neural networks. Therefore, CNN can successfully realize the deep structure and effectively control the occurrence of overfitting. To be able to perform the CNN, the 153 molecular features were converted to 1D sequences. We attempted to maximize the use of feature information through the deep structure of 1D CNN. However, the performance of CNN on the GPR40 agonist data set was less efficient than that of the three-layer DNN with a fully connected structure. A total of 153 features are independent according to their specific meanings (Supplementary Table 2). Local features were very important for sound signal, vibration signal, and text processing. Compared with global information extraction, local detail extraction could be used for model training more efficiently on signals like sound. But for the quantitative physical and chemical properties and composition information of molecules, the significance of the transformation and extraction of multiple features by the convolutional layer was not as great as the former. The molecular feature sequence lacked overall spatial information because 153 features had no spatial correlation. This would cause the local receptive field to extract the meaningless characteristics of neighboring spatial information. Although it had a deeper network structure, the partial failure of the local receptive field and the simplification of the connection structure might be the reason for the performance degradation of CNN. CNN was an excellent feature extractor, but the processing and optimization of 2D features with high independence and low noise and no adjacent spatial information would not get positive feedback. The feedback of PCA feature dimensionality reduction and LASSO feature selection on DNN also supported this view, and both methods led to variable degrees of performance degradation. The MSE shock of CNN at the end of the training was stronger than that of DNN; this is possibly because the data set is too small and the batch size of CNN is smaller than that of DNN.

For small data sets, the performance of MOE 2D molecular features was better than that of Morgan fingerprint features. The information content of a single MOE 2D descriptor was more complex than Morgan fingerprints, and the highly integrated features reduced the difficulty of convergence for the model. The 1,024-bit Morgan fingerprint was more suitable for large data sets. Although the data set had undergone the necessary preprocessing, the noise of EC50 labels measured by different laboratories could not be ignored. The instruments, methods, and materials used by the researcher during the label determination process would all affect the label. The error of the label and the size of the data set were the main reasons that restrict the accuracy of the DNN. How to obtain high-quality data sets was still an important issue in the development of AI.

Compared with that by non-processing, the number of new PCA features obtained by orthogonal projection is significantly reduced. Still, the apparent decline of DNN performance indicated that important information was lost in the PCA processing. The LASSO regression estimator retained 83 features with nonzero coefficients, and DNN showed a slight loss of accuracy. The model was not optimized for accuracy after the preprocessing of the features was completed, which might be due to less noise in the features.

For all the scoring functions, there was a noticeable feature from the result that the order of the value from each scoring function is Max > Dis/Ave > Min, which was consistent with their definition. The “Must match” function was so strict that none of the entities in the testing set match the fingerprint model. In some distance, a high standard of the scoring function is not always appropriate for the fingerprint method. For finding the novel compounds as GPR40 agonists, a high standard might lead one to miss potent candidates in screening. In the “Min” function, the scores were low and close to each other, which were not identical to the “Max” function. The model that could distinguish active entities from inactive entities is our goal. Therefore, the “Max” function was chosen to be the scoring function in this study. For the fingerprint type, the high absolute value of k is a key characteristic to evaluate the quality of each fingerprint type, and the GpiDAPH3 showed gorgeous output in this study.

The inputs of DNN are some quantitative molecular features, and different weights are assigned to the features after training. Compared with DNN, MFS recognizes the common features of data sets. Most molecules in the data set have similar main body skeletons, so the active molecules exported by MFS have skeleton similarity. DNN can have different tendencies in the main body skeleton and detailed features through weight distribution. Here, DNN tended to feature details, but access to the GPR40 binding site required certain linearity of the molecule. Therefore, the two models were combined to limit the linear and affinity groups of the peptides. In addition, DNN combined with MFS to predict peptides reduced the influence of label noise in the data set to a certain extent. For large data sets, it may be difficult for the MFS to extract a sufficient number of common features, while the DNN will have better performance due to its complex network structure.

The docking results show that the peptides are difficult to form an ion–hydrogen bond interaction similar to that of TAK-875, which might be due to the steric hindrance given by the peptide backbone and the peptide side chain. In contrast, the molecular structure of TAK-875 was roughly linear with almost no branches. The linear structure helped TAK-875 penetrate deep into GPR40 and formed ionic and hydrogen bonds with ARG183 and ARG258. As little steric hindrance as possible was necessary to pass through the narrow binding cleft. The data sets used for DNN and MFS contained a large number of TAK-875 analogs. DNN and MFS extracted high-frequency features, such as benzene ring, heterocyclic ring, carboxyl group, halogen, and sulfur element. Linear peptides contained at least one carboxyl group, which was the advantage of peptides to form ionic or hydrogen bonds. The main chain of the peptide was composed of repeating units of carbon-amide bonds, which meant that the groups preferred by the model could only exist in the side chain. The preference groups that exist only in the side chain led to the fact that the peptides selected by DNN and MFS were simple linear structures and much more complicated than TAK-875. The complex side-chain structure of peptides causes it proneness to molecular–residue spatial conflicts when passing through the binding cleft, which was not conducive to the deepening of the carboxyl-terminal or side chain ionic groups of the peptide.

It is worth noting that the single-point mutation of the AA within the binding cleft is an effective strategy to form ionic bonds or hydrogen bonds. Moreover, a sufficiently long chain with a carboxyl terminus is necessary, which is essential for the peptide to reach ARG183 and ARG258. However, the long-chain carboxyl AAs of the LI01 mutant failed to reach the residues of ARG183 and ARG258. This is because the complex moiety outside the alpha helix of the LI01 mutant prevented the mutant from approaching GPR40. Interestingly, the mutant AAs of the AG02 mutant and the DE20 mutant did not form ionic bonds or hydrogen bonds with ARG residues as expected. Instead, the mutant binds to GPR40 in another position, and the mutant’s G28 passes through the alpha helix to form ionic and hydrogen bonds (Figures 11A,B).

The five peptides after flexible docking were all inserted into the binding cleft by one residue, and the remaining peptide residues on the outer surface had different effects on the affinity of peptides. The two main interactions of 4-15 were formed by CM25 and G28 on the outer surface. The affinity of 5-1 was mainly derived from two interactions: the π–H interaction formed by the G35 residue on the outer surface. The peptide residues on the outer surface of 4-4 contributed little to the affinity; and P107 and P131, respectively, formed only weak hydrogen bonds and π–H interactions. Most of the affinity was derived from the salt bridge formed by the G28 residue and the GPR40 residue entering the binding cleft for the two candidate mutants. The binding modes of peptides can be divided into two types: the binding mode is dominated by external surface residues; the other is the mode where a single residue penetrates the binding cleft. The mode of a single residue entering the binding pocket profoundly depends on the strong salt bridge. The mode dominated by residues on the outer surface is more dependent on van der Waals interactions. The two models may have different specificities and affinities, which can provide references for advanced peptide design.

4-4 and 5-1 broke away from the binding site in MD; even they obtain low GBVI upon the flexible docking. The GBVI of other ligands increases to varying degrees in MD (Table 3). This might be caused by conditions similar to those of the environment in the human body used by MD. The temperature of 310K increased the instability of the system. MD showed that the stability of peptides that relied on van der Waals interaction was lower than that of peptides that relied on salt bridges. Peptides that relied on salt bridges needed to enter the binding site as deep as possible to contact the arginine residues, which might be the main reason for their high stability. The peptides of the two binding modes (4-15 and mutants) showed different characteristics in many aspects of the MD analysis results. Although peptides that relied on salt bridges showed higher stability, peptides that relied on van der Waals interactions could achieve higher affinity by complex interactions.

## Conclusion

In summary, we developed a novel protocol to rationally design peptides and to identify a peptide database that may show potency for the activation of GPR40. Machine learning combined with MFS could efficiently distinguish between active and inactive molecules. The comparison results of the two machine learning methods showed that the DNN had better performance than the 1D CNN on the peptide activity prediction task based on small data sets. We found that the MFS model focused on the main skeleton of the chemical structure, and the DNN was able to pay attention to more structural details, so the combination of the two strategies could comprehensively scan the chemical structures. Besides, the SDM approach successfully optimized the interaction between the lead peptides and GPR40 to make the peptide more comparable with the active compound. Then, flexible docking, a credible method to evaluate the affinity, was performed to confirm the candidates; and five peptides were finally selected for MD simulations. Three peptides showing good stability in MD simulations were selected as promising leads against T2DM. The strategy concept described here can efficiently discover new active peptides and require only a small amount of computing resources, cost-effective for scale-up assessments in peptide drug development.

## Data Availability Statement

The original contributions presented in the study are included in the article/Supplementary Material, further inquiries can be directed to the corresponding authors.

## Author Contributions

JW, XC, and ZC conceived the idea, designed the study, and wrote the manuscript. JW and ZW directed the project, provided technical support, and corrections to the manuscript. XC and ZC performed all the experiments and analyzed the data. XC and ZC revised the manuscript according to the comments of JW and other co-authors. All authors contributed to the article and approved the submitted version.

## Conflict of Interest

The authors declare that the research was conducted in the absence of any commercial or financial relationships that could be construed as a potential conflict of interest.
