# Supplementary material for: De novo Design of G Protein-Coupled Receptor 40 Peptide Agonists for Type 2 Diabetes Mellitus Based on Artificial Intelligence and Site-Directed Mutagenesis
Source: Front Bioeng Biotechnol. 2021 Jun 14;9:694100. doi: 10.3389/fbioe.2021.694100 (PMC8236607; doi:10.3389/fbioe.2021.694100)
Supplement: Supplementary file 1 [file Table_1.DOCX]

**De Novo Design of GPR40 peptide agonists for T2DM based on Artificial Intelligence and Site-Directed Mutagenesis**

Xu Chen^1,2†^, Zhidong Chen^1,2†^, Daiyun Xu^2^, Yonghui Lyu^2^, Yongxiao Li^2^, Shengbin Li^2^, Junqing Wang^2^* Zhe Wang^1^*

^1^Department of Pathology, The Eighth Affiliated Hospital, Sun Yat-sen University, Shenzhen, 518033, China

^2^School of Pharmaceutical Sciences (Shenzhen), Sun Yat-sen University, Shenzhen, 518100, China

*** Correspondence:**Junqing Wang ([wangjunqing@mail.sysu.edu.cn](mailto:wangjunqing@mail.sysu.edu.cn))

Zhe Wang (Wangzh379@mail.sysu.edu.cn)

^†^These authors have contributed equally to this work

**Keywords:** T2DM; GPR40; Artificial Intelligence; Oligopeptides; Molecular Fingerprint; Site-Directed Mutagenesis

Content

[Table S1. 528 molecules in the data set. 3](#_Toc70783112)

[Table S2. Description of 153 MOE two-dimensional descriptors. 21](#_Toc70783113)

[Table S3. All categories of unnatural amino acids. 26](#_Toc70783114)

[Table S4. Non-zero coefficient features after Lasso feature selection. 27](#_Toc70783115)

[Table S5. 41 amino acids with a carboxyl chain. 29](#_Toc70783116)

[Figure S1. Loss function curve of CNN. 31](#_Toc70783117)

[Figure S2. Loss function curve of DNN. 32](#_Toc70783118)

[Figure S3. Loss function curve of DNN trained based on Morgan fingerprint. 33](#_Toc70783119)

[Figure S4. The GpiDAPH fingerprint model uses different score functions to evaluate the similarity score of the test set. (A) GpiDAPH_Ave, (B) GpiDAPH_Dis, (C) GpiDAPH_Max, (D) GpiDAPH_Min, (E) GpiDAPH_Must. Index 1-50 is active molecule and Index 51-100 is inactive molecule. 34](#_Toc70783120)

[Figure S5. Seven fingerprint models use the maximum scoring function to evaluate the similarity score of the test set. (A) Bit_MACCS_Max, (B) GpiDAPH_Max, (C) MACCS_Max, (D) TAD_Max, (E) TAT_Max , (F) TGD_Max, (G) TGT_Max. Index 1-50 is active molecule and Index 51-100 is inactive molecule. 36](#_Toc70783121)

[Figure S6. The similarity scores of the redefined test set on the (A) Bit_MACCS, (B) GpiDAPH, and (C) MACCS models. Index 1-50 is active molecule and Index 51-100 is inactive molecule. 37](#_Toc70783122)

[Figure S7. The 5 peptides were considered as potential active peptides that were analyzed in molecular dynamics simulations. (A) 4-15, (B) 5-1, (C) 4-4, (D) DE20 mutant, (E) AG02 mutant. 38](#_Toc70783123)

[Figure S8. The complex structures of five peptides and control at 10ns. (A) 4-15, (B) AG02 mutant, (C) DE20 mutant, (D) 4-4, (E) 5-1, (F) TAK-875. 39](#_Toc70783124)

[Figure S9. RMSD of the ligand and the complex. (A) 4-15, (B) AG02 mutant, (C) DE20 mutant, (D) TAK-875. 40](#_Toc70783125)

[Figure S10. MSD of the ligand and GPR40 in the complex. (A) 4-15, (B) AG02 mutant, (C) DE20 mutant, (D) TAK-875. 41](#_Toc70783126)

[Figure S11. Gyrate of the ligand in the complex. (A) 4-15, (B) AG02 mutant, (C) DE20 mutant, (D) TAK-875. 42](#_Toc70783127)

[Figure S12. Gyrate of the GPR40 in the complex. (A) 4-15, (B) AG02 mutant, (C) DE20 mutant, (D) TAK-875. 43](#_Toc70783128)

[Figure S13. SASA of the ligand in the complex. (A) 4-15, (B) AG02 mutant, (C) DE20 mutant, (D) TAK-875. 44](#_Toc70783129)

[Figure S14. SASA of the GPR40 in the complex. (A) 4-15, (B) AG02 mutant, (C) DE20 mutant, (D) TAK-875. 45](#_Toc70783130)

[Figure S15. RMSF of the GPR40 in the complex. (A) 4-15, (B) AG02 mutant, (C) DE20 mutant, (D) TAK-875. 46](#_Toc70783131)

[Figure S16. Total energy of the complex. (A) 4-15, (B) AG02 mutant, (C) DE20 mutant, (D) TAK-875. 47](#_Toc70783132)

## Table S1. 528 molecules in the data set.

| **ChEMBL ID** | **pEC_50_** | **Smiles** |
| --- | --- | --- |
| CHEMBL3944424 | 9.523 | FC(F)(F)c1c(C(C)N2CC(COc3cc([C@@H]([C@@H](C(=O)O)C)C4CC4)ccc3)C2)cc(C(F)(F)F)cc1 |
| CHEMBL3922381 | 9.097 | FC(F)(F)CN(Cc1cc(COc2ccc([C@@H](C#CC)CC(=O)O)cc2)ccc1)Cc1cscc1 |
| CHEMBL3287571 | 8.886 | Clc1cc(C)c(-c2cc(COc3ccc([C@H](CC(=O)O)c4n(C)nnn4)cc3)ccc2)cc1 |
| CHEMBL3904378 | 8.854 | O=C(O)C[C@H](C#CC)c1ccc(OCc2cc(CN(CC(C)C)Cc3cscc3)ccc2)cc1 |
| CHEMBL3966739 | 8.721 | O=C(O)C[C@H](C#CC)c1ccc(OCc2cc(CN(Cc3cscc3)C3CC3)ccc2)cc1 |
| CHEMBL3896509 | 8.721 | O=C(O)C[C@H](C#CC)c1ccc(OCc2cc(CN(Cc3cscc3)Cc3ccncc3)ccc2)cc1 |
| CHEMBL3923431 | 8.699 | O=C(O)C[C@H](C#CC)c1ccc(OCc2cc(CN(Cc3cscc3)C3CCCCC3)ccc2)cc1 |
| CHEMBL3889768 | 8.678 | O=C(O)C[C@H](C#CC)c1ccc(OCc2cc(CN(Cc3cscc3)Cc3cscc3)ccc2)cc1 |
| CHEMBL1829173 | 8.658 | FC(F)(F)c1ccc(-c2cc(COc3ccc([C@@H](C#CC)CC(=O)O)cc3)ccc2)cc1 |
| CHEMBL3287574 | 8.444 | FC(F)(F)c1ccc(-c2sc(COc3ccc([C@H](CC(=O)O)c4nocc4)cc3)c(C)n2)cc1 |
| CHEMBL3924534 | 8.357 | O=C(O)C[C@H](C#CC)c1ccc(OCc2cc(CN(Cc3cscc3)Cc3ccccc3)ccc2)cc1 |
| CHEMBL2048618 | 8.347 | Fc1c(CCC(=O)O)ccc(OCc2cc(-c3c(C)cc(OCCOCC)cc3C)ccc2)c1 |
| CHEMBL3951157 | 8.337 | O=C(O)C[C@H](C#CC)c1ccc(OCc2cc(CN(Cc3cscc3)C3CCCC3)ccc2)cc1 |
| CHEMBL4460582 | 8.319 | Fc1c(OCC(=O)O)ccc(NCc2cc(-c3c(C)cccc3)ccc2)c1 |
| CHEMBL3918387 | 8.155 | S(=O)(=O)(N(Cc1cc(COc2ccc([C@@H](C#CC)CC(=O)O)cc2)ccc1)Cc1cscc1)CC |
| CHEMBL3604048 | 8.143 | FC(F)(F)c1ccc(COc2ccc(C(OCC)CC(=O)O)cc2)cc1 |
| CHEMBL3604058 | 8.125 | FC(F)(F)c1c(F)cc(COc2ccc([C@@H](OCC)CC(=O)O)cc2)cc1 |
| CHEMBL3914686 | 8.114 | O=C(O)C[C@H](C#CC)c1ccc(OCc2cc(CN(Cc3cscc3)C3CCOCC3)ccc2)cc1 |
| CHEMBL3914418 | 8.102 | O=C(O)C[C@H](C#CC)c1ccc(OCc2cc(CN(Cc3cscc3)C3CCSCC3)ccc2)cc1 |
| CHEMBL4228985 | 8.102 | Clc1c(-c2c(C)onc2C)cc(COc2cc3OC[C@@H](CC(=O)O)c3cc2)cc1 |
| CHEMBL4218596 | 8.097 | O(CCC(O)(C)C)c1cc(C)c(-c2c3c(sc2)ccc(COc2ccc([C@@H](C#CC)Cc4[nH]nnn4)cc2)c3)cc1 |
| CHEMBL3913383 | 8.066 | O=C(O)C[C@H](C#CC)c1ccc(OCc2cc(CN(CCOC)Cc3cscc3)ccc2)cc1 |
| CHEMBL3975239 | 8.066 | O=C(O)C[C@H](C#CC)c1ccc(OCc2cc(CN(C(C)C)Cc3cscc3)ccc2)cc1 |
| CHEMBL3287576 | 8.046 | FC(F)(F)c1ccc(-c2sc(COc3ccc([C@H](CC(=O)O)c4ocnc4)cc3)c(C)n2)cc1 |
| CHEMBL4206445 | 8.046 | O(Cc1cc2c(-c3c(C)cccc3)csc2cc1)c1ccc([C@@H](C#CC)Cc2[nH]nnn2)cc1 |
| CHEMBL4202907 | 8.046 | O(CCOc1cc(C)c(-c2c3c(sc2)ccc(COc2ccc([C@@H](C#CC)Cc4[nH]nnn4)cc2)c3)cc1)C |
| CHEMBL3799898 | 8.032 | Clc1c(COc2cc(F)c(CCC(=O)O)cc2)cc(-c2c(C)onc2C)cc1 |
| CHEMBL4164625 | 8.027 | Brc1c(-c2[nH]c3c(c2)cc(CCC(=O)O)cc3)cccc1 |
| CHEMBL3604065 | 8.004 | FC(F)(F)Oc1c2c([C@H](Oc3ccc([C@@H](OCC)CC(=O)O)cc3)CC2)ccc1 |
| CHEMBL2022256 | 8.000 | O=C(O)CC1c2c(OC1)cc(OCc1cc(-c3c(C)cccc3C)c(OCc3ccccc3)cc1)cc2 |
| CHEMBL3893141 | 8.000 | S(=O)(=O)(N(Cc1cc(COc2ccc([C@@H](C#CC)CC(=O)O)cc2)ccc1)Cc1cscc1)C1CC1 |
| CHEMBL3954235 | 8.000 | Clc1cc([C@H]2CN([C@@H](CC(=O)O)C2)c2cc(Cc3c(C)cc(-c4c(F)ccc(OC)c4)cc3)ccc2)ccc1 |
| CHEMBL4203795 | 8.000 | Fc1c2scc(-c3c(C)cc(OC)cc3)c2cc(COc2ccc([C@@H](C#CC)Cc3[nH]nnn3)cc2)c1 |
| CHEMBL4281133 | 8.000 | Clc1cc(OCCCOc2ccc([C@@H](C#CC)CC(=O)O)cc2)ccc1 |
| CHEMBL3798044 | 7.955 | Fc1c(CCC(=O)O)ccc(OCc2c(F)ccc(-c3c(C)onc3C)c2)c1 |
| CHEMBL2381289 | 7.947 | FC(F)(F)c1cc([C@H]2Oc3c(OC2)cc2[C@H](CC(=O)O)COc2c3)ccc1 |
| CHEMBL3930086 | 7.936 | O=C([O-])C[C@H](C#CC)c1ccc(OCc2cc(CN(C(C)C)Cc3cscc3)ccc2)cc1.O=C([O-])C[C@H](C#CC)c1ccc(OCc2cc(CN(C(C)C)Cc3cscc3)ccc2)cc1.[Ca+2] |
| CHEMBL4575598 | 7.936 | Fc1c(OCC(=O)O)ccc(NCc2cc(-c3c(C)cc(OCC)cc3)ccc2)c1 |
| CHEMBL2048614 | 7.921 | O=C(O)CCc1ccc(OCc2cc(-c3c(C)cc(OC)cc3C)ccc2)cc1 |
| CHEMBL3604047 | 7.921 | Clc1ccc(COc2ccc(C(OCC)CC(=O)O)cc2)cc1 |
| CHEMBL3604037 | 7.921 | Clc1c(Cl)ccc(COc2cc(F)c(C(OCC)CC(=O)O)cc2)c1 |
| CHEMBL3604061 | 7.921 | O=C(O)C[C@H](OCC)c1ccc(O[C@H]2c3c(c(CC)ccc3)CC2)cc1 |
| CHEMBL3604062 | 7.921 | O=C(O)C[C@H](OCC)c1ccc(O[C@H]2c3c(c(C(C)C)ccc3)CC2)cc1 |
| CHEMBL3800362 | 7.921 | Fc1c(CCC(=O)O)ccc(OCc2cc(C)cc(-c3c(C)onc3C)c2)c1 |
| CHEMBL3957744 | 7.921 | O=C(O)C[C@H](C#CC)c1ccc(OCc2cc(CN(CCO)Cc3cscc3)ccc2)cc1 |
| CHEMBL4206318 | 7.921 | S(=O)(=O)(CCCOc1cc(C)c(-c2c3c(sc2)ccc(COc2ccc([C@@H](C#CC)Cc4[nH]nnn4)cc2)c3)cc1)C |
| CHEMBL2048617 | 7.886 | O=C(O)CCc1ccc(OCc2cc(-c3c(C)cc(OCCOCC)cc3C)ccc2)cc1 |
| CHEMBL3603843 | 7.886 | FC(F)(F)c1cc2c([C@H](Oc3ccc([C@@H](OCC)CC(=O)O)cc3)CC2)cc1 |
| CHEMBL3604063 | 7.886 | O=C(O)C[C@H](OCC)c1ccc(O[C@H]2c3c(c(C4CC4)ccc3)CC2)cc1 |
| CHEMBL3604052 | 7.886 | Fc1c2c(c(COc3ccc(C(OCC)CC(=O)O)cc3)cc1)cccc2 |
| CHEMBL3604057 | 7.886 | FC(F)(F)c1cc(F)c(COc2ccc([C@@H](OCC)CC(=O)O)cc2)cc1 |
| CHEMBL3931755 | 7.886 | Fc1c(COc2ncc3[C@@H]4C(C(=O)O)[C@@H]4Cc3c2)cc(-c2c(C)cc(OCCC(O)C)cc2C)cc1 |
| CHEMBL4209834 | 7.886 | FC(F)(F)c1c(-c2c3c(sc2)ccc(COc2ccc([C@@H](C#CC)Cc4[nH]nnn4)cc2)c3)cccc1 |
| CHEMBL4207592 | 7.886 | FC(F)(F)c1c(-c2c(C)cc(OCCOC)cc2)c2c(s1)ccc(COc1ccc([C@@H](C#CC)Cc3[nH]nnn3)cc1)c2 |
| CHEMBL2381282 | 7.870 | Brc1cc([C@H]2Oc3c(OC2)cc2[C@H](CC(=O)O)COc2c3)ccc1 |
| CHEMBL2048616 | 7.854 | O=C(O)CCc1ccc(OCc2cc(-c3c(C)cc(OCc4ccccc4)cc3C)ccc2)cc1 |
| CHEMBL2048624 | 7.824 | Fc1c(CCC(=O)O)ccc(OCc2cc(-c3c(C)cc(OCC4(O)CCS(=O)(=O)CC4)cc3C)ccc2)c1 |
| CHEMBL2048615 | 7.824 | O=C(O)CCc1ccc(OCc2cc(-c3c(C)cc(OCC4CC4)cc3C)ccc2)cc1 |
| CHEMBL4213937 | 7.824 | Fc1c(-c2c(C)cccc2)c2c(s1)ccc(COc1ccc([C@@H](C#CC)Cc3[nH]nnn3)cc1)c2 |
| CHEMBL4218348 | 7.824 | FC(F)(F)c1c(-c2c(C)cccc2)c2c(s1)ccc(COc1ccc([C@@H](C#CC)Cc3[nH]nnn3)cc1)c2 |
| CHEMBL4202452 | 7.810 | S(=O)(=O)(CCCOc1cc(C)c(c(C)c1)-c1cc(COc2ccc(CCC(=O)O)cc2)ccc1)C |
| CHEMBL4593950 | 7.804 | FC(F)(F)c1c(-c2cc(CNc3cc(F)c(OCC(=O)O)cc3)ccc2)cccc1 |
| CHEMBL3799274 | 7.799 | Clc1c(-c2c(C)onc2C)cc(COc2cc(F)c(CCC(=O)O)cc2)cc1 |
| CHEMBL2381287 | 7.796 | Clc1c(Cl)ccc([C@H]2Oc3c(OC2)cc2[C@H](CC(=O)O)COc2c3)c1 |
| CHEMBL4206968 | 7.796 | O(Cc1cc2c(c(C#N)sc2cc1)-c1c(C)cccc1)c1ccc([C@@H](C#CC)Cc2[nH]nnn2)cc1 |
| CHEMBL3895442 | 7.793 | O=C(O)C[C@H](C#CC)c1ccc(OCc2cc(CN(Cc3cscc3)CC3CC3)ccc2)cc1 |
| CHEMBL3933446 | 7.777 | O=C(O)C[C@H](C#CC)c1ccc(OCc2cc(CN(Cc3cscc3)C3CCS(=O)(=O)CC3)ccc2)cc1 |
| CHEMBL4217348 | 7.745 | Fc1c(-c2c(C)cc(OCCOC)cc2)c2c(s1)ccc(COc1ccc([C@@H](C#CC)Cc3[nH]nnn3)cc1)c2 |
| CHEMBL4228406 | 7.730 | Clc1c(-c2c(C)onc2C)cc(COc2cc3OCC(CC(=O)O)c3cc2)cc1 |
| CHEMBL3604041 | 7.721 | Clc1cc(COc2ccc(C(OCC)CC(=O)O)cc2)ccc1 |
| CHEMBL4202612 | 7.721 | Brc1c(-c2c(C)cccc2)c2c(s1)ccc(COc1ccc([C@@H](C#CC)Cc3[nH]nnn3)cc1)c2 |
| CHEMBL2381308 | 7.719 | O=C(O)C[C@H]1c2c(OC1)cc1O[C@@H](c3cc(-c4c(C)cccc4)ccc3)COc1c2 |
| CHEMBL4438440 | 7.714 | Clc1c(-c2cc(CNc3cc(F)c(OCC(=O)O)cc3)ccc2)cccc1 |
| CHEMBL2048613 | 7.699 | O=C(O)CCc1ccc(OCc2cc(-c3c(C)cccc3C)c(OC)cc2)cc1 |
| CHEMBL3604067 | 7.699 | FC(F)(F)c1c2c([C@H](Oc3ncc([C@@H](OCC)CC(=O)O)cc3)CC2)ccc1 |
| CHEMBL3785637 | 7.699 | Fc1c(CCC(=O)O)ccc(C#C/C(=C\C)/C)c1 |
| CHEMBL4208095 | 7.699 | O(Cc1cc2c(c(C)sc2cc1)-c1c(C)cccc1)c1ccc([C@@H](C#CC)Cc2[nH]nnn2)cc1 |
| CHEMBL4214289 | 7.699 | Clc1c(-c2c(C)cccc2)c2c(s1)ccc(COc1ccc([C@@H](C#CC)Cc3[nH]nnn3)cc1)c2 |
| CHEMBL2022252 | 7.678 | O=C(O)CC1c2c(OC1)cc(OCc1cc(-c3c(C)ccc4c3cccc4)ccc1)cc2 |
| CHEMBL3604066 | 7.678 | FC(F)(F)c1c2OC[C@@H](Oc3ccc([C@@H](OCC)CC(=O)O)cc3)c2ccc1 |
| CHEMBL4537190 | 7.648 | Clc1c(-c2cc(CNc3cc(F)c(OCC(=O)O)cc3)ccc2)ccc(C)c1 |
| CHEMBL3798575 | 7.640 | Fc1c(CCC(=O)O)ccc(OCc2cc(-c3c(C)onc3C)c(OC)cc2)c1 |
| CHEMBL2048619 | 7.638 | Fc1c(CCC(=O)O)ccc(OCc2cc(-c3c(C)cc(OC4CCOCC4)cc3C)ccc2)c1 |
| CHEMBL2048626 | 7.638 | Fc1c(CCC(=O)O)ccc(NCc2cc(-c3c(C)cc(OC4CCS(=O)(=O)CC4)cc3C)ccc2)c1 |
| CHEMBL2022244 | 7.638 | O=C(O)CC1c2c(cc(OCc3cc(-c4c(C)cccc4C)ccc3)cc2)CCC1 |
| CHEMBL3604044 | 7.638 | O=C(O)CC(OCC)c1ccc(OCc2cc(Oc3ccccc3)ccc2)cc1 |
| CHEMBL4204345 | 7.638 | Fc1c2scc(-c3c(C)cccc3)c2cc(COc2ccc([C@@H](C#CC)Cc3[nH]nnn3)cc2)c1 |
| CHEMBL2022255 | 7.620 | O=C(O)CC1c2c(OC1)cc(OCc1cc(-c3c(C)cccc3C)c(OC)cc1)cc2 |
| CHEMBL4570807 | 7.616 | Fc1c(OCC(=O)O)ccc(NCc2cc(-c3c(C)cc(OCC)cc3C)ccc2)c1 |
| CHEMBL3798341 | 7.614 | Fc1c(CCC(=O)O)ccc(OCc2c(OC)ccc(-c3c(C)onc3C)c2)c1 |
| CHEMBL2048622 | 7.602 | S(=O)(=O)(CCOc1cc(C)c(c(C)c1)-c1cc(COc2cc(F)c(CCC(=O)O)cc2)ccc1)CC |
| CHEMBL2048623 | 7.602 | Fc1c(CCC(=O)O)ccc(OCc2cc(-c3c(C)cc(OC4CCS(=O)(=O)CC4)cc3C)ccc2)c1 |
| CHEMBL4203837 | 7.602 | Clc1c2scc(-c3c(C)cc(OC)cc3)c2cc(COc2ccc([C@@H](C#CC)Cc3[nH]nnn3)cc2)c1 |
| CHEMBL3800232 | 7.599 | Fc1c(CCC(=O)O)ccc(OCc2cc(-c3c(C)onc3C)c(F)cc2)c1 |
| CHEMBL3798274 | 7.587 | Fc1c(COc2cc(F)c(CCC(=O)O)cc2)cccc1-c1c(C)onc1C |
| CHEMBL3604033 | 7.569 | Clc1c(Cl)ccc(CNc2ccc(C(OCC)CC(=O)O)cc2)c1 |
| CHEMBL4228620 | 7.561 | O=C(O)CC1c2c(OC1)cc(OCc1cc(-c3c(C)onc3C)ccc1)cc2 |
| CHEMBL2022576 | 7.553 | O=C(O)CC1c2c(OC1)cc(OCc1cc(-c3c(C)cc(OCc4ccccc4)cc3C)ccc1)cc2 |
| CHEMBL2022577 | 7.553 | O=C(O)CC1c2c(OC1)cc(OCc1cc(-c3c(C)cc(OCCOCC)cc3C)ccc1)cc2 |
| CHEMBL2022243 | 7.553 | O=C(O)CC1c2c(cc(OCc3cc(-c4c(C)cccc4C)ccc3)cc2)CC1 |
| CHEMBL4218709 | 7.553 | Ic1c(-c2c(C)cccc2)c2c(s1)ccc(COc1ccc([C@@H](C#CC)Cc3[nH]nnn3)cc1)c2 |
| CHEMBL4218946 | 7.540 | Clc1c(-c2cc(COc3ccc(S(=O)(=O)CC(=O)O)cc3)ccc2)cccc1 |
| CHEMBL2022257 | 7.538 | O=C(O)CC1c2c(OC1)cc(OCc1cc(-c3c(C)cc(C)cc3)ccc1)cc2 |
| CHEMBL2048627 | 7.538 | Fc1c(CCC(=O)O)ccc(NCc2cc(-c3c(C)cc(OCC4(O)CCS(=O)(=O)CC4)cc3C)ccc2)c1 |
| CHEMBL4206640 | 7.538 | O(CC(O)CO)c1cc(C)c(-c2c3c(sc2)ccc(COc2ccc([C@@H](C#CC)Cc4[nH]nnn4)cc2)c3)cc1 |
| CHEMBL3921143 | 7.529 | O=C(O)C[C@H](C#CC)c1ccc(OCc2cc(CN(C(=O)C)Cc3cscc3)ccc2)cc1 |
| CHEMBL3604040 | 7.523 | Fc1cc(COc2ccc(C(OCC)CC(=O)O)cc2)ccc1 |
| CHEMBL4209136 | 7.523 | O=S1(=O)CC(COc2cc(C)c(-c3c4c(sc3)ccc(COc3ccc([C@@H](C#CC)Cc5[nH]nnn5)cc3)c4)cc2)(C)C1 |
| CHEMBL2381306 | 7.521 | O=C(O)C[C@H]1c2c(OC1)cc1O[C@@H](c3cc(-c4c(C)cccc4C)ccc3)COc1c2 |
| CHEMBL2381290 | 7.517 | FC(F)(F)c1cc([C@@H]2Oc3c(OC2)cc2[C@H](CC(=O)O)COc2c3)ccc1 |
| CHEMBL3427705 | 7.509 | O=C(O)C[C@H](OCC)c1ccc(O[C@H]2c3c(cccc3)CC2)cc1 |
| CHEMBL2048621 | 7.495 | Fc1c(CCC(=O)O)ccc(OCc2cc(-c3c(C)cc(OCCCN4C(=O)CCC4)cc3C)ccc2)c1 |
| CHEMBL3799946 | 7.488 | Fc1c(CCC(=O)O)ccc(OCc2cc(-c3c(C)onc3C)ccc2)c1 |
| CHEMBL2022258 | 7.481 | O=C(O)CC1c2c(OC1)cc(OCc1cc(-c3c(C)cc(C)cc3C)ccc1)cc2 |
| CHEMBL3287570 | 7.481 | Clc1cc(C)c(-c2cc(COc3ccc([C@H](CC(=O)O)c4n(C)ncn4)cc3)ccc2)cc1 |
| CHEMBL3799639 | 7.479 | Fc1c(CCC(=O)O)ccc(OCc2cc(F)cc(-c3c(C)onc3C)c2)c1 |
| CHEMBL2381285 | 7.466 | Clc1cc([C@H]2Oc3c(OC2)cc2[C@H](CC(=O)O)COc2c3)ccc1 |
| CHEMBL3780042 | 7.460 | O=C(O)CC1c2c(OC1)cc(OCc1cc(-n3c(C)ccc3C)c(OC)cc1)cc2 |
| CHEMBL3604049 | 7.456 | O=C(O)CC(OCC)c1ccc(OCC2CCCCC2)cc1 |
| CHEMBL2315532 | 7.450 | O=C(O)[C@@H]1[C@@H](c2ccc(C#Cc3c(CC#N)cccc3)cc2)C1 |
| CHEMBL2381312 | 7.445 | Fc1cc(C)c(-c2cc(C3Oc4c(OC3)cc3[C@H](CC(=O)O)COc3c4)ccc2)cc1 |
| CHEMBL3287568 | 7.444 | Clc1cc(C)c(-c2cc(COc3ccc([C@@H](C#CC)CC(=O)O)cc3)ccc2)cc1 |
| CHEMBL3604068 | 7.444 | FC(F)(F)c1c2OC[C@@H](Oc3ncc([C@@H](OCC)CC(=O)O)cc3)c2ccc1 |
| CHEMBL4534840 | 7.442 | Fc1c(OCC(=O)O)ccc(NCc2cc(-c3c(C)cc(OCCO)cc3)ccc2)c1 |
| CHEMBL4209491 | 7.440 | Clc1c(-c2cc(COc3ccc(S(=O)(=O)CC(=O)O)cc3)ccc2)ccc(C)c1 |
| CHEMBL2381291 | 7.439 | O=C(O)C[C@H]1c2c(OC1)cc1OC(c3cc(C4CC4)ccc3)COc1c2 |
| CHEMBL4471403 | 7.438 | Fc1c(OCC(=O)O)ccc(NCc2cc(-c3c(F)cccc3)ccc2)c1 |
| CHEMBL4207273 | 7.432 | O(Cc1cc2c(-c3c(C)ncnc3)csc2cc1)c1ccc([C@@H](C#CC)Cc2[nH]nnn2)cc1 |
| CHEMBL3785764 | 7.430 | Fc1c(CCC(=O)O)ccc(C#C/C(=C/C)/C)c1 |
| CHEMBL4227915 | 7.427 | Fc1c(CCC(=O)O)ccc(OCc2cc(-c3c(C)onc3C)ccc2)c1 |
| CHEMBL4168059 | 7.423 | Clc1cc(-c2[nH]c3c(c2)cc(CCC(=O)O)cc3)ccc1 |
| CHEMBL3894356 | 7.420 | O=C(O)C[C@H](C#CC)c1ccc(OCc2cc(CN(Cc3cscc3)C)ccc2)cc1 |
| CHEMBL2315252 | 7.420 | O=C(O)CCc1ccc(C#Cc2cc(COC)ccc2)cc1 |
| CHEMBL2022247 | 7.416 | O=C(O)CC1c2c(OC1)cc(OCc1cc(-c3c(C)cccc3C)ccc1)cc2 |
| CHEMBL4228673 | 7.416 | O=C(O)CCc1ccc(OCc2cc(-c3c(C)onc3C)ccc2)cc1 |
| CHEMBL2381313 | 7.412 | O=C(O)C[C@H]1c2c(OC1)cc1OC(c3cc(-c4c(C)cc(C)cc4)ccc3)COc1c2 |
| CHEMBL4450552 | 7.412 | Fc1c(OCC(=O)O)ccc(NCc2cc(-c3c(C)cc(OCCC)cc3C)ccc2)c1 |
| CHEMBL3799341 | 7.410 | Clc1cc(COc2cc(F)c(CCC(=O)O)cc2)cc(-c2c(C)onc2C)c1 |
| CHEMBL2022253 | 7.409 | O=C(O)CC1c2c(OC1)cc(OCc1cc(-c3c4c(sc3)cccc4)ccc1)cc2 |
| CHEMBL4213597 | 7.409 | Clc1c(-c2c(C)cc(OCCOC)cc2)c2c(s1)ccc(COc1ccc([C@@H](C#CC)Cc3[nH]nnn3)cc1)c2 |
| CHEMBL2381307 | 7.406 | O=C(O)C[C@H]1c2c(OC1)cc1O[C@H](c3cc(-c4c(C)cccc4)ccc3)COc1c2 |
| CHEMBL4229006 | 7.401 | Clc1c(COc2cc3OCC(CC(=O)O)c3cc2)cc(-c2c(C)onc2C)cc1 |
| CHEMBL2315256 | 7.400 | O=C(O)CCc1ccc(C#Cc2c(C)ccc(C#N)c2)cc1 |
| CHEMBL2048610 | 7.398 | O=C(O)CCc1ccc(OCc2c(C)c(-c3c(C)cccc3C)ccc2)cc1 |
| CHEMBL1829170 | 7.390 | Clc1nc(Cl)cc(C#Cc2ccc(CCC(=O)O)cc2)c1 |
| CHEMBL2315251 | 7.390 | O=C(O)CCc1ccc(C#Cc2c(COC)cccc2)cc1 |
| CHEMBL3287573 | 7.387 | FC(F)(F)c1ccc(-c2sc(COc3ccc([C@H](CC(=O)O)c4n(C)nnn4)cc3)c(C)n2)cc1 |
| CHEMBL4468987 | 7.384 | Fc1c(OCC(=O)O)ccc(NCc2cc(-c3c(C)cc(OCCC)cc3)ccc2)c1 |
| CHEMBL4166922 | 7.380 | S(=O)(=O)(CCCOc1cc(C)c(c(C)c1)-c1cc(COc2cc3OC[C@@H](C(=O)O)c3cc2)ccc1)C |
| CHEMBL4442354 | 7.378 | Fc1c(OCC(=O)O)ccc(NCc2cc(-c3c(C)cc(OCCOC)cc3)ccc2)c1 |
| CHEMBL3905856 | 7.377 | Fc1c(N2C[C@@H](C)[C@H](Oc3ncc(N4[C@@H](CC(=O)O)[C@H](C)C(OC)C4)nc3)CC2)cc(OCC)cc1 |
| CHEMBL1829172 | 7.370 | Clc1nc(Cl)cc(C#Cc2ccc(C3C(C(=O)O)C3)cc2)c1 |
| CHEMBL3629049 | 7.361 | S(=O)(=O)(CCCOc1cc(C)c(-c2cc(COc3cc(F)c(OCC(=O)O)cc3)ccc2)cc1)C |
| CHEMBL1829171 | 7.360 | Clc1c(C)c(C#Cc2ccc(CCC(=O)O)cc2)cc(Cl)n1 |
| CHEMBL4550165 | 7.341 | S(=O)(=O)(CCCOc1cc(C)c(-c2cc(CNc3cc(F)c(OCC(=O)O)cc3)ccc2)cc1)C |
| CHEMBL4288001 | 7.337 | Clc1cc2O[C@@H](COc3ccc([C@@H](C#CC)CC(=O)O)cc3)COc2cc1 |
| CHEMBL2381295 | 7.333 | S(=O)(=O)(CCCOc1cc(C)c(c(C)c1)-c1cc([C@H]2Oc3c(OC2)cc2[C@H](CC(=O)O)COc2c3)c(F)cc1)C |
| CHEMBL2315530 | 7.330 | O=C(O)CCc1ccc(C#Cc2c(OCC#N)cccc2)cc1 |
| CHEMBL4217477 | 7.330 | S(=O)(=O)(CC(=O)O)c1ccc(OCc2cc(-c3c(C)cccc3)ccc2)cc1 |
| CHEMBL3781077 | 7.329 | O=C(O)CC1c2c(OC1)cc(OCc1cc(-n3c(C)ccc3C)ccc1)cc2 |
| CHEMBL4215544 | 7.328 | O(CCOc1cc(C)c(-c2c(C)sc3c2cc(COc2ccc([C@@H](C#CC)Cc4[nH]nnn4)cc2)cc3)cc1)C |
| CHEMBL3787072 | 7.320 | Fc1c(CCC(=O)O)ccc(C#C/C=C\C)c1 |
| CHEMBL3781477 | 7.312 | Fc1ccc(-c2sc(COc3cc4OCC(CC(=O)O)c4cc3)c(C)n2)cc1 |
| CHEMBL2022248 | 7.310 | O=C(O)C1Cc2c(OCC1)cc(OCc1cc(-c3c(C)cccc3C)ccc1)cc2 |
| CHEMBL3604054 | 7.310 | Clc1c(Cl)ccc(Oc2ccc(C(OCC)CC(=O)O)cc2)c1 |
| CHEMBL3628755 | 7.304 | Fc1c(OCC(=O)O)ccc(OCc2cc(-c3c(C)cc(OC)cc3C)ccc2)c1 |
| CHEMBL4204628 | 7.290 | Clc1c(-c2cc(COc3ccc(S(=O)(=O)CC(=O)O)cc3)ccc2)ccc(OCC)c1 |
| CHEMBL4574808 | 7.287 | Fc1c(OCC(=O)O)ccc(NCc2cc(-c3c(C)cc(OCCOC)cc3C)ccc2)c1 |
| CHEMBL4461611 | 7.287 | Clc1c(-c2ccccc2)cc(CNc2cc(F)c(OCC(=O)O)cc2)cc1 |
| CHEMBL4209622 | 7.280 | S(=O)(=O)(CC(=O)O)c1ccc(OCc2cc(-c3c(C(F)(F)F)cccc3)ccc2)cc1 |
| CHEMBL2381305 | 7.277 | O=C(O)C[C@H]1c2c(OC1)cc1O[C@H](c3cc(-c4c(C)cccc4C)ccc3)COc1c2 |
| CHEMBL3604045 | 7.276 | O=C(O)CC(OCC)c1ccc(OCc2cc(C#N)ccc2)cc1 |
| CHEMBL3798881 | 7.275 | Fc1c(CCC(=O)O)ccc(OCc2cc(OC)cc(-c3c(C)onc3C)c2)c1 |
| CHEMBL4226180 | 7.270 | O=C(O)CC1c2c(OC1)cc(OCc1c(C)ccc(-c3c(C)onc3C)c1)cc2 |
| CHEMBL4213548 | 7.268 | O(CCOc1c(C)c(-c2c3c(sc2)ccc(COc2ccc([C@@H](C#CC)Cc4[nH]nnn4)cc2)c3)ccc1)C |
| CHEMBL4160141 | 7.263 | Clc1c(-c2[nH]c3c(c2)cc(CCC(=O)O)cc3)cccc1 |
| CHEMBL2381281 | 7.262 | Brc1cc(C2Oc3c(OC2)cc2[C@H](CC(=O)O)COc2c3)ccc1 |
| CHEMBL2381293 | 7.253 | S(=O)(=O)(CCCOc1cc(C)c(c(C)c1)-c1cc([C@H]2Oc3c(OC2)cc2[C@H](CC(=O)O)COc2c3)ccc1)C |
| CHEMBL2381302 | 7.250 | Clc1c(-c2cc(c(F)cc2)C2Oc3c(OC2)cc2[C@H](CC(=O)O)COc2c3)ccc(OCCCS(=O)(=O)C)c1 |
| CHEMBL2381310 | 7.246 | Clc1c(-c2cc([C@@H]3Oc4c(OC3)cc3[C@H](CC(=O)O)COc3c4)ccc2)cccc1 |
| CHEMBL4212170 | 7.240 | S(=O)(=O)(CC(=O)O)c1ccc(OCc2cc(-c3c(C)cc(OC)cc3C)ccc2)cc1 |
| CHEMBL3600993 | 7.237 | O=C(O)CCc1ccc(OCc2c(C)oc(-c3ccccc3)n2)cc1 |
| CHEMBL4577954 | 7.233 | Fc1c(OCC(=O)O)ccc(NCc2cc(-c3ccccc3)ccc2)c1 |
| CHEMBL4537797 | 7.223 | S(=O)(=O)(CCCOc1cc(C)c(c(C)c1)-c1cc(CNc2cc(F)c(OCC(=O)O)cc2)ccc1)C |
| CHEMBL3781733 | 7.213 | O=C(O)CC1c2c(OC1)cc(OCc1cc(-n3c(C)ccc3C)c(OCC)cc1)cc2 |
| CHEMBL4240026 | 7.211 | Fc1c(OCC(=O)O)ccc(OCc2cc(-c3c(C)cc(OCCCO[N+](=O)[O-])cc3C)ccc2)c1 |
| CHEMBL2381314 | 7.210 | O=C(O)C[C@H]1c2c(OC1)cc1OC(c3cc(-c4c(C)cc(C)cc4C)ccc3)COc1c2 |
| CHEMBL3353742 | 7.206 | S(=O)(=O)(CCCOc1cc(C)c(c(C)c1)-c1cc(COc2cc(F)c(OCC(=O)O)cc2)ccc1)C |
| CHEMBL3604046 | 7.201 | O=[N+]([O-])c1cc(COc2ccc(C(OCC)CC(=O)O)cc2)ccc1 |
| CHEMBL4210878 | 7.201 | Clc1c2scc(-c3c(C)cccc3)c2cc(COc2ccc([C@@H](C#CC)Cc3[nH]nnn3)cc2)c1 |
| CHEMBL4215675 | 7.200 | S(=O)(=O)(CC(=O)O)c1ccc(OCc2cc(-c3c(C)cc(OCC)cc3C)ccc2)cc1 |
| CHEMBL2380322 | 7.196 | FC(F)(F)c1cc(F)cc(C2Oc3c(OC2)cc2[C@H](CC(=O)O)COc2c3)c1 |
| CHEMBL3780456 | 7.188 | Fc1cc(-c2sc(COc3cc4OCC(CC(=O)O)c4cc3)c(C)n2)ccc1 |
| CHEMBL3287572 | 7.187 | FC(F)(F)c1ccc(-c2sc(COc3ccc([C@H](CC(=O)O)c4n(C)ncn4)cc3)c(C)n2)cc1 |
| CHEMBL3604053 | 7.187 | Clc1c(Cl)ccc(CCOc2ccc(C(OCC)CC(=O)O)cc2)c1 |
| CHEMBL2381311 | 7.184 | O=C(O)C[C@H]1c2c(OC1)cc1OC(c3cc(-c4ccccc4)ccc3)COc1c2 |
| CHEMBL3628756 | 7.181 | Fc1c(OCC(=O)O)ccc(OCc2cc(-c3c(C)cc(OCC)cc3C)ccc2)c1 |
| CHEMBL4209845 | 7.180 | S(=O)(=O)(CC(=O)O)c1ccc(OCc2cc(-c3c(C)cccc3C)ccc2)cc1 |
| CHEMBL2381309 | 7.176 | Clc1c(-c2cc([C@H]3Oc4c(OC3)cc3[C@H](CC(=O)O)COc3c4)ccc2)cccc1 |
| CHEMBL4210243 | 7.174 | O(Cc1cc2c(-c3n(C)ncc3)csc2cc1)c1ccc([C@@H](C#CC)Cc2[nH]nnn2)cc1 |
| CHEMBL2381296 | 7.171 | S(=O)(=O)(CCCOc1cc(C)c(c(C)c1)-c1cc([C@@H]2Oc3c(OC2)cc2[C@H](CC(=O)O)COc2c3)c(F)cc1)C |
| CHEMBL4579901 | 7.167 | FC(F)(F)c1ccc(-c2sc(COc3cc4OC[C@@H](CC(=O)O)c4cc3)c(C)n2)cc1 |
| CHEMBL3780232 | 7.164 | O=C(O)CC1c2c(OC1)cc(OCc1c(C)nc(-c3cscc3)s1)cc2 |
| CHEMBL2022249 | 7.161 | O=C(O)CC1c2c(cc(OCc3cc(-c4c(C)cccc4C)ccc3)cc2)C1 |
| CHEMBL3287575 | 7.161 | FC(F)(F)c1ccc(-c2sc(COc3ccc([C@H](CC(=O)O)c4occn4)cc3)c(C)n2)cc1 |
| CHEMBL3427706 | 7.161 | O=C(O)C[C@H](OCC)c1ccc(O[C@@H]2c3c(cccc3)CC2)cc1 |
| CHEMBL3797383 | 7.161 | Fc1c(CCC(=O)O)ccc(OCc2cc(OCC)cc(-c3c(C)onc3C)c2)c1 |
| CHEMBL4210719 | 7.160 | S(=O)(=O)(CC(=O)O)c1ccc(OCc2cc(-c3c(C)cc(OCCC)cc3C)ccc2)cc1 |
| CHEMBL4561122 | 7.157 | Fc1c(OCC(=O)O)ccc(NCc2cc(-c3c(C)cc(O)cc3)ccc2)c1 |
| CHEMBL2315553 | 7.150 | O=C(O)CCc1ccc(C#Cc2cc(OC)ccc2)cc1 |
| CHEMBL3973075 | 7.149 | FC(F)(F)C=1[C@@H](C)[C@H](CC(=O)O)N(c2ccc(O[C@H]3[C@H](C)CN(c4c(C)ccc(OC)c4)CC3)cc2)N=1 |
| CHEMBL3286409 | 7.137 | FC(F)(F)c1ccc(-c2sc(COc3ccc([C@H](CC(=O)O)c4n(C)ccn4)cc3)c(C)n2)cc1 |
| CHEMBL2381318 | 7.135 | Fc1c(-c2cc(C3Oc4c(OC3)cc3[C@H](CC(=O)O)COc3c4)ccc2)cccc1 |
| CHEMBL3780334 | 7.134 | O=C(O)CC1c2c(OC1)cc(OCc1c(C)nc(-c3ccc(C)cc3)s1)cc2 |
| CHEMBL4214530 | 7.130 | S(=O)(=O)(CC(=O)O)c1ccc(OCc2cc(-c3c(C(C)C)cccc3)ccc2)cc1 |
| CHEMBL2381280 | 7.128 | FC(F)(F)c1c(-c2cc(C3Oc4c(OC3)cc3[C@H](CC(=O)O)COc3c4)ccc2)cccc1 |
| CHEMBL2381317 | 7.127 | Clc1c(-c2cc(C3Oc4c(OC3)cc3[C@H](CC(=O)O)COc3c4)ccc2)ccc(F)c1 |
| CHEMBL1829156 | 7.120 | O=C(O)CCc1ccc(C#Cc2c(C)ccs2)cc1 |
| CHEMBL4246335 | 7.117 | Fc1c(OCC(=O)O)ccc(OCc2cc(-c3c(C)cc(OCCO[N+](=O)[O-])cc3C)ccc2)c1 |
| CHEMBL3797784 | 7.101 | Fc1c(CCC(=O)O)ccc(OCc2cc(OC(C)C)cc(-c3c(C)onc3C)c2)c1 |
| CHEMBL1829169 | 7.100 | Clc1cc2nccc(C#Cc3ccc(CCC(=O)O)cc3)c2cc1 |
| CHEMBL4208237 | 7.100 | S(=O)(=O)(CC(=O)O)c1ccc(OCc2cc(-c3c(C)cc(OCC4CC4)cc3C)ccc2)cc1 |
| CHEMBL3787463 | 7.090 | Fc1c(CCC(=O)O)ccc(C#CC(=C)C)c1 |
| CHEMBL3427707 | 7.086 | O=C(O)C[C@H](OCC)c1ccc(OC2Cc3c(cccc3)C2)cc1 |
| CHEMBL2381297 | 7.084 | S(=O)(=O)(CCCOc1c(F)c(C)c(c(C)c1)-c1cc([C@H]2Oc3c(OC2)cc2[C@H](CC(=O)O)COc2c3)ccc1)C |
| CHEMBL4204776 | 7.081 | O(Cc1cc2c(N3C(C)CCCC3)csc2cc1)c1ccc([C@@H](C#CC)Cc2[nH]nnn2)cc1 |
| CHEMBL2315548 | 7.080 | Brc1c(C#Cc2ccc(CCC(=O)O)cc2)cccc1 |
| CHEMBL3786972 | 7.080 | O=C(O)CCc1ccc(C#CC(=C(C)C)C)cc1 |
| CHEMBL2022579 | 7.076 | O=C(O)CC1Cc2c(ccc(OCc3cc(-c4c(C)cc(OCCOCC)cc4C)ccc3)c2)C1 |
| CHEMBL4216026 | 7.071 | O(CCOc1cc(c(C)cc1)-c1c2c(sc1)ccc(COc1ccc([C@@H](C#CC)Cc3[nH]nnn3)cc1)c2)C |
| CHEMBL3781162 | 7.068 | Clc1ccc(-c2sc(COc3cc4OCC(CC(=O)O)c4cc3)c(C)n2)cc1 |
| CHEMBL4458669 | 7.064 | Fc1c(OCC(=O)O)ccc(NCc2cc(-c3c(C(C)C)cccc3)ccc2)c1 |
| CHEMBL2381315 | 7.056 | O=C(O)C[C@H]1c2c(OC1)cc1OC(c3cc(-c4c(C)ccc(C)c4)ccc3)COc1c2 |
| CHEMBL4206579 | 7.056 | O(Cc1cc2c(-c3c(C)nccc3)csc2cc1)c1ccc([C@@H](C#CC)Cc2[nH]nnn2)cc1 |
| CHEMBL3604042 | 7.051 | O=C(O)CC(OCC)c1ccc(OCc2cc(OC)ccc2)cc1 |
| CHEMBL2315545 | 7.050 | O=C(O)CCc1ccc(C#Cc2c(CC)cccc2)cc1 |
| CHEMBL3799443 | 7.049 | Fc1c(CCC(=O)O)ccc(OCc2cc(OCCCC)cc(-c3c(C)onc3C)c2)c1 |
| CHEMBL4285582 | 7.046 | O=C(O)C[C@H](C#CC)c1ccc(OC[C@@H]2Oc3c(OC2)cccc3)cc1 |
| CHEMBL3798609 | 7.041 | Fc1c(CCC(=O)O)ccc(OCc2cc(OCC3CC3)cc(-c3c(C)onc3C)c2)c1 |
| CHEMBL2022582 | 7.041 | O=C(O)CC1OCc2c1ccc(OCc1cc(-c3c(C)cc(OCCOCC)cc3C)ccc1)c2 |
| CHEMBL3353741 | 7.033 | Clc1c(OCC(=O)O)ccc(OCc2cc(-c3c(C)cc(OCCCS(=O)(=O)C)cc3C)ccc2)c1 |
| CHEMBL2315539 | 7.030 | O=C(O)[C@@H]1[C@@H](c2ccc(C#Cc3cc(C)ccc3)cc2)C1 |
| CHEMBL4250135 | 7.028 | Fc1c(OCC(=O)O)ccc(OCc2cc(-c3c(C)cc(OCCCCO[N+](=O)[O-])cc3C)ccc2)c1 |
| CHEMBL2381299 | 7.023 | Clc1c(-c2cc([C@H]3Oc4c(OC3)cc3[C@H](CC(=O)O)COc3c4)ccc2)ccc(OCCCS(=O)(=O)C)c1 |
| CHEMBL2315555 | 7.020 | FC(F)(F)Oc1cc(C#Cc2ccc(CCC(=O)O)cc2)ccc1 |
| CHEMBL4217180 | 7.020 | S(=O)(=O)(CC(=O)O)c1ccc(OCc2cc(-c3c(C)cc(OCC(C)C)cc3C)ccc2)cc1 |
| CHEMBL3780211 | 7.015 | FC(F)(F)c1ccc(-c2sc(COc3cc4OCC(CC(=O)O)c4cc3)c(C)n2)cc1 |
| CHEMBL3798213 | 7.015 | Fc1c(CCC(=O)O)ccc(OCc2cc(OCCC)cc(-c3c(C)onc3C)c2)c1 |
| CHEMBL4218763 | 7.010 | S(=O)(=O)(CC(=O)O)c1ccc(OCc2cc(Oc3ccccc3)ccc2)cc1 |
| CHEMBL3604038 | 7.009 | Clc1c(Cl)ccc(COc2cc(Cl)c(C(OCC)CC(=O)O)cc2)c1 |
| CHEMBL2048625 | 7.004 | S(=O)(=O)(CCOc1cc(C)c(c(C)c1)-c1cc(CNc2cc(F)c(CCC(=O)O)cc2)ccc1)CC |
| CHEMBL3604043 | 7.000 | O=C(O)CC(OCC)c1ccc(OCc2cc(OCOC)ccc2)cc1 |
| CHEMBL4288867 | 7.000 | Clc1cc2OC(COc3ccc(C(C#CC)CC(=O)O)cc3)COc2cc1 |
| CHEMBL3798992 | 6.991 | Fc1c(CCC(=O)O)ccc(OCc2cc(OC3CCCC3)cc(-c3c(C)onc3C)c2)c1 |
| CHEMBL3780725 | 6.991 | O=C(O)CC1c2c(OC1)cc(OCc1c(C)nc(-c3ccccc3)s1)cc2 |
| CHEMBL3628763 | 6.985 | Fc1c(OCC(=O)O)ccc(OCc2cc(-c3c(C)cc(OCCOC)cc3C)ccc2)c1 |
| CHEMBL4225643 | 6.984 | O=C(O)CC1c2c(OC1)cc(OCc1cc(-c3c(C)n(C)nc3C)ccc1)cc2 |
| CHEMBL2381292 | 6.978 | Brc1cc(F)cc(C2Oc3c(OC2)cc2[C@H](CC(=O)O)COc2c3)c1 |
| CHEMBL3628757 | 6.976 | Fc1c(OCC(=O)O)ccc(OCc2cc(-c3c(C)cc(OCCC)cc3C)ccc2)c1 |
| CHEMBL4218110 | 6.971 | O(Cc1cc2c(C3CCCCC3)csc2cc1)c1ccc([C@@H](C#CC)Cc2[nH]nnn2)cc1 |
| CHEMBL3629046 | 6.961 | Fc1c(OCC(=O)O)ccc(OCc2cc(-c3c(C)cc(OCCCO)cc3C)ccc2)c1 |
| CHEMBL4283655 | 6.959 | Clc1cc2OC[C@H](COc3ccc([C@@H](C#CC)CC(=O)O)cc3)Oc2cc1 |
| CHEMBL3628762 | 6.957 | Fc1c(OCC(=O)O)ccc(OCc2cc(-c3c(C)cc(O)cc3C)ccc2)c1 |
| CHEMBL2381283 | 6.954 | Brc1cc([C@@H]2Oc3c(OC2)cc2[C@H](CC(=O)O)COc2c3)ccc1 |
| CHEMBL4463798 | 6.951 | Fc1c(OCC(=O)O)ccc(OCc2c(C)nc(-c3ccc(F)cc3)s2)c1 |
| CHEMBL3798642 | 6.943 | FC(F)(F)c1cc(COc2cc(F)c(CCC(=O)O)cc2)cc(-c2c(C)onc2C)c1 |
| CHEMBL3931296 | 6.927 | O=C(O)C[C@H](C#CC)c1ccc(OCc2cc(CNCc3cscc3)ccc2)cc1 |
| CHEMBL3781145 | 6.926 | O=C(O)CC1c2c(OC1)cc(OCc1c(C)nc(-c3cc(C)ccc3)s1)cc2 |
| CHEMBL3927519 | 6.924 | O=C(O)C[C@H](C#CC)c1ccc(OCC2=Cn3nc(-c4c(C)cccc4C)nc3C=C2)cc1 |
| CHEMBL3604060 | 6.921 | O=C(O)C[C@H](OCC)c1ccc(Oc2noc3c(C)cccc23)cc1 |
| CHEMBL4279126 | 6.921 | O=C(O)C[C@H](C#CC)c1ccc(OC[C@@H]2Oc3c(OC2)ccc(C)c3)cc1 |
| CHEMBL1829165 | 6.920 | Fc1c(C)c(C#Cc2ccc(CCC(=O)O)cc2)ccn1 |
| CHEMBL2381298 | 6.918 | S(=O)(=O)(CCCOc1c(F)c(C)c(c(C)c1)-c1cc([C@@H]2Oc3c(OC2)cc2[C@H](CC(=O)O)COc2c3)ccc1)C |
| CHEMBL3353261 | 6.917 | S(=O)(=O)(CCCOc1cc(C)c(c(C)c1)-c1cc(COc2ccc(OCC(=O)O)cc2)ccc1)C |
| CHEMBL2381301 | 6.912 | S(=O)(=O)(CCCOc1cc(C)c(-c2cc(C3Oc4c(OC3)cc3[C@H](CC(=O)O)COc3c4)ccc2)cc1)C |
| CHEMBL2315541 | 6.910 | O=C(O)CCc1ccc(C#Cc2c(C)ccc(C)c2)cc1 |
| CHEMBL4207283 | 6.910 | O=C(O)CC1c2c(SC1)cc(OCc1cc(-c3c(C)cc(OC)cc3C)ccc1)cc2 |
| CHEMBL3781779 | 6.909 | O=C(O)CC1c2c(OC1)cc(OCc1c(C)nc(-c3ccc(OC)cc3)s1)cc2 |
| CHEMBL2381304 | 6.903 | Clc1c(-c2c(F)ccc(C3Oc4c(OC3)cc3[C@H](CC(=O)O)COc3c4)c2)ccc(OCCCS(=O)(=O)C)c1 |
| CHEMBL4205241 | 6.900 | S(=O)(=O)(CC(=O)O)c1ccc(OCc2cc(-c3c(C)cc(OCCOC)cc3C)ccc2)cc1 |
| CHEMBL4218236 | 6.890 | S(=O)(=O)(CC(=O)O)c1ccc(OCc2cc(-n3c(C)ccc3C)ccc2)cc1 |
| CHEMBL4277181 | 6.886 | O=C(O)C[C@H](C#CC)c1ccc(OC[C@@H]2Oc3c(OC2)ccc(OC)c3)cc1 |
| CHEMBL2381316 | 6.879 | O=C(O)C[C@H]1c2c(OC1)cc1OC(c3cc(-c4cc(C)cc(C)c4)ccc3)COc1c2 |
| CHEMBL4216016 | 6.873 | O(Cc1cc(C)c2scc(-c3c(C)cccc3)c2c1)c1ccc([C@@H](C#CC)Cc2[nH]nnn2)cc1 |
| CHEMBL1829166 | 6.870 | Clc1c(C)c(C#Cc2ccc(CCC(=O)O)cc2)ccn1 |
| CHEMBL3780713 | 6.862 | Clc1cc(-c2sc(COc3cc4OCC(CC(=O)O)c4cc3)c(C)n2)ccc1 |
| CHEMBL2315538 | 6.860 | O=C(O)[C@@H]1[C@@H](c2ccc(C#Cc3c(C)cccc3)cc2)C1 |
| CHEMBL2315543 | 6.860 | Clc1cc(C)c(C#Cc2ccc(CCC(=O)O)cc2)cc1 |
| CHEMBL2022580 | 6.854 | O=C(O)C1Cc2c(cc(OCc3cc(-c4c(C)cc(OCCOCC)cc4C)ccc3)cc2)CC1 |
| CHEMBL3905435 | 6.854 | O=C(O)C[C@H](C#CC)c1ccc(OCc2cc(CN(Cc3cscc3)C3CCN(C)CC3)ccc2)cc1 |
| CHEMBL3800255 | 6.845 | Fc1c(CCC(=O)O)ccc(OCc2cc(OCC(C)C)cc(-c3c(C)onc3C)c2)c1 |
| CHEMBL2315537 | 6.840 | O=C(O)[C@@H]1[C@@H](c2ccc(C#Cc3ccccc3)cc2)C1 |
| CHEMBL2381300 | 6.838 | Clc1c(-c2cc([C@@H]3Oc4c(OC3)cc3[C@H](CC(=O)O)COc3c4)ccc2)ccc(OCCCS(=O)(=O)C)c1 |
| CHEMBL3958983 | 6.836 | O=C(O)C[C@H](C#CC)c1ccc(OCC=2c3n(ncn3)C=C(c3c(C)cccc3)C=2)cc1 |
| CHEMBL3604056 | 6.833 | Clc1c(Cl)ccc(COc2cc(C(OCC)CC(=O)O)ccc2)c1 |
| CHEMBL4573403 | 6.827 | FC(F)(F)c1ccc(-c2sc(COc3cc(F)c(OCC(=O)O)cc3)c(C)n2)cc1 |
| CHEMBL4283222 | 6.824 | Clc1c(Cl)cc2OC[C@H](COc3ccc([C@@H](C#CC)CC(=O)O)cc3)Oc2c1 |
| CHEMBL2315547 | 6.820 | Clc1c(C#Cc2ccc(CCC(=O)O)cc2)cccc1 |
| CHEMBL3353738 | 6.814 | S(=O)(=O)(CCCOc1cc(C)c(c(C)c1)-c1cc(COc2cc(C)c(OCC(=O)O)cc2)ccc1)C |
| CHEMBL4534812 | 6.807 | Fc1c(OCC(=O)O)ccc(OCc2c(C)nc(-c3ccc(C)cc3)s2)c1 |
| CHEMBL4226029 | 6.804 | O=C(O)CC1c2c(OC1)cc(OCc1cc(-c3c(C)ccs3)ccc1)cc2 |
| CHEMBL3600994 | 6.796 | Clc1cc(-c2occ(COc3ccc(CCC(=O)O)cc3)n2)ccc1 |
| CHEMBL4210139 | 6.770 | S(=O)(=O)(CCCOc1cc(C)c(c(C)c1)-c1cc(COc2ccc(S(=O)(=O)CC(=O)O)cc2)ccc1)C |
| CHEMBL4291567 | 6.770 | Clc1c2OC[C@H](COc3ccc([C@@H](C#CC)CC(=O)O)cc3)Oc2ccc1 |
| CHEMBL1829164 | 6.750 | Fc1ncc(C)c(C#Cc2ccc(CCC(=O)O)cc2)c1 |
| CHEMBL4213829 | 6.750 | S(=O)(=O)(CC(=O)O)c1ccc(OCc2ccc(OC/C(=N\OC)/c3ccccc3)cc2)cc1 |
| CHEMBL2022254 | 6.745 | O=C(O)CC1c2c(OC1)cc(OCc1cc(-c3cc4c(scc4)cc3)ccc1)cc2 |
| CHEMBL4456869 | 6.733 | Fc1c(OCC(=O)O)ccc(OCc2c(C)nc(-c3cc(F)ccc3)s2)c1 |
| CHEMBL1829162 | 6.730 | O=C(O)CCc1ccc(C#Cc2cc(-c3ccccc3)ncc2)cc1 |
| CHEMBL2315248 | 6.730 | O=C(O)CCc1ccc(C#Cc2c(C(=O)C)cccc2)cc1 |
| CHEMBL3604064 | 6.721 | O=C(O)C[C@H](OCC)c1ccc(OC2c3c(c(OC)ccc3)CC2)cc1 |
| CHEMBL2381288 | 6.720 | Clc1c(Cl)ccc([C@@H]2Oc3c(OC2)cc2[C@H](CC(=O)O)COc2c3)c1 |
| CHEMBL2315529 | 6.720 | O=C(O)CCc1ccc(C#Cc2cc(CC#N)ccc2)cc1 |
| CHEMBL4210871 | 6.720 | S(=O)(=O)(CC(=O)O)c1ccc(OCc2c(C)nc(-c3ccc(F)cc3)s2)cc1 |
| CHEMBL4214140 | 6.710 | S(CC(=O)O)c1ccc(OCc2cc(-c3c(C)cccc3C)ccc2)cc1 |
| CHEMBL4206179 | 6.710 | S(=O)(=O)(CC(=O)O)c1ccc(OCc2cc(-c3c(OC)cccc3)ccc2)cc1 |
| CHEMBL4516052 | 6.706 | O=C(O)COc1ccc(NCc2cc(-c3c(C)cc(OCC)cc3C)ccc2)cc1 |
| CHEMBL2315535 | 6.700 | O=C(O)CC(C)c1ccc(C#Cc2ccccc2)cc1 |
| CHEMBL3786524 | 6.700 | O=C(O)CCc1ccc(C#CC2=CCCCC2)cc1 |
| CHEMBL4290397 | 6.699 | Brc1c(Cl)cc2O[C@@H](COc3ccc([C@@H](C#CC)CC(=O)O)cc3)COc2c1 |
| CHEMBL2381303 | 6.688 | Clc1c(-c2cc(F)cc(C3Oc4c(OC3)cc3[C@H](CC(=O)O)COc3c4)c2)ccc(OCCCS(=O)(=O)C)c1 |
| CHEMBL3786526 | 6.680 | Fc1c(CCC(=O)O)ccc(C#C/C=C/C)c1 |
| CHEMBL3601000 | 6.678 | O=C(O)CCc1ccc(OCc2onc(-c3ccc(CC)cc3)n2)cc1 |
| CHEMBL3604034 | 6.678 | Clc1c(Cl)ccc(COc2c(F)cc(C(OCC)CC(=O)O)cc2)c1 |
| CHEMBL4293798 | 6.678 | Clc1c(F)cc2OC[C@H](COc3ccc([C@@H](C#CC)CC(=O)O)cc3)Oc2c1 |
| CHEMBL4279826 | 6.678 | Fc1cc2O[C@@H](COc3ccc([C@@H](C#CC)CC(=O)O)cc3)COc2cc1 |
| CHEMBL4287731 | 6.678 | Brc1cc2O[C@@H](COc3ccc([C@@H](C#CC)CC(=O)O)cc3)COc2cc1 |
| CHEMBL4215289 | 6.670 | S(=O)(=O)(CC(=O)O)c1ccc(OCc2c(C)nc(-c3cc(C)ccc3)s2)cc1 |
| CHEMBL4211760 | 6.670 | Clc1c(-c2c(C)onc2C)cc(COc2ccc(S(=O)(=O)CC(=O)O)cc2)cc1 |
| CHEMBL3797889 | 6.670 | Fc1c(CCC(=O)O)ccc(OCc2cc(C#Cc3c(C)onc3C)ccc2)c1 |
| CHEMBL2315552 | 6.660 | O=C(O)CCc1ccc(C#Cc2c(OC)cccc2)cc1 |
| CHEMBL2381286 | 6.658 | Clc1cc([C@@H]2Oc3c(OC2)cc2[C@H](CC(=O)O)COc2c3)ccc1 |
| CHEMBL2315554 | 6.650 | FC(F)(F)Oc1c(C#Cc2ccc(CCC(=O)O)cc2)cccc1 |
| CHEMBL4469534 | 6.647 | Fc1c(OCC(=O)O)ccc(NCc2cc(-c3c(C)onc3C)ccc2)c1 |
| CHEMBL4202936 | 6.630 | O=C(O)CC1c2c(C(=O)C1)cc(OCc1cc(-c3c(C)cc(OC)cc3C)ccc1)cc2 |
| CHEMBL4206875 | 6.630 | Clc1c(COc2ccc(S(=O)(=O)CC(=O)O)cc2)cc(-c2c(C)onc2C)cc1 |
| CHEMBL4581313 | 6.607 | Fc1c(OCC(=O)O)ccc(OCc2c(C)nc(-c3cc(C)ccc3)s2)c1 |
| CHEMBL4535460 | 6.600 | Fc1ccc(-c2sc(COc3ccc(CCC(=O)O)cc3)c(C)n2)cc1 |
| CHEMBL1829155 | 6.600 | O=C(O)CCc1ccc(C#Cc2sccc2)cc1 |
| CHEMBL4210351 | 6.600 | Clc1cc(-c2sc(COc3ccc(S(=O)(=O)CC(=O)O)cc3)c(C)n2)ccc1 |
| CHEMBL4214028 | 6.590 | S(=O)(=O)(CC(=O)O)c1ccc(OCc2cc(-c3c(F)cccc3)ccc2)cc1 |
| CHEMBL4281717 | 6.585 | Clc1cc2O[C@H](COc3ccc([C@@H](C#CC)CC(=O)O)cc3)COc2cc1 |
| CHEMBL2315531 | 6.580 | O=C(O)CCc1ccc(C#Cc2cc(OCC#N)ccc2)cc1 |
| CHEMBL4206505 | 6.570 | S(=O)(=O)(CC(=O)O)c1ccc(OCc2cc(-c3c(C)onc3C)ccc2)cc1 |
| CHEMBL4205991 | 6.560 | S(=O)(=O)(CC(=O)O)c1ccc(OCc2c(C)nc(-c3cc(F)ccc3)s2)cc1 |
| CHEMBL4294274 | 6.553 | O=C(O)C[C@H](C#CC)c1ccc(OC[C@@H]2Oc3c(OC2)ccc(OC(C)C)c3)cc1 |
| CHEMBL2381294 | 6.550 | S(=O)(=O)(CCCOc1cc(C)c(c(C)c1)-c1cc([C@@H]2Oc3c(OC2)cc2[C@H](CC(=O)O)COc2c3)ccc1)C |
| CHEMBL4205774 | 6.540 | S(=O)(=O)(CC(=O)O)c1ccc(OCc2cc(-c3ccccc3)ccc2)cc1 |
| CHEMBL1829159 | 6.530 | Clc1nccc(C#Cc2ccc(CCC(=O)O)cc2)c1 |
| CHEMBL3601001 | 6.523 | O=C(O)CCc1ccc(OCc2onc(-c3ccc(C)cc3)n2)cc1 |
| CHEMBL389699 | 6.520 | O=C(O)CC(c1ccc(OC[C@H](CC)C)cc1)c1ccccc1 |
| CHEMBL2315540 | 6.510 | O=C(O)[C@@H]1[C@@H](c2ccc(C#Cc3ccc(C)cc3)cc2)C1 |
| CHEMBL1829167 | 6.500 | Clc1c(CC=C)c(C#Cc2ccc(CCC(=O)O)cc2)ccn1 |
| CHEMBL4445519 | 6.488 | FC(F)(F)c1ccc(-c2sc(COc3ccc(CCC(=O)O)cc3)c(C)n2)cc1 |
| CHEMBL4210415 | 6.480 | S(=O)(=O)(CC(=O)O)c1ccc(OCc2c(C)nc(-c3ccccc3)s2)cc1 |
| CHEMBL2315549 | 6.470 | FC(F)(F)c1c(C#Cc2ccc(CCC(=O)O)cc2)cccc1 |
| CHEMBL2315536 | 6.460 | O=C(O)CC1c2c(cc(C#Cc3ccccc3)cc2)CC1 |
| CHEMBL4206915 | 6.440 | S(=O)(=O)(CC(=O)O)c1ccc(OCc2c(C)c(-c3c(C)onc3C)ccc2)cc1 |
| CHEMBL4436948 | 6.427 | Fc1c(OCC(=O)O)ccc(OCc2c(C)nc(-c3ccccc3)s2)c1 |
| CHEMBL3900797 | 6.420 | O(CCO)c1cc(C)c(-c2cc(COc3ccc(CN4C(=O)NC(=O)O4)cc3)ccc2)cc1 |
| CHEMBL4281238 | 6.420 | O=C(O)C#Cc1ccc(OCc2cc(-c3c(C)cccc3C)ccc2)cc1 |
| CHEMBL3787580 | 6.410 | O=C(O)CCc1ccc(C#CCc2ccccc2)cc1 |
| CHEMBL3353262 | 6.410 | S(=O)(=O)(CCCOc1cc(C)c(c(C)c1)-c1cc(COc2ccc(OC(C(=O)O)C)cc2)ccc1)C |
| CHEMBL4464317 | 6.401 | Fc1c(OCC(=O)O)ccc(NCc2ccc(OC/C(=N\OC)/c3ccccc3)cc2)c1 |
| CHEMBL2315542 | 6.380 | O=C(O)CCc1ccc(C#Cc2c(C)cccc2C)cc1 |
| CHEMBL3785544 | 6.380 | O=C(O)CCc1ccc(C#CC(=C)C)cc1 |
| CHEMBL2315254 | 6.370 | S(=O)(=O)(CCCCOc1cc(C#Cc2ccc(CCC(=O)O)cc2)ccc1)C |
| CHEMBL3785413 | 6.370 | Fc1c(CCC(=O)O)ccc(C#C/C(=C\CO)/C)c1 |
| CHEMBL4211807 | 6.370 | S(=O)(=O)(CC(=O)O)c1ccc(OCc2c(C)ccc(-c3c(C)onc3C)c2)cc1 |
| CHEMBL2315551 | 6.350 | O=C(O)CCc1ccc(C#Cc2cc(O)ccc2)cc1 |
| CHEMBL4164008 | 6.350 | O=C(O)CCc1cc2c([nH]c(-c3c(C)cccc3)c2)cc1 |
| CHEMBL4211064 | 6.340 | S(=O)(=O)(CC(=O)O)c1ccc(OCc2cc(-c3c(C#N)cccc3)ccc2)cc1 |
| CHEMBL2315255 | 6.330 | O=C(O)CCc1ccc(C#Cc2c(C#N)ccc(C)c2)cc1 |
| CHEMBL1829161 | 6.320 | O=C(O)CCc1ccc(C#Cc2cc(Oc3ccccc3)ncc2)cc1 |
| CHEMBL3787110 | 6.310 | O=C(O)CCc1ccc(C#CC2CCCC2)cc1 |
| CHEMBL3601002 | 6.301 | Clc1ccc(-c2nc(C(Oc3ccc(CCC(=O)O)cc3)C)on2)cc1 |
| CHEMBL3798520 | 6.296 | Fc1c(CCC(=O)O)ccc(OCc2nc(-c3c(C)onc3C)ccc2)c1 |
| CHEMBL2022581 | 6.292 | O=C(O)CC1Cc2c(cc(OCc3cc(-c4c(C)cc(OCCOCC)cc4C)ccc3)cc2)CC1 |
| CHEMBL1829158 | 6.290 | Fc1nccc(C#Cc2ccc(CCC(=O)O)cc2)c1 |
| CHEMBL4294975 | 6.284 | Clc1c2O[C@@H](COc3ccc([C@@H](C#CC)CC(=O)O)cc3)COc2ccc1 |
| CHEMBL1829160 | 6.280 | O=C(O)CCc1ccc(C#Cc2cc(OC)ncc2)cc1 |
| CHEMBL2315544 | 6.280 | Fc1ccc(C#Cc2ccc(CCC(=O)O)cc2)cc1 |
| CHEMBL4519415 | 6.271 | Fc1c(OCC(=O)O)ccc(NCc2cc(-c3c(OC)cccc3)ccc2)c1 |
| CHEMBL3601003 | 6.268 | Clc1ccc(-c2nc(COc3ccc(CCC(=O)O)cc3)on2)cc1 |
| CHEMBL4205537 | 6.260 | S(=O)(=O)(CC(=O)O)c1ccc(OCc2c(C)nc(-c3c(F)cccc3)s2)cc1 |
| CHEMBL4203471 | 6.260 | O=C(O)CC1c2c(C(O)C1)cc(OCc1cc(-c3c(C)cc(OC)cc3C)ccc1)cc2 |
| CHEMBL3600995 | 6.252 | Fc1cc(-c2occ(COc3ccc(CCC(=O)O)cc3)n2)ccc1 |
| CHEMBL3604055 | 6.252 | Clc1c2c(c(Oc3ccc(C(OCC)CC(=O)O)cc3)cc1)cccc2 |
| CHEMBL2022250 | 6.244 | O=C(O)CC1c2c(nc(OCc3cc(-c4c(C)cccc4C)ccc3)cc2)CC1 |
| CHEMBL2315249 | 6.240 | O=C(O)CCc1ccc(C#Cc2c(CO)cccc2)cc1 |
| CHEMBL3604031 | 6.237 | S(=O)(=O)(NC(=O)CC(OCC)c1ccc(OCc2c(C)cccc2)cc1)C |
| CHEMBL3805888 | 6.237 | Fc1c(NC(=O)c2sc(COc3ccc(CCC(=O)O)cc3)nn2)cccc1 |
| CHEMBL3805952 | 6.237 | Fc1c(F)ccc(NC(=O)c2sc(COc3ccc(CCC(=O)O)cc3)nn2)c1 |
| CHEMBL4528449 | 6.229 | O=C(O)CCc1ccc(OCc2c(C)nc(-c3ccccc3)s2)cc1 |
| CHEMBL1829148 | 6.220 | Clc1c(C#Cc2ccc(CCC(=O)O)cc2)cccn1 |
| CHEMBL3601004 | 6.215 | FC(F)(F)c1ccc(-c2nc(COc3ccc(CCC(=O)O)cc3)on2)cc1 |
| CHEMBL4277789 | 6.215 | O=C(O)C#Cc1ccc(OCc2cc(-c3c(C)cccc3)ccc2)cc1 |
| CHEMBL4285740 | 6.194 | Clc1cc2OC(COc3ccc(C(OCC)CC(=O)O)cc3)COc2cc1 |
| CHEMBL1829150 | 6.180 | Fc1c(C#Cc2ccc(CCC(=O)O)cc2)cc(C)cn1 |
| CHEMBL4212041 | 6.173 | S(=O)(=O)(C)c1c(-c2c(C)cccc2)c2c(s1)ccc(COc1ccc([C@@H](C#CC)Cc3[nH]nnn3)cc1)c2 |
| CHEMBL3601005 | 6.167 | Fc1ccc(-c2nc(COc3ccc(CCC(=O)O)cc3)on2)cc1 |
| CHEMBL4285071 | 6.161 | Clc1c(-c2cc(COc3ccc(C#CC(=O)O)cc3)ccc2)cccc1 |
| CHEMBL3600996 | 6.155 | O=C(O)CCc1ccc(OCc2nc(-c3ccccc3)oc2)cc1 |
| CHEMBL4286382 | 6.155 | FC(F)(F)Oc1cc2O[C@@H](COc3ccc([C@@H](C#CC)CC(=O)O)cc3)COc2cc1 |
| CHEMBL1829149 | 6.150 | Clc1ncc(C#Cc2ccc(CCC(=O)O)cc2)cc1 |
| CHEMBL4469691 | 6.138 | Fc1c(OCC(=O)O)ccc(NCc2cc(-c3c(C#N)cccc3)ccc2)c1 |
| CHEMBL3805328 | 6.119 | O=C(O)CCc1sc(C(=O)Nc2cc(-c3cc(C)ccc3)ccc2)nn1 |
| CHEMBL4163511 | 6.109 | Clc1ccc(-c2[nH]c3c(c2)cc(CCC(=O)O)cc3)cc1 |
| CHEMBL1829168 | 6.100 | Clc1c(Cc2ccccc2)c(C#Cc2ccc(CCC(=O)O)cc2)ccn1 |
| CHEMBL2315253 | 6.090 | S(=O)(=O)(CCCOc1cc(C#Cc2ccc(CCC(=O)O)cc2)ccc1)C |
| CHEMBL4281714 | 6.081 | Clc1cc2OC(COc3cc(F)c(CCC(=O)O)cc3)COc2cc1 |
| CHEMBL4218822 | 6.067 | O(Cc1cc2c(C3CC3)csc2cc1)c1ccc([C@@H](C#CC)Cc2[nH]nnn2)cc1 |
| CHEMBL3799646 | 6.064 | Fc1c(CCC(=O)O)ccc(OCc2cc(-c3c(C)onc3C)ncc2)c1 |
| CHEMBL4073421 | 6.046 | O=C(O)CCc1ccc(OCc2ccc(CN3CCC4(OCCC(Oc5ncccc5)C4)CC3)cc2)cc1 |
| CHEMBL2022245 | 6.027 | O=C(O)CC1c2c(cc(OCc3cc(-c4c(C)cccc4C)ccc3)cc2)CCCC1 |
| CHEMBL2022578 | 6.018 | O=C(O)C1Cc2c(ccc(OCc3cc(-c4c(C)cc(OCCOCC)cc4C)ccc3)c2)C1 |
| CHEMBL3604039 | 6.013 | Clc1c(Cl)ccc(COc2cc(OC)c(C(OCC)CC(=O)O)cc2)c1 |
| CHEMBL2315556 | 6.010 | O=C(O)Cc1ccc(C#Cc2ccccc2)cc1 |
| CHEMBL4278249 | 6.009 | Brc1cc2OCC(COc3cc(F)c(CCC(=O)O)cc3)Oc2cc1 |
| CHEMBL2315557 | 6.000 | O=C(O)CCCc1ccc(C#Cc2ccccc2)cc1 |
| CHEMBL2315546 | 6.000 | O=C(O)CCc1ccc(C#Cc2c(-c3ccccc3)cccc2)cc1 |
| CHEMBL4161206 | 5.993 | FC(F)(F)Oc1c(-c2[nH]c3c(c2)cc(CCC(=O)O)cc3)cccc1 |
| CHEMBL4218014 | 5.991 | Fc1c(C(Cc2sc(C(=O)Nc3ccc(CCC(=O)O)cc3)nn2)c2ccccc2)cccc1 |
| CHEMBL4277516 | 5.987 | Clc1cc2OC(COc3ccc(C(CC(=O)O)c4ocnc4)cc3)COc2cc1 |
| CHEMBL3806208 | 5.975 | O=C(O)CCc1ccc(OCc2sc(C(=O)Nc3ccccc3)nn2)cc1 |
| CHEMBL3600997 | 5.971 | Fc1cc(-c2oc(COc3ccc(CCC(=O)O)cc3)nc2)ccc1 |
| CHEMBL1829163 | 5.970 | O=C(O)CCc1ccc(C#Cc2cc(-c3c(C)cccc3)ncc2)cc1 |
| CHEMBL4285594 | 5.959 | O=C(O)C#Cc1ccc(OCc2cc(Oc3ccccc3)ccc2)cc1 |
| CHEMBL3601006 | 5.955 | FC(F)(F)c1cc(-c2nc(COc3ccc(CCC(=O)O)cc3)on2)ccc1 |
| CHEMBL1829157 | 5.950 | O=C(O)CCc1ccc(C#Cc2cc(C)ncc2)cc1 |
| CHEMBL3601007 | 5.947 | O=C(O)CCc1ccc(OCc2onc(-c3ccccc3)n2)cc1 |
| CHEMBL4212573 | 5.947 | O=C(O)CCc1ccc(NC(=O)c2sc(COc3cc(C)c(C)cc3)nn2)cc1 |
| CHEMBL3759212 | 5.939 | O(Cc1cc(-c2c(C)cccc2C)ccc1)c1ccc(CC2C(=O)NC(=O)S2)cc1 |
| CHEMBL4212664 | 5.932 | O=C(O)CCc1ccc(NC(=O)c2sc(COc3ccc(CC)cc3)nn2)cc1 |
| CHEMBL4294582 | 5.932 | Clc1cc2OC(COc3ccc(C(CC(=O)O)c4ccncc4)cc3)COc2cc1 |
| CHEMBL3805903 | 5.924 | Fc1cc(NC(=O)c2sc(COc3ccc(CCC(=O)O)cc3)nn2)ccc1 |
| CHEMBL3601008 | 5.921 | Clc1c(-c2nc(COc3ccc(CCC(=O)O)cc3)on2)cccc1 |
| CHEMBL4175260 | 5.918 | Fc1c(-c2[nH]c3c(c2)cc(CCC(=O)O)cc3)cccc1 |
| CHEMBL3601049 | 5.900 | Brc1cc(-c2nc(COc3ccc(CCC(=O)O)cc3)on2)ccc1 |
| CHEMBL3805879 | 5.893 | Fc1ccc(-c2cc(NC(=O)c3sc(CCC(=O)O)nn3)ccc2)cc1 |
| CHEMBL4171817 | 5.883 | FC(F)(F)c1c(-c2[nH]c3c(c2)cc(CCC(=O)O)cc3)cccc1 |
| CHEMBL4215600 | 5.870 | O=C(O)CCc1ccc(NC(=O)c2sc(CC(c3ccc(OC)cc3)c3ccccc3)nn2)cc1 |
| CHEMBL4454699 | 5.870 | Fc1c(OCC(=O)O)ccc(OCc2c(C)nc(-c3ccccc3)o2)c1 |
| CHEMBL4215103 | 5.860 | O=C(O)CCc1ccc(NC(=O)c2sc(COc3cc4c(cc3)CCC4)nn2)cc1 |
| CHEMBL4211335 | 5.850 | O=[SH0]1c2c(C(c3c(C)cccc3)=C1)cc(COc1ccc([C@@H](C#CC)Cc3[nH]nnn3)cc1)cc2 |
| CHEMBL3785789 | 5.840 | O=C(O)CCc1ccc(C#CCCCCCCC)cc1 |
| CHEMBL4289597 | 5.833 | Fc1c(CCC(=O)O)ccc(OCC2Oc3c(OC2)ccc(OC)c3)c1 |
| CHEMBL3604035 | 5.824 | Clc1c(Cl)ccc(COc2c(C)cc(C(OCC)CC(=O)O)cc2)c1 |
| CHEMBL3805668 | 5.818 | O=C(O)CCc1ccc(OCc2sc(C(=O)Nc3c(C)cccc3)nn2)cc1 |
| CHEMBL4203431 | 5.804 | Clc1cc(OCc2sc(C(=O)Nc3ccc(CCC(=O)O)cc3)nn2)ccc1 |
| CHEMBL3600998 | 5.796 | O=C(O)CCc1ccc(OCc2oc(-c3ccccc3)cn2)cc1 |
| CHEMBL3805303 | 5.785 | O=C(O)CCc1sc(C(=O)Nc2cc(-c3occc3)ccc2)nn1 |
| CHEMBL3758719 | 5.780 | Clc1c(O[C@@H](CCOc2ccc(CC3C(=O)NC(=O)S3)cc2)c2ccccc2)ccc(Cl)c1 |
| CHEMBL3604029 | 5.770 | O(C(Cc1[nH]nnn1)c1ccc(OCc2c(C)cccc2)cc1)CC |
| CHEMBL3806055 | 5.752 | O=C(O)CCc1ccc(OCc2sc(C(=O)Nc3cc(C)ccc3)nn2)cc1 |
| CHEMBL4171403 | 5.745 | Brc1cc(-c2[nH]c3c(c2)cc(CCC(=O)O)cc3)ccc1 |
| CHEMBL3758197 | 5.724 | O(Cc1n(CC(C)C)c2c(n1)cccc2)c1ccc(CC2C(=O)NC(=O)S2)cc1 |
| CHEMBL3797836 | 5.714 | Fc1c(CCC(=O)O)ccc(OCc2nccc(-c3c(C)onc3C)c2)c1 |
| CHEMBL2315250 | 5.690 | O=C(O)CCc1ccc(C#Cc2c(CCO)cccc2)cc1 |
| CHEMBL3604050 | 5.678 | O=C(O)CC(OCC)c1ccc(OCc2c(C)nccc2)cc1 |
| CHEMBL4207755 | 5.664 | O=C(O)CCc1ccc(NC(=O)c2sc(COc3ccc(C)cc3)nn2)cc1 |
| CHEMBL4286165 | 5.650 | Brc1cc2OC(COc3cc(F)c(CCC(=O)O)cc3)COc2cc1 |
| CHEMBL4285148 | 5.625 | Fc1c(CCC(=O)O)ccc(OCC2Oc3c(ccc(OCCOC)c3)CC2)c1 |
| CHEMBL4205508 | 5.622 | Clc1ccc(Cc2sc(C(=O)Nc3ccc(CCC(=O)O)cc3)nn2)cc1 |
| CHEMBL4208272 | 5.614 | O=C(O)CCc1ccc(NC(=O)c2sc(COc3ccc(OC)cc3)nn2)cc1 |
| CHEMBL4217530 | 5.595 | O=C(O)CCc1ccc(NC(=O)c2sc(COc3c(C)cc(C)cc3)nn2)cc1 |
| CHEMBL3601052 | 5.590 | O=C(O)CCc1ccc(OCc2onc(-c3sccc3)n2)cc1 |
| CHEMBL3601053 | 5.577 | O=C(O)CCc1ccc(OCc2nc(-c3ccccc3)on2)cc1 |
| CHEMBL4203929 | 5.567 | Clc1ccc(C(Cc2sc(C(=O)Nc3ccc(CCC(=O)O)cc3)nn2)c2ccccc2)cc1 |
| CHEMBL1829153 | 5.560 | Clc1nc(C#Cc2ccc(CCC(=O)O)cc2)cc(C)n1 |
| CHEMBL4288513 | 5.559 | O=C(O)CC1c2c(OC1)cc(OCCCOc1cc(-c3ccccc3)ccc1)cc2 |
| CHEMBL4292911 | 5.545 | FC(F)(F)Oc1c(-c2cc(COc3ccc(C#CC(=O)O)cc3)ccc2)cccc1 |
| CHEMBL3601055 | 5.523 | Clc1c(OCc2onc(-c3cc(OC)ccc3)n2)ccc(CCC(=O)O)c1 |
| CHEMBL4280212 | 5.520 | O=C(O)CC1c2c(OC1)cc(OCCCOc1cc(C(C)(C)C)ccc1)cc2 |
| CHEMBL3604036 | 5.509 | Clc1c(Cl)ccc(COc2c([N+](=O)[O-])cc(C(OCC)CC(=O)O)cc2)c1 |
| CHEMBL3604051 | 5.495 | O=C(O)CC(OCC)c1ccc(OCc2n(C)nc(C)c2)cc1 |
| CHEMBL4212106 | 5.487 | O=C(O)CCc1ccc(NC(=O)c2sc(COc3cc(C)ccc3)nn2)cc1 |
| CHEMBL4279744 | 5.469 | Clc1cc2O[C@@H](COc3ccc([C@H](C#CC)CC(=O)O)cc3)COc2cc1 |
| CHEMBL3758549 | 5.447 | Brc1cc(COc2ccc(CC3C(=O)NC(=O)S3)cc2)ccc1 |
| CHEMBL4215054 | 5.435 | O=C(O)CCc1ccc(NC(=O)c2sc(Cc3ccccc3)nn2)cc1 |
| CHEMBL4288438 | 5.424 | O=C(O)C#Cc1ccc(OCc2cc(-c3ccccc3)ccc2)cc1 |
| CHEMBL3604030 | 5.420 | FC(F)(C(=O)O)C(OCC)c1ccc(OCc2c(C)cccc2)cc1 |
| CHEMBL4277858 | 5.415 | O=C(O)CC1c2c(OC1)cc(OCCOc1cc(C(C)(C)C)ccc1)cc2 |
| CHEMBL1829154 | 5.410 | O=C(O)CCc1ccc(C#Cc2scnc2)cc1 |
| CHEMBL4278085 | 5.400 | O=C(O)C#Cc1ccc(OCc2cc(-c3c(C(C)C)cccc3)ccc2)cc1 |
| CHEMBL3601056 | 5.398 | O=C(O)CCc1ccc(OC(C)c2onc(-c3sccc3)n2)cc1 |
| CHEMBL3758919 | 5.386 | O(Cc1n(Cc2ccccc2)c2c(n1)cccc2)c1ccc(CC2C(=O)NC(=O)S2)cc1 |
| CHEMBL4281316 | 5.371 | O=C(O)CC1c2c(OC1)cc(OCCCOc1ccc(-c3ccccc3)cc1)cc2 |
| CHEMBL3805246 | 5.367 | O=C(O)CCc1ccc(OCc2sc(C(=O)Nc3ccc(C)cc3)nn2)cc1 |
| CHEMBL3601057 | 5.339 | Clc1c(OCc2onc(-c3cc(C)ccc3)n2)ccc(CCC(=O)O)c1 |
| CHEMBL2022246 | 5.310 | O=C(O)Cc1c2c(oc1)cc(OCc1cc(-c3c(C)cccc3C)ccc1)cc2 |
| CHEMBL4217225 | 5.299 | Fc1cc(Cc2sc(C(=O)Nc3ccc(CCC(=O)O)cc3)nn2)ccc1 |
| CHEMBL4202901 | 5.268 | Fc1ccc(OCc2sc(C(=O)Nc3ccc(CCC(=O)O)cc3)nn2)cc1 |
| CHEMBL3604059 | 5.252 | O=C(O)C[C@H](OCC)c1ccc(OC2c3c(nccc3)CC2)cc1 |
| CHEMBL4287775 | 5.231 | Clc1cc2OC(COc3ccc(C(CC(=O)O)c4n(C)ccn4)cc3)COc2cc1 |
| CHEMBL1829144 | 5.220 | O=C(O)CCc1ccc(C#Cc2cnccc2)cc1 |
| CHEMBL3758553 | 5.214 | FC(F)(F)c1ccc(-c2cc(COc3ccc(CC4C(=O)NC(=O)S4)cc3)ccc2)cc1 |
| CHEMBL1829146 | 5.210 | O=C(O)CCc1ccc(C#Cc2nc(C)ccc2)cc1 |
| CHEMBL316370 | 5.183 | O(Cc1ccccc1)c1ccc(CC2C(=O)NC(=O)S2)cc1 |
| CHEMBL3805086 | 5.178 | Fc1ccc(NC(=O)c2sc(COc3ccc(CCC(=O)O)cc3)nn2)cc1 |
| CHEMBL3759746 | 5.130 | FC(F)(F)c1ccc(O[C@@H](CCOc2ccc(CC3C(=O)NC(=O)S3)cc2)c2ccccc2)cc1 |
| CHEMBL4281988 | 5.118 | O=C(O)CC1c2c(OC1)cc(OCCCOc1ccc(C)cc1)cc2 |
| CHEMBL4283988 | 5.094 | Clc1cc2OC(COc3ccc(C4(CC(=O)O)COC4)cc3)COc2cc1 |
| CHEMBL4291766 | 5.093 | Clc1c(-c2cc(COc3ccc(C#CC(=O)O)cc3)ccc2)ccc(C)c1 |
| CHEMBL4295200 | 5.073 | Clc1cc(OCCCOc2cc(F)c(OCC(=O)O)cc2)ccc1 |
| CHEMBL3759913 | 5.070 | Clc1ccc(O[C@@H](CCOc2ccc(CC3C(=O)NC(=O)S3)cc2)c2ccccc2)cc1 |
| CHEMBL1829151 | 5.040 | Clc1nc(C#Cc2ccc(CCC(=O)O)cc2)ccn1 |
| CHEMBL2315533 | 5.030 | O=C(O)/C=C/c1ccc(C#Cc2ccccc2)cc1 |
| CHEMBL1829152 | 5.020 | O=C(O)CCc1ccc(C#Cc2nc(OC)ncc2)cc1 |
| CHEMBL3759467 | 5.004 | O(Cc1cc(-c2ccccc2)ccc1)c1ccc(CC2C(=O)NC(=O)S2)cc1 |
| CHEMBL3805377 | 4.974 | Clc1cc(NC(=O)c2sc(COc3ccc(CCC(=O)O)cc3)nn2)ccc1 |
| CHEMBL1829147 | 4.960 | O=[N+]([O-])c1c(C#Cc2ccc(CCC(=O)O)cc2)nccc1 |
| CHEMBL121 | 4.940 | O(CCN(C)c1ncccc1)c1ccc(CC2C(=O)NC(=O)S2)cc1 |
| CHEMBL3601058 | 4.883 | Clc1c(OCc2onc(-c3ccccc3)n2)ccc(CCC(=O)O)c1 |
| CHEMBL1829143 | 4.880 | O=C(O)CCc1ccc(C#Cc2ncccc2)cc1 |
| CHEMBL4282779 | 4.867 | Clc1cc2OC(CNc3cc(F)c(CCC(=O)O)cc3)COc2cc1 |
| CHEMBL2315550 | 4.790 | O=C(O)CCc1ccc(C#Cc2c(O)cccc2)cc1 |
| CHEMBL3759448 | 4.780 | O(Cc1n(C(C)C)c2c(n1)cccc2)c1ccc(CC2C(=O)NC(=O)S2)cc1 |
| CHEMBL3804883 | 4.746 | O=C(O)CCc1sc(C(=O)Nc2ccc(OCc3ccccc3)cc2)nn1 |
| CHEMBL3601061 | 4.726 | Clc1cc(-c2nc(COc3ccc(CC(=O)O)cc3)on2)ccc1 |
| CHEMBL3759866 | 4.708 | O([C@@H](CCOc1ccc(CC2C(=O)NC(=O)S2)cc1)c1ccccc1)c1cc(OC)ccc1 |
| CHEMBL3758885 | 4.678 | O(Cc1n(CC=C)c2c(n1)cccc2)c1ccc(CC2C(=O)NC(=O)S2)cc1 |
| CHEMBL3759133 | 4.636 | Brc1ccc(COc2ccc(CC3C(=O)NC(=O)S3)cc2)cc1 |
| CHEMBL4283015 | 4.565 | S(=O)(=O)(C)c1cc2O[C@@H](COc3ccc([C@@H](C#CC)CC(=O)O)cc3)COc2cc1 |
| CHEMBL3601059 | 4.521 | Clc1c(OCc2onc(-c3cc(C(F)(F)F)ccc3)n2)ccc(CCC(=O)O)c1 |
| CHEMBL4278799 | 4.440 | S(=O)(=O)(CCCOc1cc2OC(COc3cc(F)c(CCC(=O)O)cc3)CCc2cc1)C |
| CHEMBL3600999 | 4.323 | Clc1cc(-c2oc(COc3ccc(CCC(=O)O)cc3)nc2)ccc1 |
| CHEMBL595 | 3.090 | O(CCc1ncc(CC)cc1)c1ccc(CC2C(=O)NC(=O)S2)cc1 |

## Table S2. Description of 153 MOE two-dimensional descriptors.

| **Code** | **Description** |
| --- | --- |
| apol | Sum of the atomic polarizabilities (including implicit hydrogens) with polarizabilities taken from [CRC 1994]. |
| a_acc | Number of hydrogen bond acceptor atoms (not counting acidic atoms but counting atoms that are both hydrogen bond donors and acceptors such as -OH). |
| a_acid | Number of acidic atoms. |
| a_aro | Number of aromatic atoms. |
| a_base | Number of basic atoms. |
| a_count | Number of atoms (including implicit hydrogens). This is calculated as the sum of (1 + hi) over all non-trivial atoms i. |
| a_don | Number of hydrogen bond donor atoms (not counting basic atoms but counting atoms that are both hydrogen bond donors and acceptors such as -OH). |
| a_donacc | Number of hydrogen bond donor plus number of hydrogen bond acceptor atoms. |
| a_heavy | Number of heavy atoms #{Zi \| Zi > 1}. |
| a_hyd | Number of hydrophobic atoms. |
| a_IC | Atom information content (total). This is calculated to be a_ICM times n. |
| a_ICM | Atom information content (mean). This is the entropy of the element distribution in the molecule (including implicit hydrogens but not lone pair pseudo-atoms). Let ni be the number of occurrences of atomic number i in the molecule. Let pi = ni / n where n is the sum of the ni. The value of a_ICM is the negative of the sum over all i of pi log pi. |
| a_nC | Number of carbon atoms: #{Zi \| Zi = 6}. |
| a_nH | Number of hydrogen atoms (including implicit hydrogens). This is calculated as the sum of hi over all non-trivial atoms i plus the number of non-trivial hydrogen atoms. |
| a_nI | Number of iodine atoms: #{Zi \| Zi = 53}. |
| a_nO | Number of oxygen atoms: #{Zi \| Zi = 8}. |
| a_nS | Number of sulfur atoms: #{Zi \| Zi = 16}. |
| balabanJ | Balaban's connectivity topological index [Balaban 1982]. |
| BCUT_PEOE_0 | The BCUT descriptors [Pearlman 1998] are calculated from the eigenvalues of a modified adjacency matrix. Each ij entry of the adjacency matrix takes the value 1/sqrt(bij) where bij is the formal bond order between bonded atoms i and j. The diagonal takes the value of the PEOE partial charges. The resulting eigenvalues are sorted and the smallest, 1/3-ile, 2/3-ile and largest eigenvalues are reported. |
| BCUT_PEOE_1 |  |
| BCUT_PEOE_2 |  |
| BCUT_PEOE_3 |  |
| BCUT_SLOGP_0 | The BCUT descriptors using atomic contribution to logP (using the Wildman and Crippen SlogP method) instead of partial charge. |
| BCUT_SLOGP_1 |  |
| BCUT_SLOGP_2 |  |
| BCUT_SLOGP_3 |  |
| BCUT_SMR_0 | The BCUT descriptors using atomic contribution to molar refractivity (using the Wildman and Crippen SMR method) instead of partial charge.diameterLargest value in the distance matrix [Petitjean 1992].petitjeanValue of (diameter - radius) / diameter. |
| BCUT_SMR_1 |  |
| BCUT_SMR_2 |  |
| BCUT_SMR_3 |  |
| bpol | Sum of the absolute value of the difference between atomic polarizabilities of all bonded atoms in the molecule (including implicit hydrogens) with polarizabilities taken from [CRC 1994]. |
| b_1rotN | Number of rotatable single bonds. Conjugated single bonds are not included (e.g. ester and peptide bonds). |
| b_1rotR | Fraction of rotatable single bonds: b_1rotN divided by b_heavy. |
| b_ar | Number of aromatic bonds. |
| b_count | Number of bonds (including implicit hydrogens). This is calculated as the sum of (di/2 + hi) over all non-trivial atoms i. |
| b_double | Number of double bonds. Aromatic bonds are not considered to be double bonds. |
| b_heavy | Number of bonds between heavy atoms. |
| b_max1len | Length of the longest single bond chain. |
| b_rotN | Number of rotatable bonds. A bond is rotatable if it has order 1, is not in a ring, and has at least two heavy neighbors. |
| b_rotR | Fraction of rotatable bonds: b_rotN divided by b_heavy. |
| b_single | Number of single bonds (including implicit hydrogens). Aromatic bonds are not considered to be single bonds. |
| chi0 | Atomic connectivity index (order 0) from [Hall 1991] and [Hall 1977]. This is calculated as the sum of 1/sqrt(di) over all heavy atoms i with di > 0. |
| chi0v | Atomic valence connectivity index (order 0) from [Hall 1991] and [Hall 1977]. This is calculated as the sum of 1/sqrt(vi) over all heavy atoms i with vi > 0. |
| chi0v_C | Carbon valence connectivity index (order 0). This is calculated as the sum of 1/sqrt(vi) over all carbon atoms i with vi > 0. |
| chi0_C | Carbon connectivity index (order 0). This is calculated as the sum of 1/sqrt(di) over all carbon atoms i with di > 0. |
| chi1 | Atomic connectivity index (order 1) from [Hall 1991] and [Hall 1977]. This is calculated as the sum of 1/sqrt(didj) over all bonds between heavy atoms i and j where i < j. |
| chi1v | Atomic valence connectivity index (order 1) from [Hall 1991] and [Hall 1977]. This is calculated as the sum of 1/sqrt(vivj) over all bonds between heavy atoms i and j where i < j. |
| chi1v_C | Carbon valence connectivity index (order 1). This is calculated as the sum of 1/sqrt(vivj) over all bonds between carbon atoms i and j where i < j. |
| chi1_C | Carbon connectivity index (order 1). This is calculated as the sum of 1/sqrt(didj) over all bonds between carbon atoms i and j where i < j. |
| chiral | The number of chiral centers. |
| chiral_u | The number of unconstrained chiral centers. |
| density | Molecular mass density: Weight divided by vdw_vol (amu/Å3). |
| diameter | Largest value in the distance matrix [Petitjean 1992]. |
| FCharge | Total charge of the molecule (sum of formal charges). |
| GCUT_SMR_0 | The GCUT descriptors using atomic contribution to molar refractivity (using the Wildman and Crippen SMR method) instead of partial charge. |
| GCUT_SMR_1 |  |
| GCUT_SMR_2 |  |
| GCUT_SMR_3 |  |
| h_ema | Sum of hydrogen bond acceptor strengths [Gerber 1998]. |
| h_emd | Sum of hydrogen bond donor strengths [Gerber 1998]. |
| h_emd_C | Sum of hydrogen bond donor strengths of carbon atoms. |
| h_logP | Log of the octanol/water partition coefficient using an 8 parameter model based on Hueckel Theory [Labute 2015] with r2 = 0.84, RMSE=0.59 on 1,836 molecules. The calculated value is that of the protonation state of the structure as drawn. |
| h_logS | Log of the aqueous solubility (mol/L) using a 7 parameter model based on Hueckel Theory [Labute 2015] with r2 = 0.83, RMSE=0.85 on 1,708 molecules. The calculated value is that of the protonation state of the structure as drawn. |
| h_log_pbo | Sum of log (1 + pi bond order) for all bonds. |
| h_mr | Molar refractivity using a 4 parameter model based on Hueckel Theory [Labute 2015] with r2 = 0.99, RMSE=0.20 on 1,947 molecules. |
| Kier1 | First kappa shape index: (n-1)2 / m2 [Hall 1991]. |
| Kier2 | Second kappa shape index: (n-1)2 / m2 [Hall 1991]. |
| Kier3 | Third kappa shape index: (n-1) (n-3)2 / p32 for odd n, and (n-3) (n-2)2 / p32 for even n [Hall 1991]. |
| KierA1 | First alpha modified shape index: s (s-1)2 / m2 where s = n + a [Hall 1991]. |
| KierA2 | Second alpha modified shape index: s (s-1)2 / m2 where s = n + a [Hall 1991]. |
| KierA3 | Third alpha modified shape index: (s-1) (s-3)2 / p32 for odd n, and (s-3) (s-2)2 / p32 for even n where s = n + a [Hall 1991]. |
| KierFlex | Kier molecular flexibility index: (KierA1) (KierA2) / n [Hall 1991]. |
| lip_acc | The number of O and N atoms. |
| lip_don | The number of OH and NH atoms. |
| logP(o/w) | Log of the octanol/water partition coefficient (including implicit hydrogens). This property is calculated from a ~100 parameter linear atom type model [LOGP 1998] with r2 = 0.931, RMSE=0.393 on 1,827 molecules. |
| logS | Log of the aqueous solubility (mol/L). This property is calculated from an atom contribution linear atom type model [Hou 2004] with r2 = 0.90, ~1,200 molecules. |
| mr | Molecular refractivity (including implicit hydrogens). This property is calculated from an 11 descriptor linear model [MREF 1998] with r2 = 0.997, RMSE = 0.168 on 1,947 small molecules. |
| opr_brigid | The number of rigid bonds from [Oprea 2000]. |
| opr_nring | The number of rings from [Oprea 2000]. |
| opr_nrot | The number of rotatable bonds from [Oprea 2000]. |
| opr_violation | The number of violations of Oprea's lead-like test [Oprea 2000]. |
| PEOE_PC+ | Total positive partial charge: the sum of the positive qi. Q_PC+ is identical to PC+ which has been retained for compatibility. |
| PEOE_PC- | Total negative partial charge: the sum of the negative qi. Q_PC- is identical to PC- which has been retained for compatibility. |
| PEOE_RPC+ | Relative positive partial charge: the largest positive qi divided by the sum of the positive qi. Q_RPC+ is identical to RPC+ which has been retained for compatibility. |
| PEOE_RPC- | Relative negative partial charge: the smallest negative qi divided by the sum of the negative qi. Q_RPC- is identical to RPC- which has been retained for compatibility. |
| PEOE_VSA+0 | Sum of vi where qi is in the range [0.00,0.05). |
| PEOE_VSA+1 | Sum of vi where qi is in the range [0.05,0.10). |
| PEOE_VSA+2 | Sum of vi where qi is in the range [0.10,0.15). |
| PEOE_VSA+3 | Sum of vi where qi is in the range [0.15,0.20). |
| PEOE_VSA+4 | Sum of vi where qi is in the range [0.20,0.25). |
| PEOE_VSA+5 | Sum of vi where qi is in the range [0.25,0.30). |
| PEOE_VSA+6 | Sum of vi where qi is greater than 0.3. |
| PEOE_VSA-0 | Sum of vi where qi is in the range [-0.05,0.00). |
| PEOE_VSA-1 | Sum of vi where qi is in the range [-0.10,-0.05). |
| PEOE_VSA-3 | Sum of vi where qi is in the range [-0.20,-0.15). |
| PEOE_VSA-4 | Sum of vi where qi is in the range [-0.25,-0.20). |
| PEOE_VSA-5 | Sum of vi where qi is in the range [-0.30,-0.25). |
| PEOE_VSA-6 | Sum of vi where qi is less than -0.30. |
| PEOE_VSA_FHYD | Fractional hydrophobic van der Waals surface area. This is the sum of the vi such that \|qi\| is less than or equal to 0.2 divided by the total surface area. The vi are calculated using a connection table approximation. |
| PEOE_VSA_FNEG | Fractional negative van der Waals surface area. This is the sum of the vi such that qi is negative divided by the total surface area. The vi are calculated using a connection table approximation. |
| PEOE_VSA_FPNEG | Fractional negative polar van der Waals surface area. This is the sum of the vi such that qi is less than -0.2 divided by the total surface area. The vi are calculated using a connection table approximation. |
| PEOE_VSA_FPOL | Fractional polar van der Waals surface area. This is the sum of the vi such that \|qi\| is greater than 0.2 divided by the total surface area. The vi are calculated using a connection table approximation. |
| PEOE_VSA_FPOS | Fractional positive van der Waals surface area. This is the sum of the vi such that qi is non-negative divided by the total surface area. The vi are calculated using a connection table approximation. |
| PEOE_VSA_FPPOS | Fractional positive polar van der Waals surface area. This is the sum of the vi such that qi is greater than 0.2 divided by the total surface area. The vi are calculated using a connection table approximation. |
| PEOE_VSA_HYD | Total hydrophobic van der Waals surface area. This is the sum of the vi such that \|qi\| is less than or equal to 0.2. The vi are calculated using a connection table approximation. |
| PEOE_VSA_NEG | Total negative van der Waals surface area. This is the sum of the vi such that qi is negative. The vi are calculated using a connection table approximation. |
| PEOE_VSA_PNEG | Total negative polar van der Waals surface area. This is the sum of the vi such that qi is less than -0.2. The vi are calculated using a connection table approximation. |
| PEOE_VSA_POL | Total polar van der Waals surface area. This is the sum of the vi such that \|qi\| is greater than 0.2. The vi are calculated using a connection table approximation. |
| PEOE_VSA_POS | Total positive van der Waals surface area. This is the sum of the vi such that qi is non-negative. The vi are calculated using a connection table approximation. |
| PEOE_VSA_PPOS | Total positive polar van der Waals surface area. This is the sum of the vi such that qi is greater than 0.2. The vi are calculated using a connection table approximation. |
| petitjean | Value of (diameter - radius) / diameter. |
| petitjeanSC | Petitjean graph Shape Coefficient as defined in [Petitjean 1992]: (diameter - radius) / radius. |
| radius | If ri is the largest matrix entry in row i of the distance matrix D, then the radius is defined as the smallest of the ri [Petitjean 1992]. |
| reactive | Indicator of the presence of reactive groups. A non-zero value indicates that the molecule contains a reactive group. The table of reactive groups is based on the Oprea set [Oprea 2000] and includes metals, phospho-, N/O/S-N/O/S single bonds, thiols, acyl halides, Michael Acceptors, azides, esters, etc. |
| rings | The number of rings. |
| rsynth | A value in [0,1] indicating the synthetic reasonableness, or feasibility, of the chemical structure. A value of 0 means it is unlikely that the molecule can be synthesized while a value of 1 means that it is likely that the molecule can be synthesized. The value reflects the fraction of heavy atoms in the molecule that can be traced back to starting materials fragments resulting from retrosynthetic disconnection rules. This molecular descriptor can also be calculated on molecules and databases using the SVL Retrosynth* function calls. |
| SlogP | Log of the octanol/water partition coefficient. This property is an atomic contribution model [Crippen 1999] that calculates logP from the given structure; i.e. the correct protonation state (washed structures). Results may vary from the logP(o/w) descriptor. The training set for SlogP was ~7000 structures. |
| SlogP_VSA0 | Sum of vi such that Li <= -0.4. |
| SlogP_VSA1 | Sum of vi such that Li is in (-0.4,-0.2]. |
| SlogP_VSA2 | Sum of vi such that Li is in (-0.2,0]. |
| SlogP_VSA3 | Sum of vi such that Li is in (0,0.1]. |
| SlogP_VSA4 | Sum of vi such that Li is in (0.1,0.15]. |
| SlogP_VSA5 | Sum of vi such that Li is in (0.15,0.20]. |
| SlogP_VSA7 | Sum of vi such that Li is in (0.25,0.30]. |
| SlogP_VSA8 | Sum of vi such that Li is in (0.30,0.40]. |
| SlogP_VSA9 | Sum of vi such that Li > 0.40. |
| SMR | Molecular refractivity (including implicit hydrogens). This property is an atomic contribution model [Crippen 1999] that assumes the correct protonation state (washed structures). The model was trained on ~7000 structures and results may vary from the mr descriptor. |
| SMR_VSA0 | Sum of vi such that Ri is in [0,0.11]. |
| SMR_VSA1 | Sum of vi such that Ri is in (0.11,0.26]. |
| SMR_VSA2 | Sum of vi such that Ri is in (0.26,0.35]. |
| SMR_VSA3 | Sum of vi such that Ri is in (0.35,0.39]. |
| SMR_VSA4 | Sum of vi such that Ri is in (0.39,0.44]. |
| SMR_VSA5 | Sum of vi such that Ri is in (0.44,0.485]. |
| SMR_VSA6 | Sum of vi such that Ri is in (0.485,0.56]. |
| SMR_VSA7 | Sum of vi such that Ri > 0.56. |
| TPSA | Polar surface area (Å2) calculated using group contributions to approximate the polar surface area from connection table information only. The parameterization is that of Ertl et al. [Ertl 2000]. |
| VAdjEq | Vertex adjacency information (equality): -(1-f)log2(1-f) - f log2 f where f = (n2 - m) / n2, n is the number of heavy atoms and m is the number of heavy-heavy bonds. If f is not in the open interval (0,1), then 0 is returned. |
| VAdjMa | Vertex adjacency information (magnitude): 1 + log2 m where m is the number of heavy-heavy bonds. If m is zero, then zero is returned. |
| VDistEq | If m is the sum of the distance matrix entries then VdistEq is defined to be the sum of log2 m - pi log2 pi / m where pi is the number of distance matrix entries equal to i. |
| VDistMa | If m is the sum of the distance matrix entries then VDistMa is defined to be the sum of log2 m - Dij log2 Dij / m over all i and j. |
| vdw_area | Area of van der Waals surface (Å2) calculated using a connection table approximation. |
| vdw_vol | van der Waals volume (Å3) calculated using a connection table approximation. |
| vsa_acc | Approximation to the sum of VDW surface areas (Å2) of pure hydrogen bond acceptors (not counting atoms that are both hydrogen bond donors and acceptors such as -OH). |
| vsa_acid | Approximation to the sum of VDW surface areas of acidic atoms (Å2). |
| vsa_base | Approximation to the sum of VDW surface areas of basic atoms (Å2). |
| vsa_don | Approximation to the sum of VDW surface areas of pure hydrogen bond donors (not counting atoms that are both hydrogen bond donors and acceptors such as -OH) (Å2). |
| vsa_hyd | Approximation to the sum of VDW surface areas of hydrophobic atoms (Å2). |
| vsa_other | Approximation to the sum of VDW surface areas (Å2) of atoms typed as "other". |
| vsa_pol | Approximation to the sum of VDW surface areas (Å2) of polar atoms (atoms that are both hydrogen bond donors and acceptors), such as -OH. |
| Weight | Molecular weight (including implicit hydrogens) in atomic mass units with atomic weights taken from [CRC 1994]. |
| wienerPath | Wiener path number: half the sum of all the distance matrix entries as defined in [Balaban 1979] and [Wiener 1947]. |
| wienerPol | Wiener polarity number: half the sum of all the distance matrix entries with a value of 3 as defined in [Balaban 1979]. |
| zagreb | Zagreb index: the sum of di2 over all heavy atoms i. |

## Table S3. All categories of unnatural amino acids.

| **Categories** | **Description** |
| --- | --- |
| ALA Derivatives | Derivatives of alanine |
| ASN-GLN Derivatives | Derivatives of asparagine and glutamine |
| ASP-GLU Derivatives | Derivatives of aspartic acid and glutamic acid |
| Cys-Met Derivatives | Derivatives of cysteine and methionine |
| Gly Derivatives | Derivatives of glycine |
| His-Arg-Lys Derivatives | Derivatives of histidine, arginine, and lysine |
| Phe Derivatives | Derivatives of phenylalanine |
| Proline Derivatives and Alicyclic Amino Acids | Derivatives of alanine and amino acids with alicyclic rings |
| Ser-Thr Derivatives | Derivatives of serine and threonine |
| Trp Derivatives | Derivatives of tryptophan |
| Tyr Derivatives | Derivatives of tyrosine |
| Val-Ile-Leu derivatives | Derivatives of valine, isoleucine, and leucine |
| α-Methyl amino acids | α-Methyl amino acids |
| Guanidyl | Amino acids with guanidine groups |
| Nitro & Dinitrophenyl amino acids | Amino acids with nitro or dinitrophenyl |
| N-Methyl Amino Acids | N-terminal methylated amino acid |
| Phosphoamino Acids | Amino acids with phosphate groups |

## Table S4. Non-zero coefficient features after Lasso feature selection.

| **Descriptor** | **Coefficients** |
| --- | --- |
| TPSA | 1.433 |
| Kier2 | 1.412 |
| PEOE_VSA-6 | 0.940 |
| SlogP | 0.893 |
| opr_nrot | 0.745 |
| vsa_pol | 0.714 |
| BCUT_SLOGP_0 | 0.575 |
| h_emd | 0.505 |
| PEOE_VSA_FHYD | 0.479 |
| h_logS | 0.453 |
| KierFlex | 0.414 |
| chi1v_C | 0.394 |
| SlogP_VSA7 | 0.324 |
| vsa_other | 0.324 |
| a_base | 0.296 |
| lip_acc | 0.274 |
| SMR_VSA2 | 0.272 |
| PEOE_VSA+2 | 0.238 |
| balabanJ | 0.237 |
| a_ICM | 0.221 |
| b_1rotR | 0.216 |
| BCUT_SMR_0 | 0.214 |
| PEOE_VSA-4 | 0.210 |
| SMR_VSA6 | 0.208 |
| GCUT_SMR_3 | 0.207 |
| PEOE_PC+ | 0.205 |
| a_nO | 0.197 |
| b_1rotN | 0.184 |
| b_max1len | 0.182 |
| chiral_u | 0.180 |
| BCUT_PEOE_3 | 0.176 |
| BCUT_SLOGP_2 | 0.165 |
| PEOE_RPC- | 0.164 |
| GCUT_SMR_1 | 0.160 |
| a_donacc | 0.156 |
| FCharge | 0.155 |
| SlogP_VSA2 | 0.154 |
| SMR_VSA4 | 0.154 |
| chi1v | 0.152 |
| BCUT_SLOGP_1 | 0.150 |
| chiral | 0.149 |
| h_ema | 0.144 |
| VAdjEq | 0.140 |
| opr_violation | 0.136 |
| BCUT_PEOE_2 | 0.135 |
| density | 0.135 |
| PEOE_VSA-3 | 0.129 |
| a_nS | 0.124 |
| BCUT_SMR_2 | 0.123 |
| PEOE_VSA_FPNEG | 0.113 |
| SlogP_VSA5 | 0.112 |
| PEOE_VSA_PNEG | 0.111 |
| BCUT_SMR_1 | 0.109 |
| PEOE_VSA_FNEG | 0.109 |
| PEOE_VSA+3 | 0.102 |
| Kier3 | 0.096 |
| PEOE_VSA+4 | 0.091 |
| rings | 0.090 |
| vsa_base | 0.084 |
| logP(o/w) | 0.081 |
| SlogP_VSA4 | 0.080 |
| weinerPath | 0.077 |
| SlogP_VSA8 | 0.071 |
| BCUT_PEOE_0 | 0.069 |
| SMR_VSA7 | 0.062 |
| SlogP_VSA3 | 0.057 |
| PEOE_VSA+6 | 0.055 |
| h_emd_C | 0.053 |
| PEOE_VSA+5 | 0.048 |
| b_double | 0.045 |
| BCUT_PEOE_1 | 0.043 |
| PEOE_VSA_FPOL | 0.043 |
| a_nH | 0.042 |
| PEOE_VSA+0 | 0.036 |
| rsynth | 0.025 |
| GCUT_SMR_0 | 0.020 |
| PEOE_VSA_FPOS | 0.019 |
| reactive | 0.017 |
| logS | 0.011 |
| petitjean | 0.003 |
| a_don | 0.002 |
| opr_nring | 0.002 |
| GCUT_SMR_2 | 0.001 |

## Table S5. 41 amino acids with a carboxyl chain.

| **Amino Name** | **Smiles** |
| --- | --- |
| AG01 | O=C([O-])C([NH3+])CCC(=O)NO |
| AG02 | O=C([O-])[C@H]([NH3+])CCC(=O)NO |
| AM07 | O=C([O-])C([NH3+])(CC(=O)[O-])C |
| AM08 | O=C([O-])[C@]([NH3+])(CC(=O)[O-])C |
| AM09 | O=C([O-])C([NH3+])(CCC(=O)[O-])C |
| AM10 | O=C([O-])[C@]([NH3+])(CCC(=O)[O-])C |
| ASP | O=C([O-])C([NH3+])CC(=O)[O-] |
| GLU | O=C([O-])C([NH3+])CCC(=O)[O-] |
| LI01 | O=C([O-])[C@@H]([NH3+])CCCCCC(=O)[O-] |
| LI02 | O=C([O-])[C@H]([NH3+])CCCCCC(=O)[O-] |
| DE07 | O=C([O-])[C@H](C([NH3+])C(=O)[O-])C |
| DE08 | O=C([O-])[C@@H](C([NH3+])C(=O)[O-])C |
| DE09 | O=C([O-])[C@H]([NH3+])[C@@H](C(=O)[O-])C |
| DE10 | O=C([O-])[C@H]([NH3+])[C@H](C(=O)[O-])C |
| DE13 | O=C([O-])C(O)C([NH3+])C(=O)[O-] |
| DE14 | O=C([O-])[C@H](O)C([NH3+])C(=O)[O-] |
| DE15 | O=C([O-])[C@@H](O)[C@@H]([NH3+])C(=O)[O-] |
| DE16 | O=C([O-])[C@H](O)[C@@H]([NH3+])C(=O)[O-] |
| DE19 | O=C([O-])[C@@H]([NH3+])CC(C(=O)[O-])C |
| DE20 | O=C([O-])[C@@H]([NH3+])C[C@H](C(=O)[O-])C |
| DE21 | O=C([O-])[C@H]([NH3+])C[C@@H](C(=O)[O-])C |
| DE22 | O=C([O-])[C@H]([NH3+])C[C@H](C(=O)[O-])C |
| DE23 | O=C([O-])[C@@H](O)C[C@H]([NH3+])C(=O)[O-] |
| DE24 | O=C([O-])[C@H](O)C[C@H]([NH3+])C(=O)[O-] |
| DE25 | O=C([O-])[C@@H](O)C[C@@H]([NH3+])C(=O)[O-] |
| DE26 | O=C([O-])[C@H](O)C[C@@H]([NH3+])C(=O)[O-] |
| DE27 | O=C([O-])[C@@H]([NH3+])C[C@@H](C(=O)[O-])Cc1ccccc1 |
| DE28 | O=C([O-])[C@@H]([NH3+])C[C@H](C(=O)[O-])Cc1ccccc1 |
| DE29 | O=C([O-])[C@H]([NH3+])C[C@@H](C(=O)[O-])Cc1ccccc1 |
| DE30 | O=C([O-])[C@H]([NH3+])C[C@H](C(=O)[O-])Cc1ccccc1 |
| DE31 | O=C([O-])C([NH3+])(CC(=O)[O-])CC(=O)[O-] |
| DE32 | O=C([O-])C([NH3+])C(C(=O)[O-])C(=O)[O-] |
| DE33 | O=C([O-])[C@H]([NH3+])C(C(=O)[O-])C(=O)[O-] |
| DE34 | O=C([O-])C([NH3+])CC(C(=O)[O-])C(=O)[O-] |
| DE35 | O=C([O-])[C@H]([NH3+])CC(C(=O)[O-])C(=O)[O-] |
| CM17 | S(C[C@H]([NH3+])C(=O)[O-])CCC(=O)[O-] |
| CM18 | S(C[C@@H]([NH3+])C(=O)[O-])CCC(=O)[O-] |
| NM47 | O=C([O-])[C@@H]([NH2+]C)CC(=O)[O-] |
| NM48 | O=C([O-])C([NH2+]C)CC(=O)[O-] |
| NM49 | O=C([O-])[C@@H]([NH2+]C)CCC(=O)[O-] |
| NM50 | O=C([O-])C([NH2+]C)CCC(=O)[O-] |

**Figure S1.** Loss function curve of CNN.

**Figure S2.** Loss function curve of DNN.

**Figure S3.** Loss function curve of DNN trained based on Morgan fingerprint.

**Figure S4.** The GpiDAPH fingerprint model uses different score functions to evaluate the similarity score of the test set. (A) GpiDAPH_Ave, (B) GpiDAPH_Dis, (C) GpiDAPH_Max, (D) GpiDAPH_Min, (E) GpiDAPH_Must. Index 1-50 is active molecule and Index 51-100 is inactive molecule.

**Figure S5.** Seven fingerprint models use the maximum scoring function to evaluate the similarity score of the test set. (A) Bit_MACCS_Max, (B) GpiDAPH_Max, (C) MACCS_Max, (D) TAD_Max, (E) TAT_Max , (F) TGD_Max, (G) TGT_Max. Index 1-50 is active molecule and Index 51-100 is inactive molecule.

**Figure S6.** The similarity scores of the redefined test set on the (A) Bit_MACCS, (B) GpiDAPH, and (C) MACCS models. Index 1-50 is active molecule and Index 51-100 is inactive molecule.

**Figure S7.** The 5 peptides were considered as potential active peptides that were analyzed in molecular dynamics simulations. (A) 4-15, (B) 5-1, (C) 4-4, (D) DE20 mutant, (E) AG02 mutant.

**Figure S8.** The complex structures of five peptides and control at 10ns. (A) 4-15, (B) AG02 mutant, (C) DE20 mutant, (D) 4-4, (E) 5-1, (F) TAK-875.

**Figure S9.** RMSD of the ligand and the complex. (A) 4-15, (B) AG02 mutant, (C) DE20 mutant, (D) TAK-875.

**Figure S10.** MSD of the ligand and GPR40 in the complex. (A) 4-15, (B) AG02 mutant, (C) DE20 mutant, (D) TAK-875.

**Figure S11.** Gyrate of the ligand in the complex. (A) 4-15, (B) AG02 mutant, (C) DE20 mutant, (D) TAK-875.

**Figure S12.** Gyrate of the GPR40 in the complex. (A) 4-15, (B) AG02 mutant, (C) DE20 mutant, (D) TAK-875.

**Figure S13.** SASA of the ligand in the complex. (A) 4-15, (B) AG02 mutant, (C) DE20 mutant, (D) TAK-875.

**Figure S14.** SASA of the GPR40 in the complex. (A) 4-15, (B) AG02 mutant, (C) DE20 mutant, (D) TAK-875.

**Figure S15.** RMSF of the GPR40 in the complex. (A) 4-15, (B) AG02 mutant, (C) DE20 mutant, (D) TAK-875.

**Figure S16.** Total energy of the complex. (A) 4-15, (B) AG02 mutant, (C) DE20 mutant, (D) TAK-875.


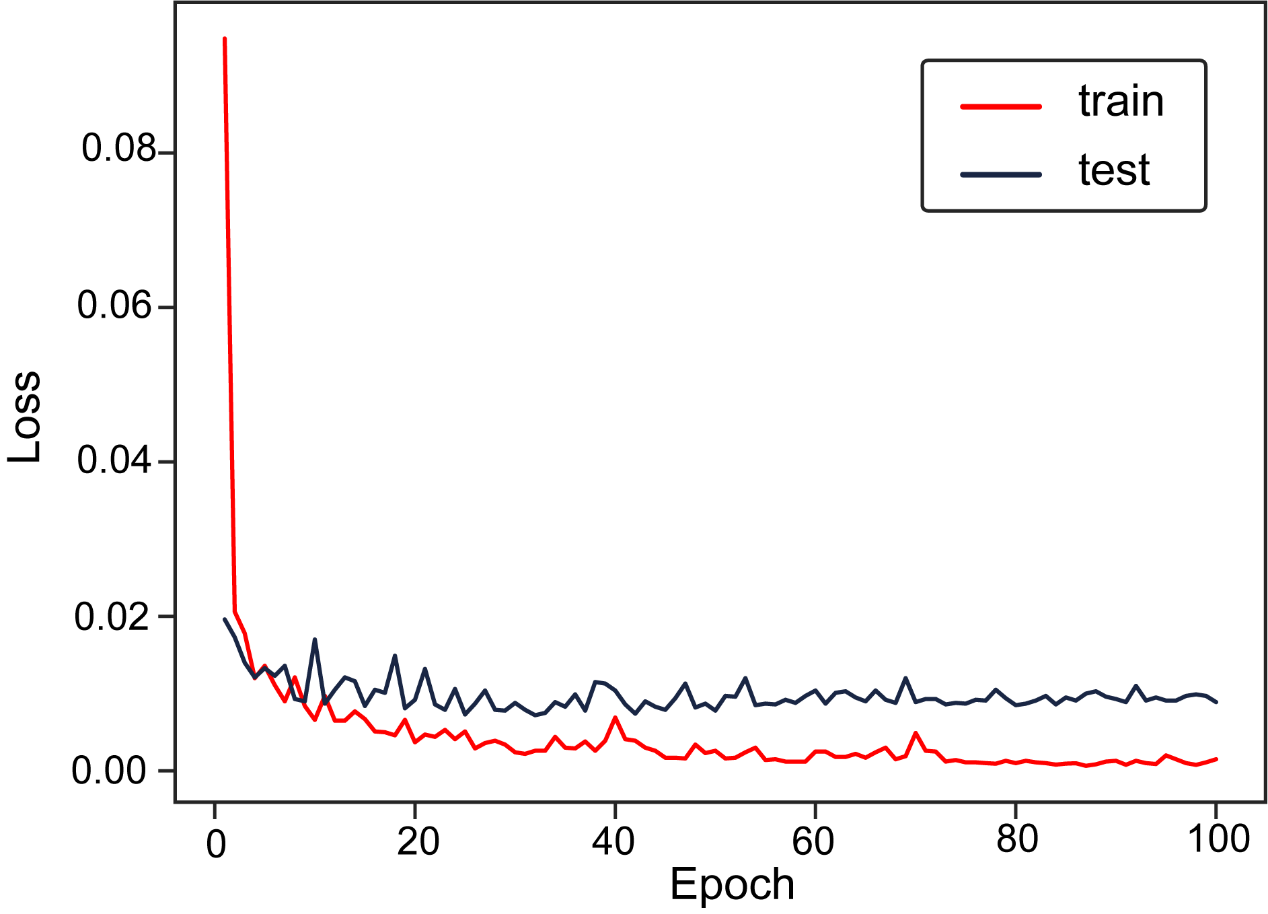


## Figure S1. Loss function curve of CNN.


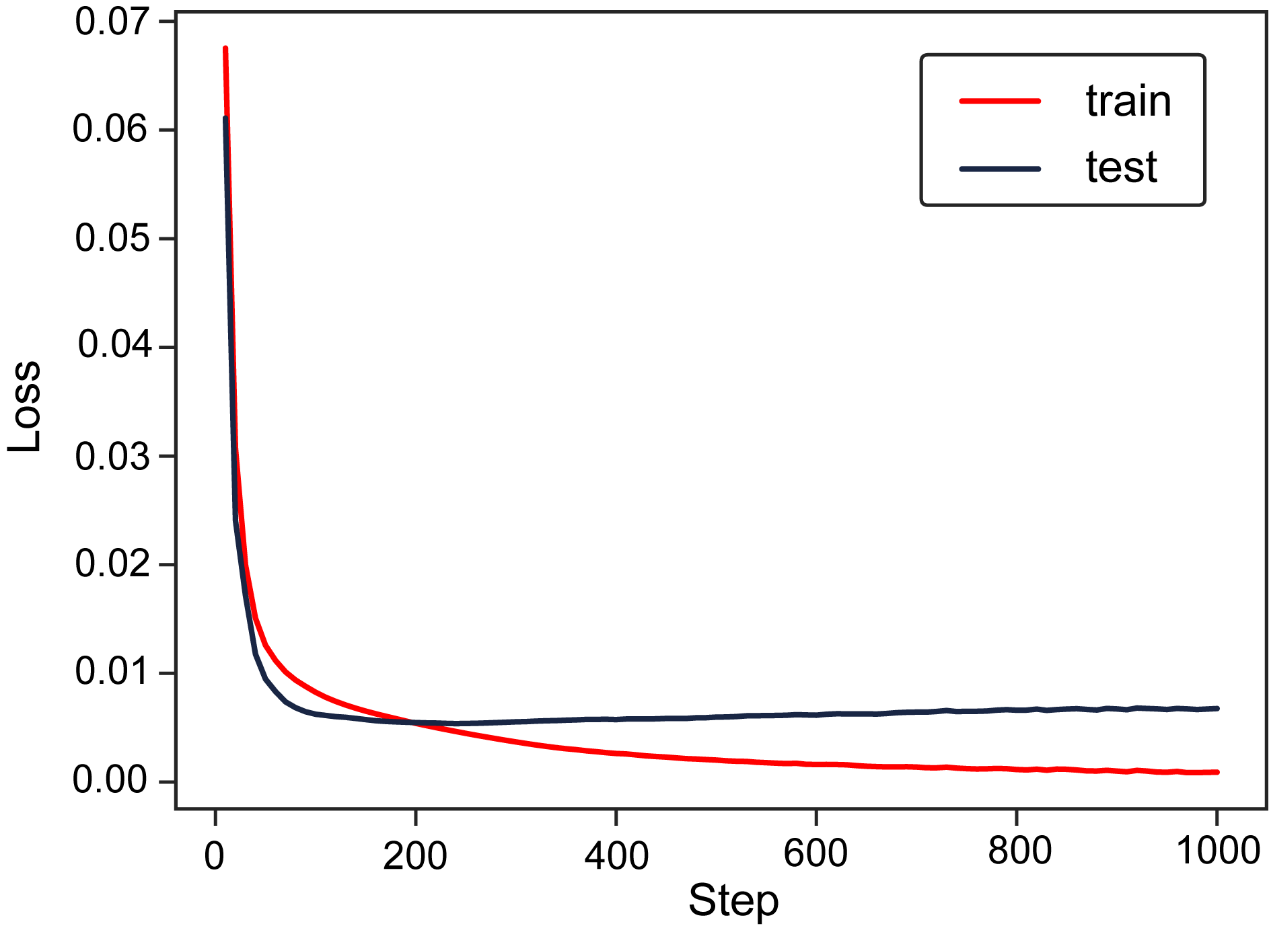


## Figure S2. Loss function curve of DNN.


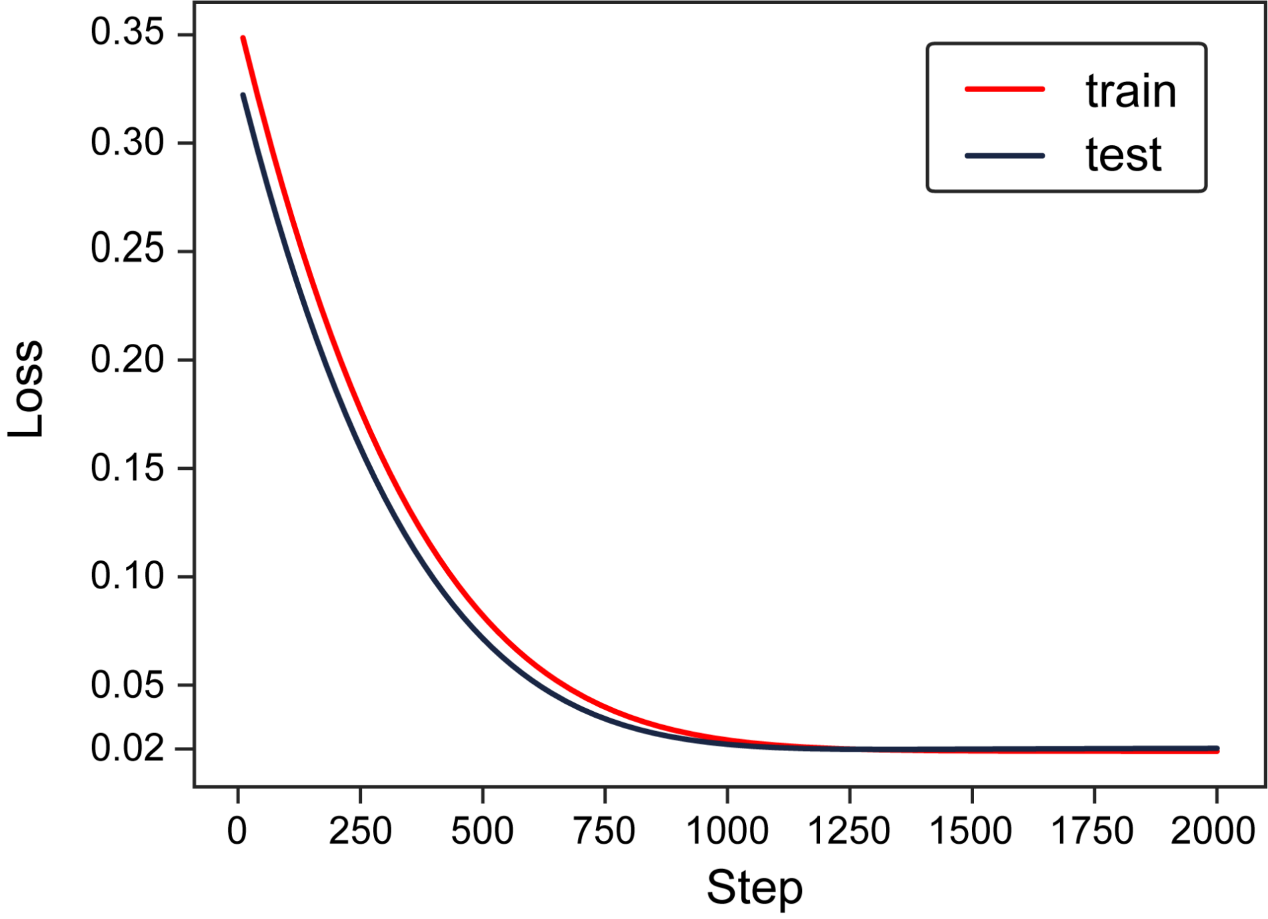


## Figure S3. Loss function curve of DNN trained based on Morgan fingerprint.

| **(A)** | **(B)** |
| --- | --- |
| 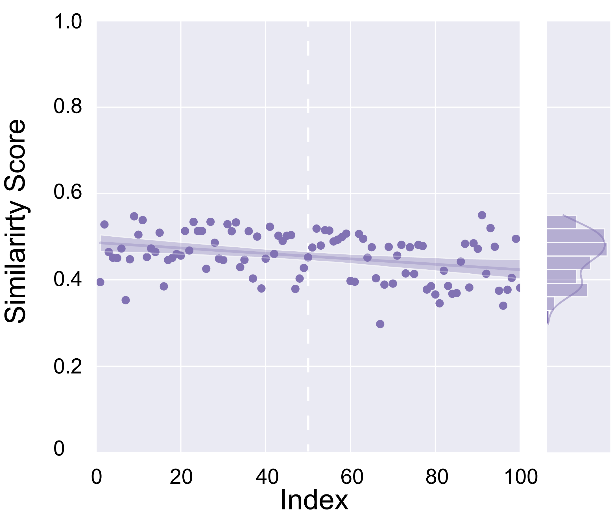 | 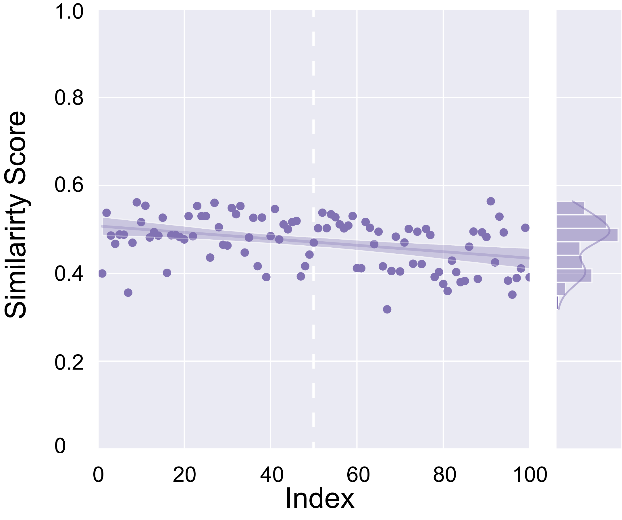 |
| **(C)** | **(D)** |
| 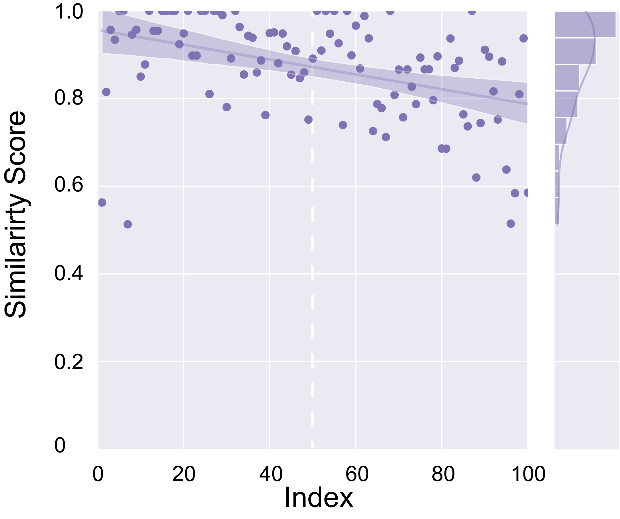 | 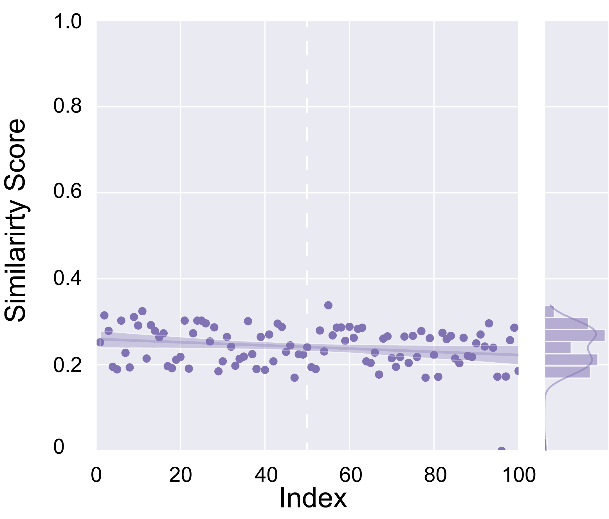 |
| **(E)** |  |
| 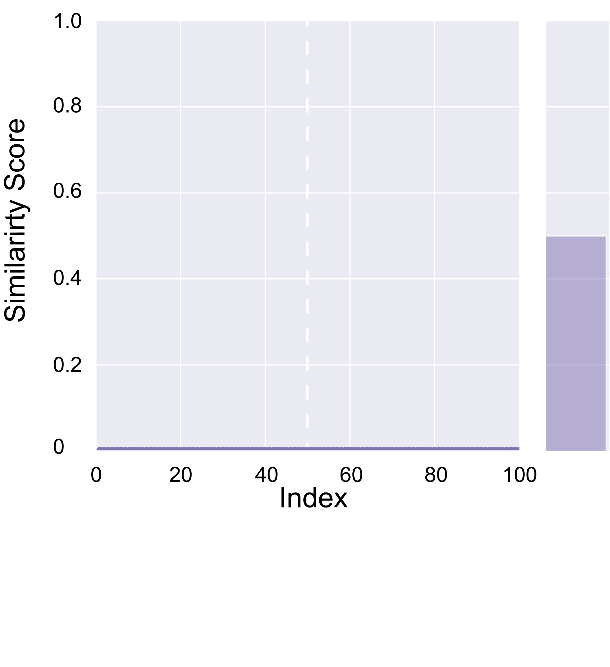 |  |

## Figure S4. The GpiDAPH fingerprint model uses different score functions to evaluate the similarity score of the test set. (A) GpiDAPH_Ave, (B) GpiDAPH_Dis, (C) GpiDAPH_Max, (D) GpiDAPH_Min, (E) GpiDAPH_Must. Index 1-50 is active molecule and Index 51-100 is inactive molecule.

| **(A)** | **(B)** |
| --- | --- |
| 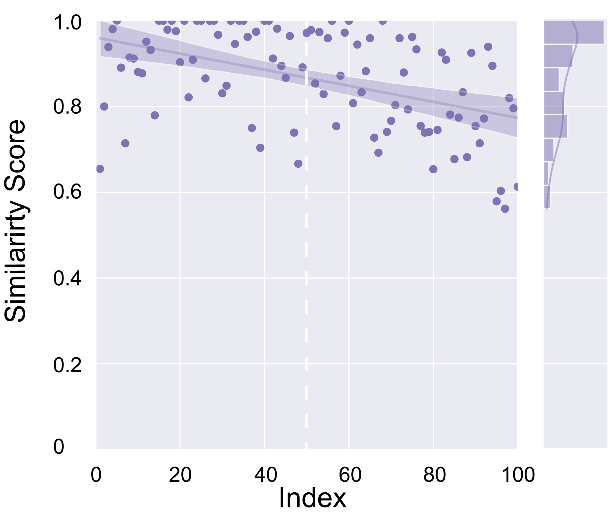 | 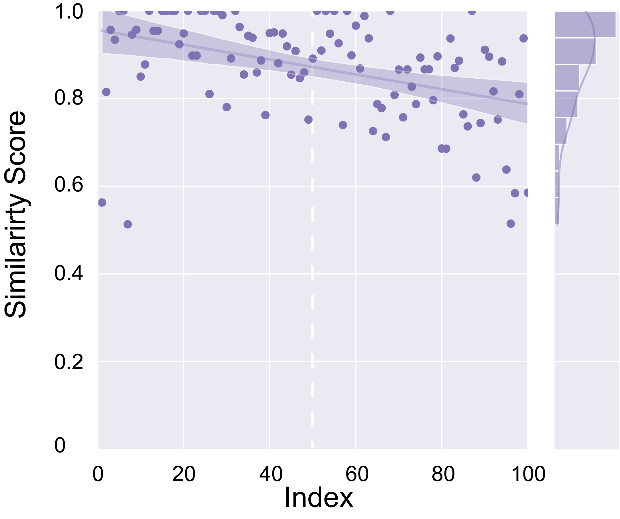 |
| **(C)** | **(D)** |
| 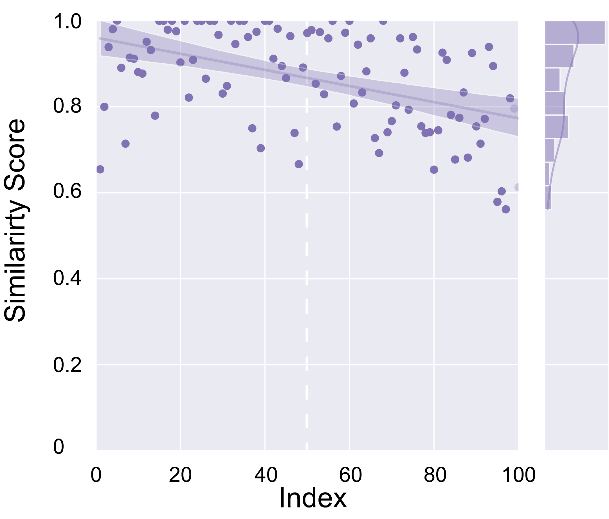 | 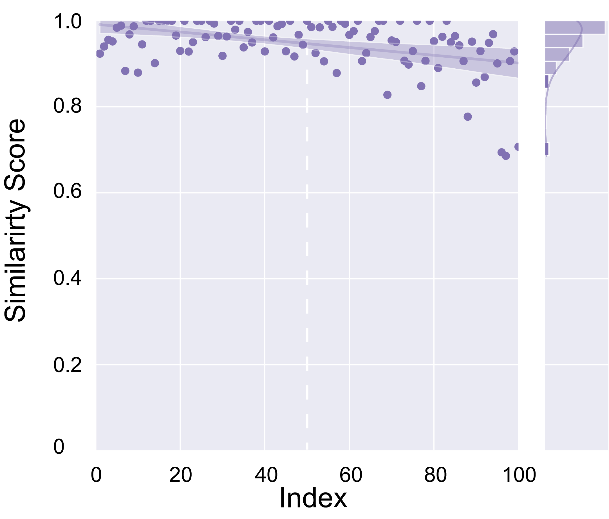 |
| **(E)** | **(F)** |
| 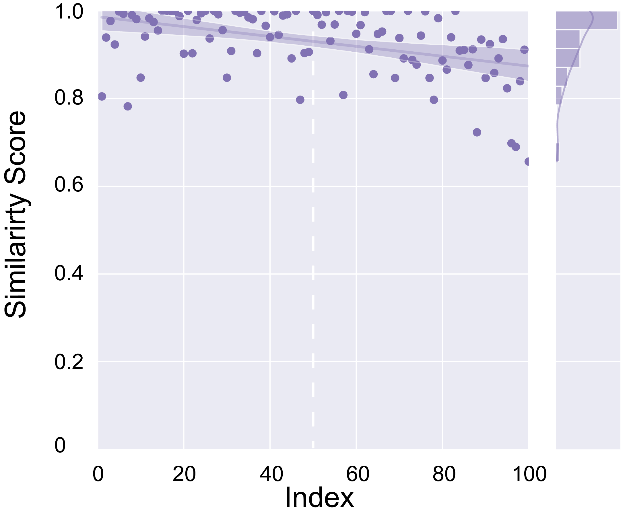 | 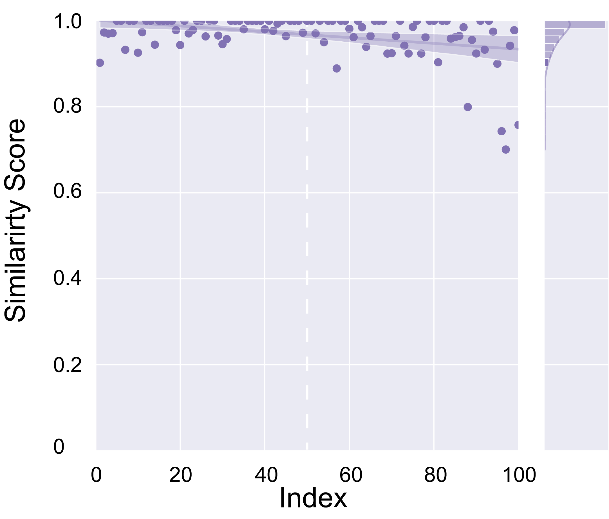 |
| **(G)** |  |
| 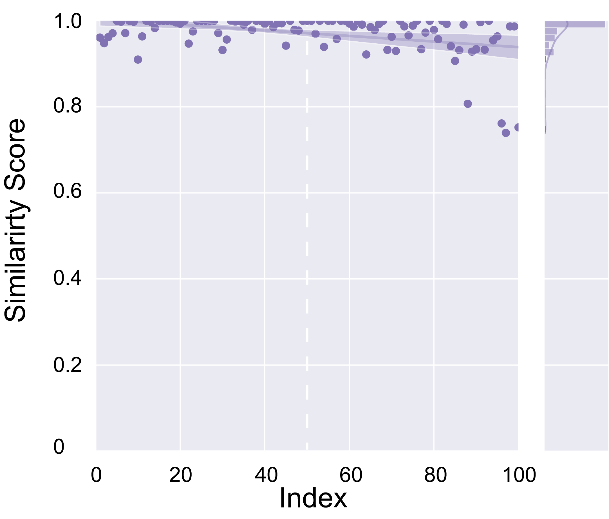 |  |

## Figure S5. Seven fingerprint models use the maximum scoring function to evaluate the similarity score of the test set. (A) Bit_MACCS_Max, (B) GpiDAPH_Max, (C) MACCS_Max, (D) TAD_Max, (E) TAT_Max , (F) TGD_Max, (G) TGT_Max. Index 1-50 is active molecule and Index 51-100 is inactive molecule.

| **(A)** | **(B)** |
| --- | --- |
| 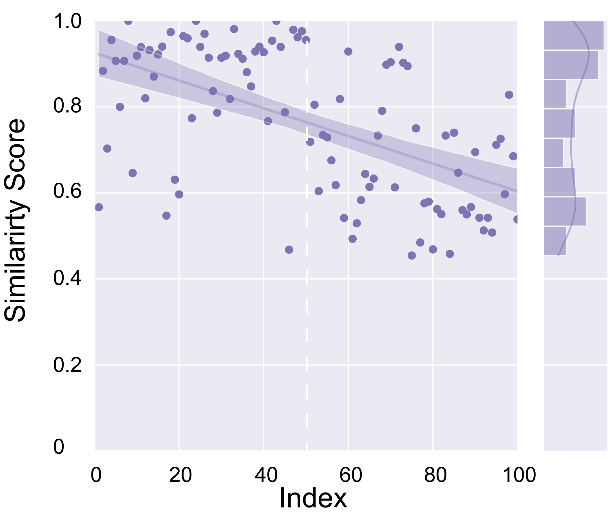 | 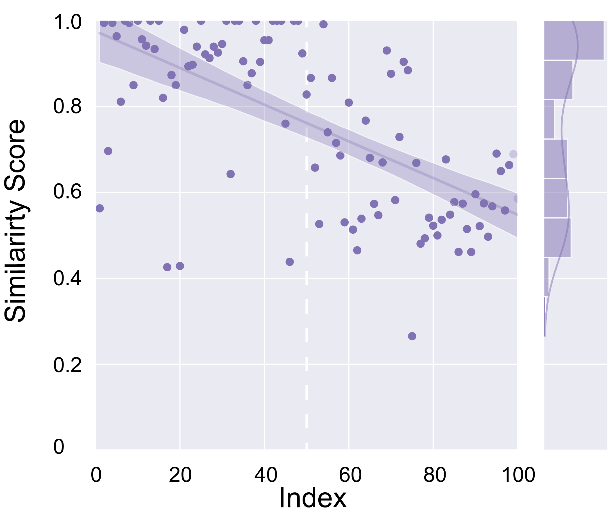 |
| **(C)** |  |
| 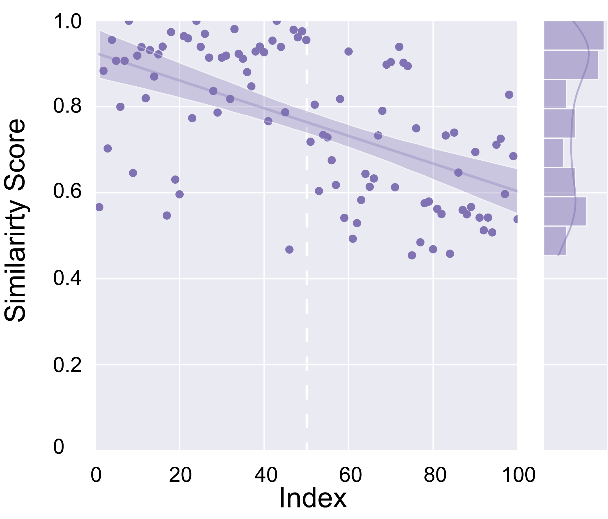 |  |

## Figure S6. The similarity scores of the redefined test set on the (A) Bit_MACCS, (B) GpiDAPH, and (C) MACCS models. Index 1-50 is active molecule and Index 51-100 is inactive molecule.

| **(A)** | **(B)** |
| --- | --- |
|  |  |
| **(C)** | **(D)** |
|  |  |
| **(E)** |  |
|  |  |

## Figure S7. The 5 peptides were considered as potential active peptides that were analyzed in molecular dynamics simulations. (A) 4-15, (B) 5-1, (C) 4-4, (D) DE20 mutant, (E) AG02 mutant.


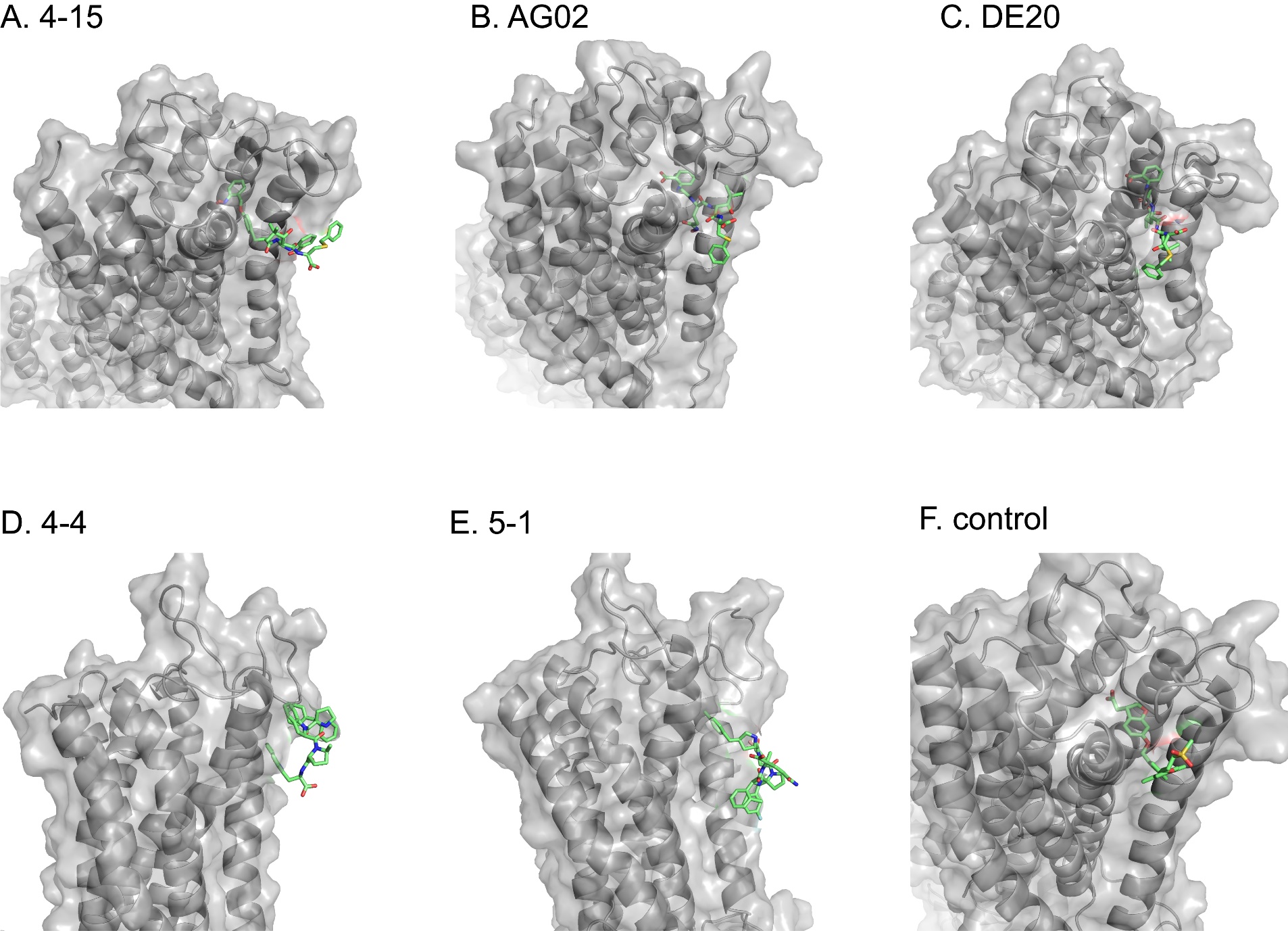


## Figure S8. The complex structures of five peptides and control at 10ns. (A) 4-15, (B) AG02 mutant, (C) DE20 mutant, (D) 4-4, (E) 5-1, (F) TAK-875.


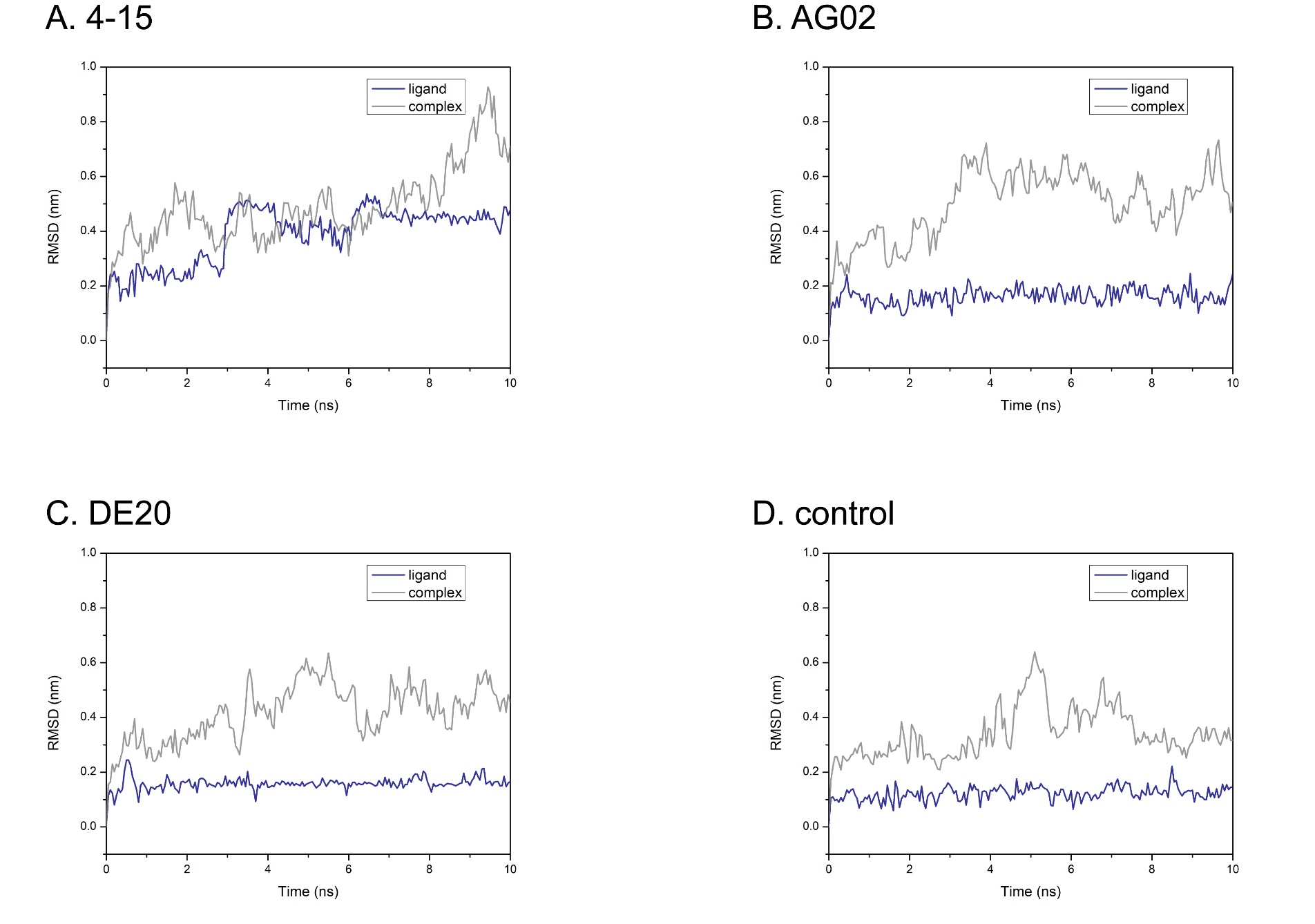


## Figure S9. RMSD of the ligand and the complex. (A) 4-15, (B) AG02 mutant, (C) DE20 mutant, (D) TAK-875.


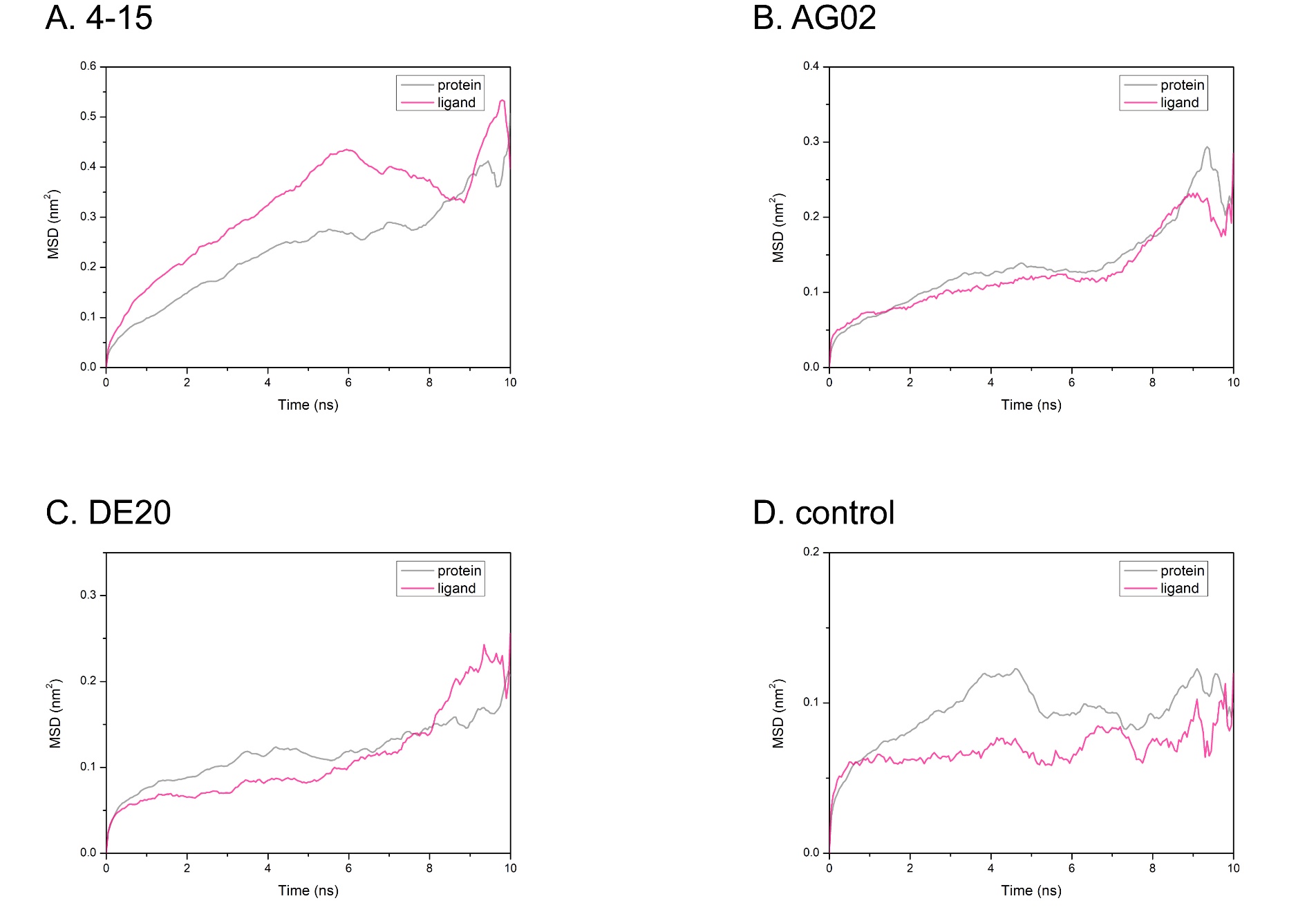


## Figure S10. MSD of the ligand and GPR40 in the complex. (A) 4-15, (B) AG02 mutant, (C) DE20 mutant, (D) TAK-875.


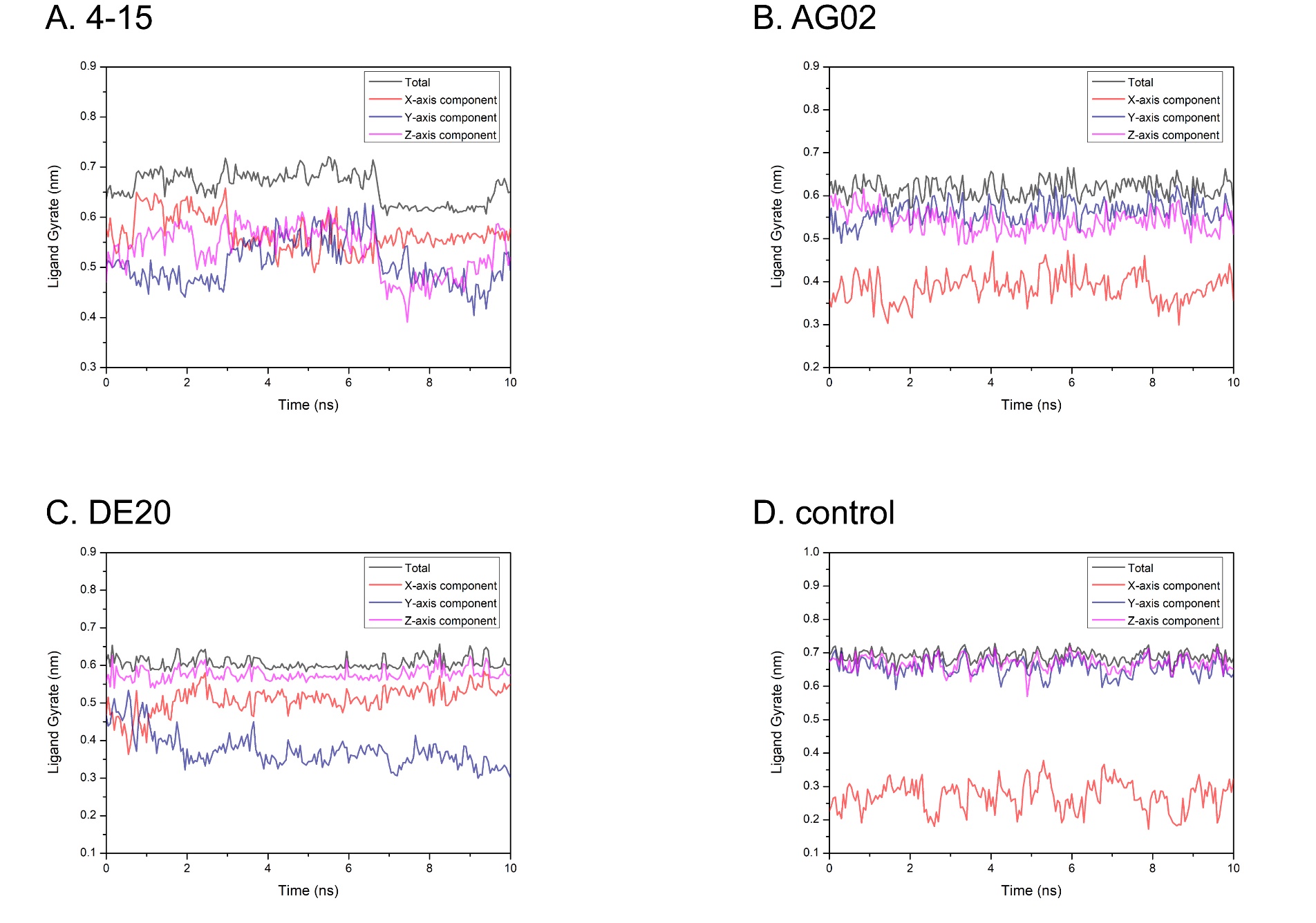


## Figure S11. Gyrate of the ligand in the complex. (A) 4-15, (B) AG02 mutant, (C) DE20 mutant, (D) TAK-875.


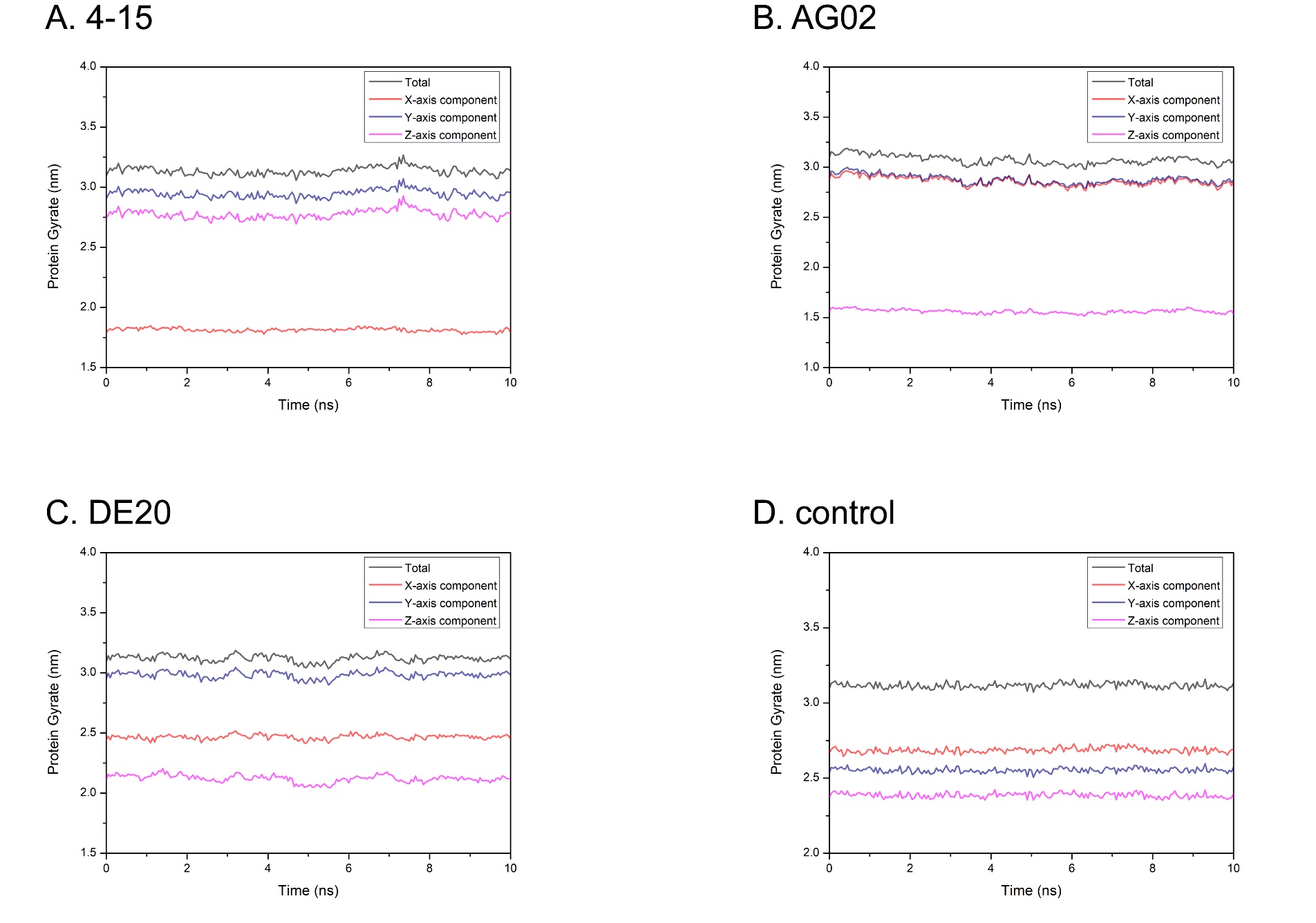


## Figure S12. Gyrate of the GPR40 in the complex. (A) 4-15, (B) AG02 mutant, (C) DE20 mutant, (D) TAK-875.


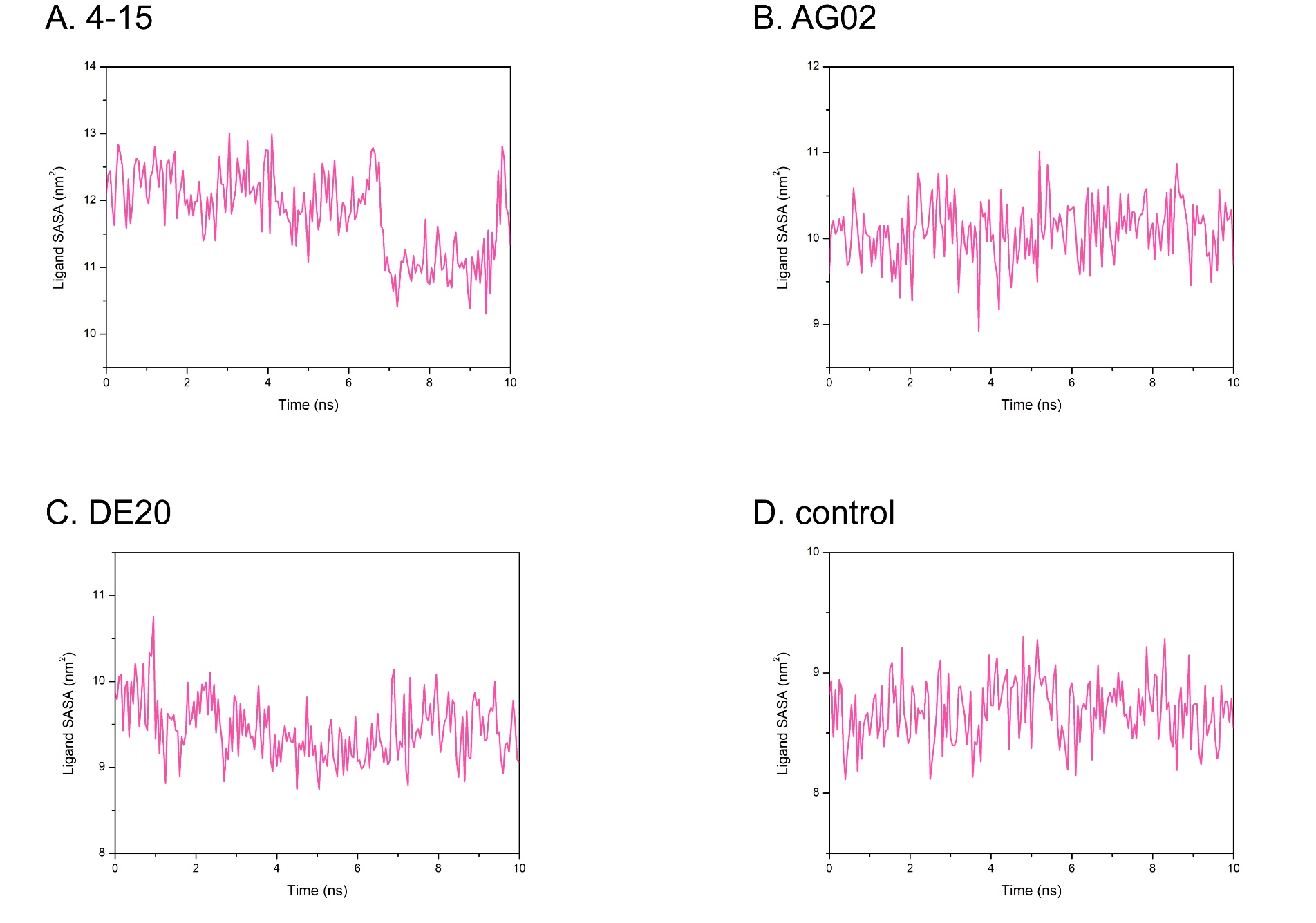


## Figure S13. SASA of the ligand in the complex. (A) 4-15, (B) AG02 mutant, (C) DE20 mutant, (D) TAK-875.


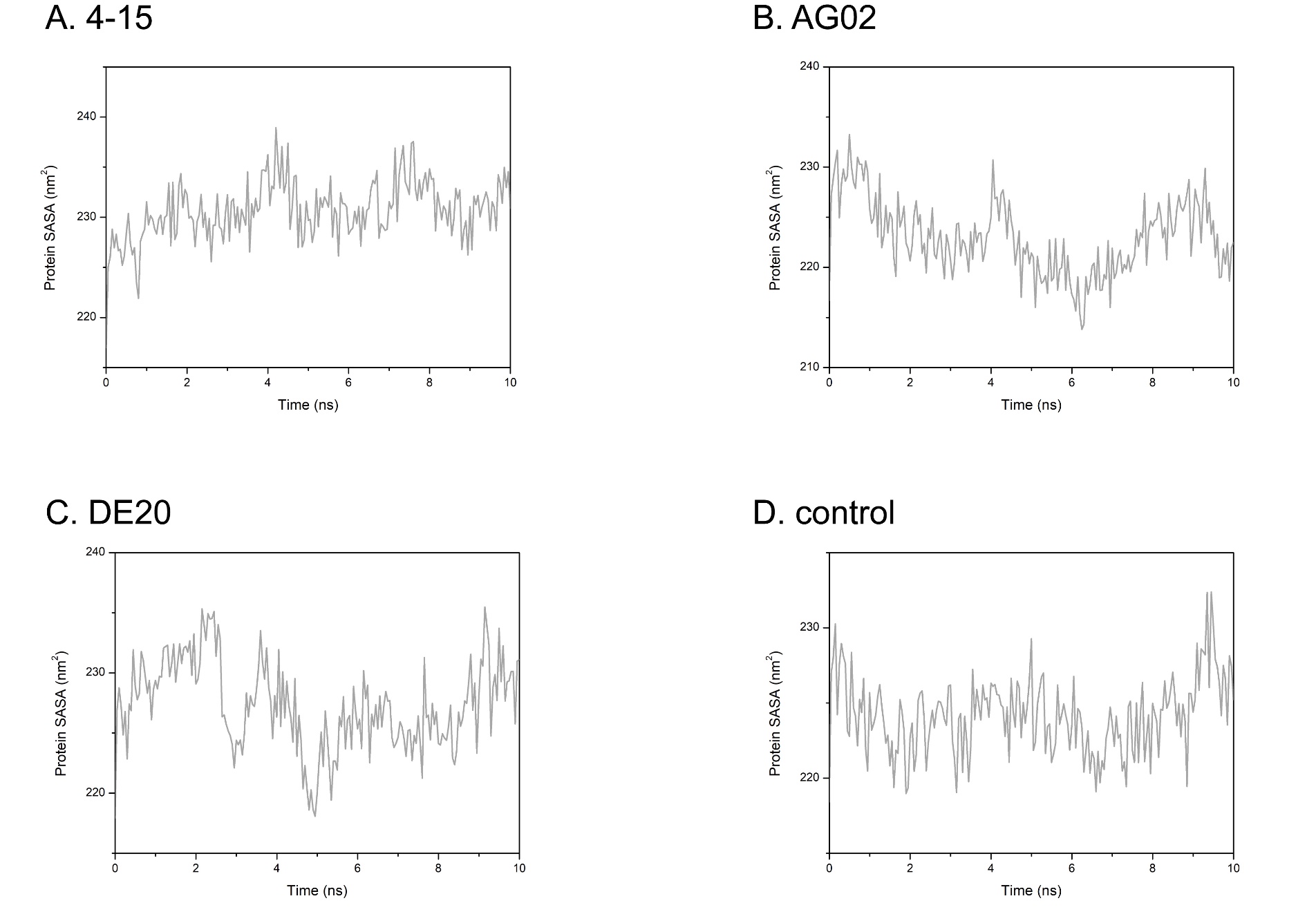


## Figure S14. SASA of the GPR40 in the complex. (A) 4-15, (B) AG02 mutant, (C) DE20 mutant, (D) TAK-875.


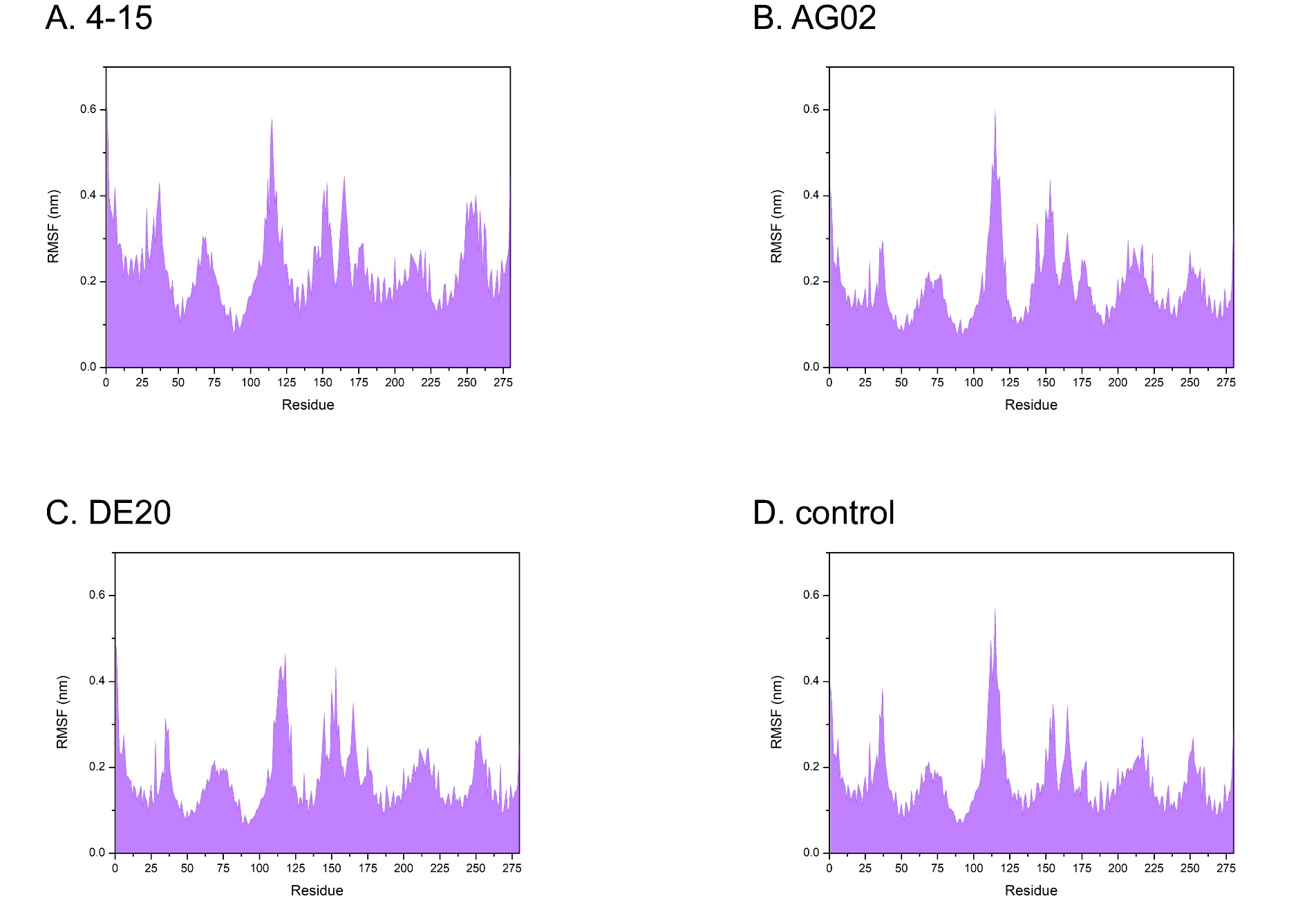


## Figure S15. RMSF of the GPR40 in the complex. (A) 4-15, (B) AG02 mutant, (C) DE20 mutant, (D) TAK-875.


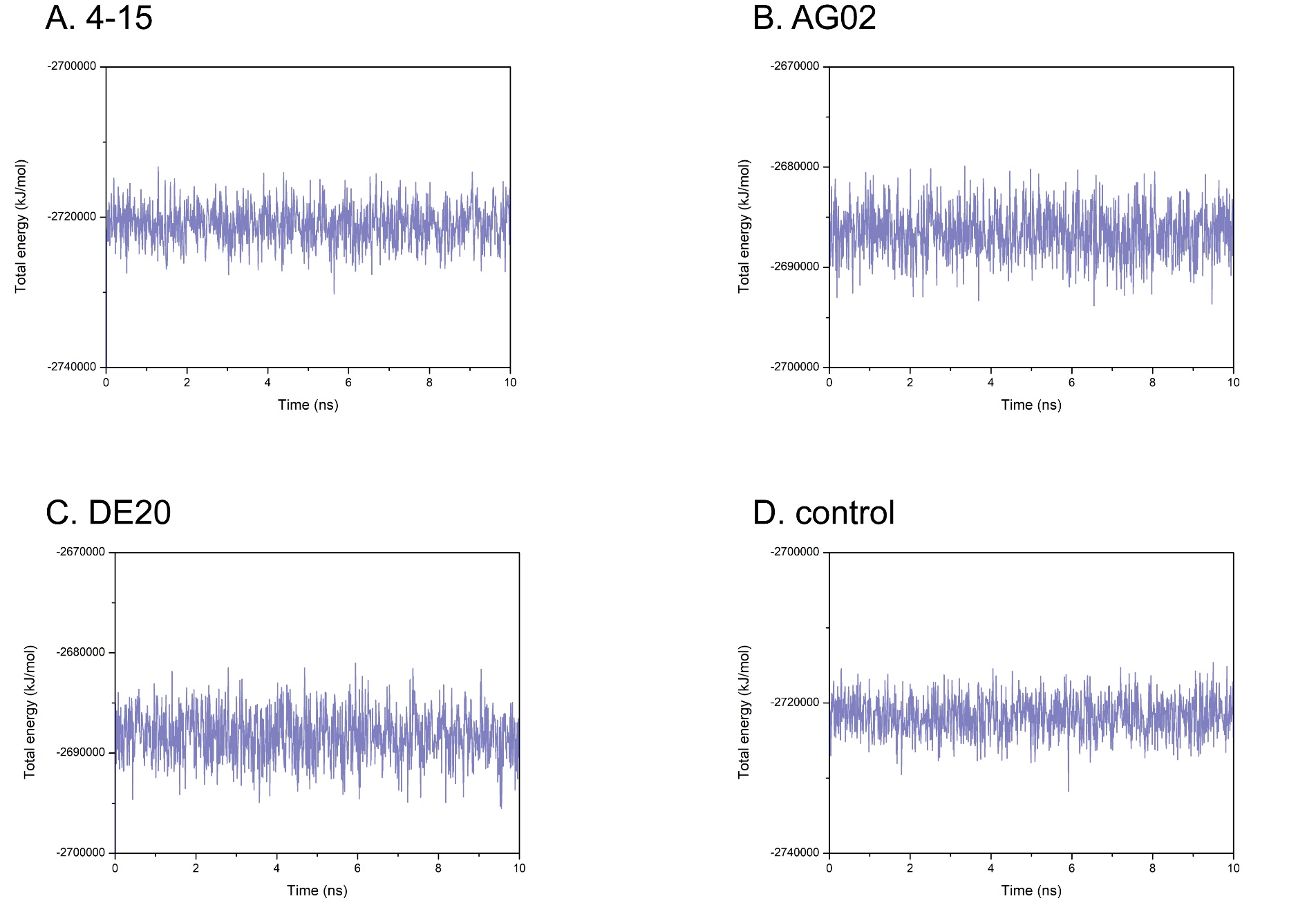


## Figure S16. Total energy of the complex. (A) 4-15, (B) AG02 mutant, (C) DE20 mutant, (D) TAK-875.
